# Synthesis and Coordination Behavior of a New Hybrid Bidentate Ligand with Phosphine and Silylene Donors

**DOI:** 10.1002/chem.202003513

**Published:** 2020-12-14

**Authors:** Mohd Nazish, Mujahuddin M. Siddiqui, Samir Kumar Sarkar, Annika Münch, Christina M. Legendre, Regine Herbst‐Irmer, Dietmar Stalke, Herbert W. Roesky

**Affiliations:** ^1^ Institut für Anorganische Chemie Universität Göttingen Tammannstraße 4 37077 Göttingen Germany

**Keywords:** aluminium, boron, copper, phosphane, silylene

## Abstract

This work describes the synthesis and coordination behavior of a new mixed‐donor ligand PhC(N*t*Bu)_2_SiC_6_H_4_PPh_2_ (**1**) containing both silylene and phosphine donor sites. Ligand **1** was synthesized from a reaction of *ortho*‐lithiated diphenylphosphinobenzene (LiC_6_H_4_PPh_2_) with chlorosilylene (PhC(N*t*Bu)_2_SiCl). Treatment of **1** with Se and GeCl_2_ resulted in Si^IV^ compounds **2** and **3** by selective oxidation of the silylene donor. This strong σ‐donor ligand induces dissociation of CuCl and PhBCl_2_ leading to formation of ionic complexes **4** and **5** respectively. The reaction of **1** with ZnCl_2_ and AlCl_3_ resulted in the formation of chelate complexes **5** and **7**, respectively, while treatment with EtAlCl_2_ and GaCl_3_ forms monodentate complexes **8** and **9**. X‐ray analysis of **4** showed that the copper is in the spiro center of the two five‐membered rings. Moreover, the copper(I)chloride has not been oxidized but dissociates to Cu^+^ and [CuCl_2_]^−^. All the compounds are well characterized by mass spectrometry, elemental analysis, NMR spectroscopy, and single‐crystal X‐ray diffraction studies.

## Graphical abstract

**A ligand with different Lewis base** donor sites of phosphorus(III) and silicon(II). The ligands are arranged in 1,2‐position at the benzene ring to generate mainly five‐membered ring compounds containing a metal atom. The exceptions of the compounds show clearly that the metal is preferentially coordinated to the silylene atom rather than to the phosphane site of the ligand.

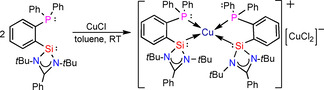

## Introduction

The structure, reactivity and catalytic activities of the main group and transition metal complexes are strongly related to the steric and electronic factors of the coordinating ligand. Over the last few decades, highly reactive compounds with low‐valent silicon, phosphorus and other main‐group elements have been isolated and structurally characterized.[^1^, ^2^, ^3^, ^4^] These compounds have attracted significant attention due to their exciting electronic structures, small molecules activating capability at ambient conditions and catalytic properties in organic transformations.[^1c^, ^5^] Most of these novel achievements were due to the suitable design and synthesis of ligands which stabilize species with low‐valent elements. N‐heterocyclic carbenes (NHCs),^[6]^ cyclic alkyl(amino) carbenes (cAACs)^[7]^ and N‐heterocyclic silylenes (NHSis)^[8]^ are the most successful and commonly used ligand systems to stabilize compounds with low valent elements.

**Scheme 1:**
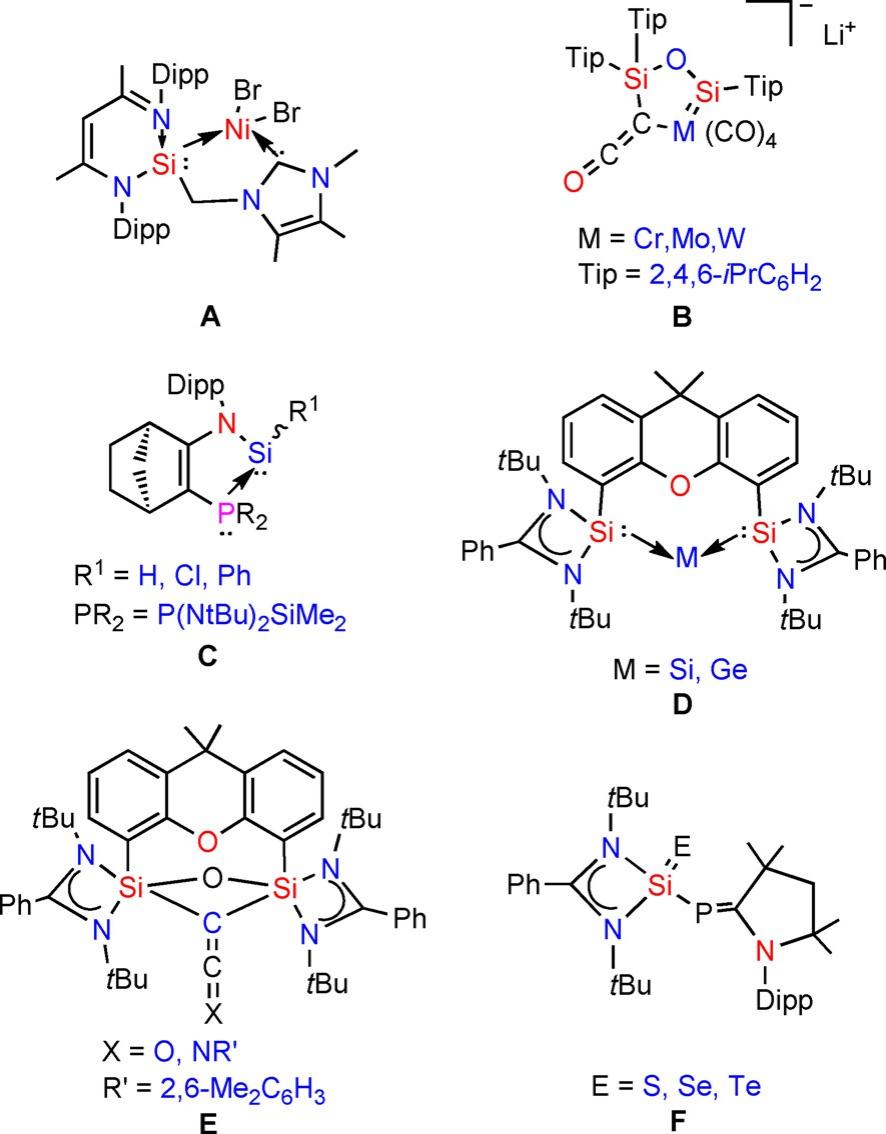
Reported silylene‐, bis(silylene)‐, diphenylphosphine‐, and phosphinidene‐based ligand systems.

Silylenes have been effectively used as stabilizing ligands in low valent main‐group elements, transition metals and for the metal free activation of robust bonds in small molecules. Few examples that illustrate the possibility to form new silylene ligands bearing an additional coordination site were published in 2016 by S. Khan et al. They have used silylene phosphine based bidentate ligand and showed the coordination of silylene with gold.^[9]^ Very recently, Stalke et al. synthesized a side‐arm functionalized silylene ligand and their transition metal complexes.^[10]^ In 2014, Driess et al. reported silylene coordinated mono‐ and dinuclear copper(I) complexes.^[11]^ The same group has synthesized mixed silylene‐carbene chelate ligands to stabilize transition metal. Furthermore they have studied the catalytic activity of **A** (Scheme 1).^[12]^ Scheschkewitz et al. have shown the synthesis of ketenyl‐ligated metal‐silylene complexes of group 6 (**B**).^[13]^ Li and co‐workers recently have synthesized bis(silylene)‐based SiC^(sp3)^Si pincer ligand and studied its coordination chemistry with Fe^0^ under Ar and N_2_.^[14]^ Tilley and others have reported chemical and catalytic properties of silylene coordinated transition metal complexes.^[15]^ In 2011, Kato et al. published the synthesis of a stable and isolable tricoordinate silicon(II) hydride (**C**), stabilized by a phosphine ligand.^[16]^ The same research group has also delivered silylene based complexes to stabilize transition metal and a highly electron rich carbon(0).^[17]^ Driess and co‐workers have successfully used bis(silylene) to stabilize zero valent silicon and germanium, respectively (**D**).^[18]^ Very recently, the same group reported homocoupling of CO and isocyanide, mediated by a bis(silylene) ligand (**E**).^[19]^

Recent studies indicated that catalytic efficiencies of various catalytic reactions can be increased by silylene ligands due to their stronger σ‐donation ability along with cooperative effects of the divalent silicon atoms in the process of catalysis. In many cases, it was found that silylenes function as powerful σ‐donor ligands surpassing the activities of analogous phosphine‐ and NHC‐based metal complexes.^[20]^

Mixed donor ligand systems allow fine tuning of the steric and electronic properties at the metal center by choosing appropriate donor atoms. Such a fine tuning can have a significant impact on the chemo‐, regio‐ and stereo selectivities in metal‐mediated catalytic transformations of organic substrates. Our group has longstanding interest in the synthesis and reactivity of silylenes and phosphinidenes. In 2017, we reported the synthesis of cAAC anchored silylene‐phosphinidene synthesized by the reaction of silylene monochloride and chlorophosphinidene, followed by reduction with KC_8_.^[21]^

In the subsequent studies, we found that the reaction of silylene‐phosphinidene with chalcogens resulted in the selective formation of silicon bonded chalcogen phosphinidenes (**F**).^[22]^ Silylenes were found to be strong σ‐donor ligands in comparison with phosphinidenes. We envisioned the synthesis of a mixed donor ligand system with phosphane and silylene donors to study its electronic properties and coordination behavior. The present work results from our effort to study the reactivity of such mixed donor ligand with transition metals as well as main group elements. Herein, we present the synthesis of a novel hybrid phosphane‐silylene ligand (**1**) and its coordination properties with various transition metals and main group elements.

## Results and Discussion

**Scheme 2:**
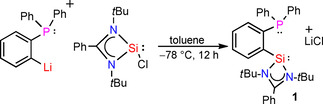
Synthesis of phosphane‐silylene ligand (**1**).

**Figure 1:**
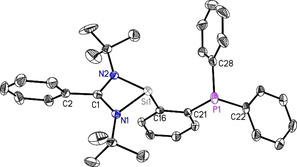
Crystal structure of PhC(N*t*Bu)_2_SiC_6_H_4_PPh_2_ (**1**). The anisotropic displacement parameters are depicted at the 50 % probability level. Hydrogen atoms are omitted for clarity. Selected bond lengths [Å] and angles [°]: Si1−N1 1.8595(13); Si1−N2 1.8696(13); Si1−C16 1.9315(15); P1−C21 1.8452(16); P1−C22 1.8384(17); P1−C28 1.8283(18); N1‐Si1‐C16 100.02(6); N2‐Si1‐C16 99.99(7); N1‐Si1‐N2 69.28(6).

In 2010, our group reported a high yielding method for the synthesis of *N*,*N*‐di(*tert*‐butyl)amidinato chlorosilylene.^[23]^ It is a versatile reagent, used in the synthesis of diverse silylene based ligands with different spacer lengths, specific structural and electronic features. We envisioned the use of chlorosilylene precursor for the synthesis of phosphane‐silylene ligand system. *ortho*‐lithiated diphenylphosphane and *N*,*N*‐di(*tert*‐butyl)amidinato chlorosilylene were treated in toluene (−78 °C to room temperature) and resulted in a purple solution. The solution was filtered and concentrated to 10 mL and stored at −30 °C in a freezer for crystallization. Colorless crystals of compound **1** were obtained after 24 hours (Scheme 2). The ^31^P NMR spectrum of compound **1** shows a single resonance at −11.2 ppm (Figure S2 in the Supporting Information), a upfield shift of 6.2 ppm compared to the starting material (−5.0 ppm). The ^29^Si NMR spectrum of compound **1** exhibits a doublet at 18.52 ppm with a ^3^ *J*_Si‐P_ coupling of 410 Hz which is significantly downfield shifted compared to the chlorosilylene (−96.8 ppm). Compound **1** crystallizes in the monoclinic space group *C*2/*c* with one single molecule of **1** in the asymmetric unit. Analysis via single‐crystal X‐ray diffraction additionally reveals a distorted tetrahedral environment around silicon, accommodating two inequivalent nitrogen donor atoms (Si1–N1: ca. 1.86 and Si1‐N2: ca. 1.87 Å). The carbon on the phosphine moiety (C16 on Figure 1) and the lone pair of electrons on Si1 are available for further chelation.

**Figure 2:**
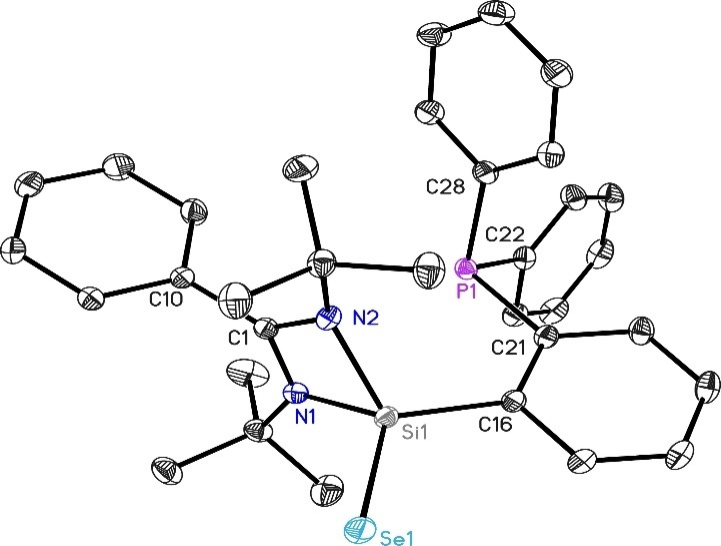
Crystal structure of PhC(N*t*Bu)_2_SiSeC_6_H_4_PPh_2_ (**2**). The anisotropic displacement parameters are depicted at the 50 % probability level. Hydrogen atoms are omitted for clarity. Selected bond lengths [Å] and angles [°]: Si1−Se1 2.1330(5); Si1−N1 1.8224(9); Si1−N2 1.8249(9); Si1−C16 1.8847(10); P1−C21 1.8439(10); P1−C22 1.8367(10); P1−C28 1.8249(11); N1‐Si1‐C16 116.50(4); N2‐Si1‐C16 111.61(4); N1‐Si1‐N2 72.04(4).

An equimolar reaction of **1** with elemental selenium in toluene at room temperature resulted in the selective oxidation of silylene donor to form **2**. The ^31^P NMR spectrum of **2** shows a single NMR resonance at −12.3 ppm (Figure S6). The structure of **2** was confirmed by single‐crystal X‐ray diffraction. Compound **2** crystallizes in the monoclinic space group *C*2/*c* as well, with one molecule of **2** and two solvent molecules of THF in the asymmetric unit. Selenium binds exclusively with silicon, at 2.1330(5) Å bond length. The greater distance between selenium and phosphorus atoms, about 5.6 Å, as well as the orientation of the phosphine moiety strongly invalidate the hypothesis of additional bonding (Figure 2).

**Scheme 3:**
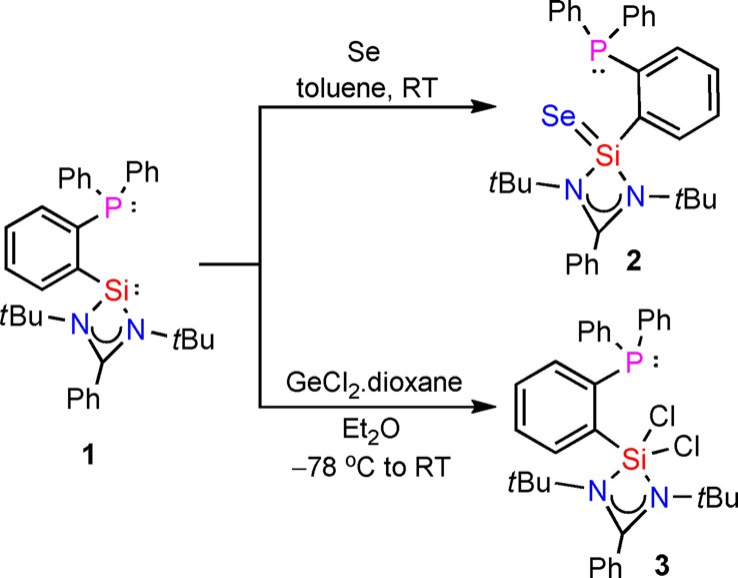
Reactions of **1** with Se and GeCl_2_.

**Figure 3:**
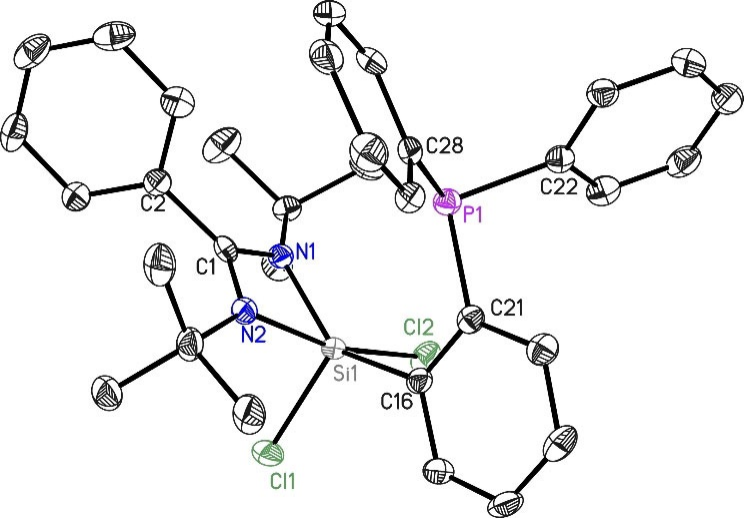
Crystal structure of PhC(N*t*Bu)_2_SiCl_2_C_6_H_4_PPh_2_ (**3**). The anisotropic displacement parameters are depicted at the 50 % probability level. Hydrogen atoms are omitted for clarity. Selected bond lengths [Å] and angles [°]: Si1−Cl1 2.0934(10); Si1−Cl2 2.2113(11); Si1−N1 1.832(2); Si1−N2 1.937(2); Si1−C16 1.896(3); P1−C21 1.828(3); P1−C22 1.837(3); P1−C28 1.838(3); N1‐Si1‐C16 140.05(11); N2‐Si1‐C16 97.41(10), N1‐Si1‐N2 69.42(9).

Surprisingly, the reaction of **1** with GeCl_2_ did not yield a germanium complex but resulted in the oxidation of silylene moiety to yield the dichlorinated compound **3** (Scheme 3). The ^31^P NMR of **3** shows single NMR resonance at 12.2 ppm (Figure S10) which shows a downfield shift of 23.4 ppm, when compared with ligand **1**. The formation of compound **3** was further confirmed by single crystal X‐ray diffraction studies, which crystallizes in the monoclinic space group *P*2_1_/*n* without any additional solvent molecules in the asymmetric unit. The penta‐coordinated silicon features a distorted environment comparable to a transition state between trigonal bipyramidal and square pyramidal, due to the rigid structure of the amidinate (Figure 3).

**Scheme 4:**
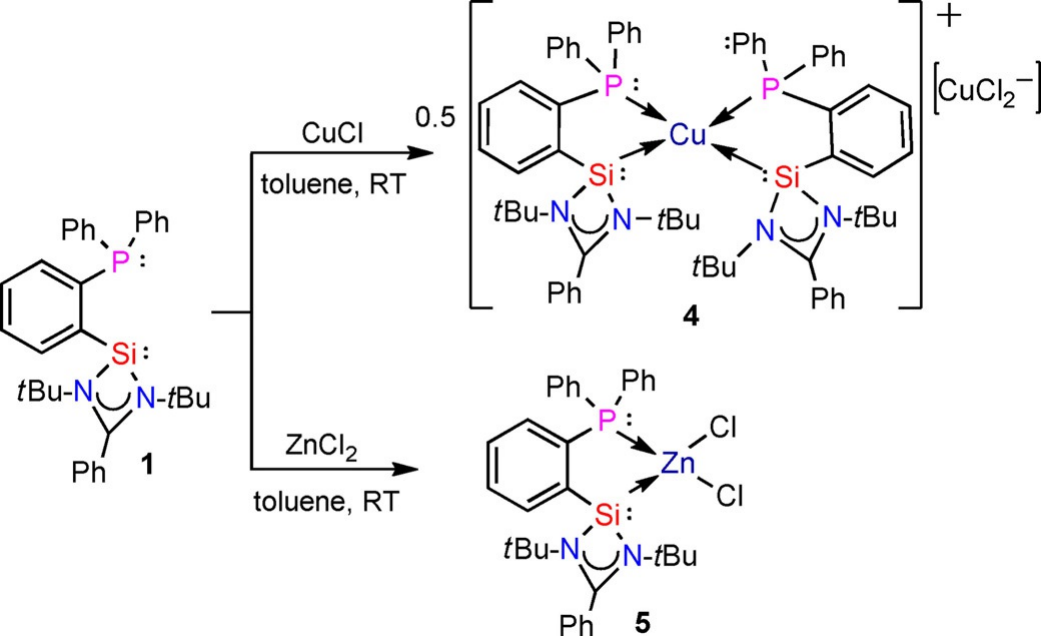
Reactions of **1** with CuCl and ZnCl_2_.

The reaction of **1** with CuCl in toluene at room temperature leads to the dissociation of CuCl resulting in the formation of the diamagnetic complex [L_2_Cu]^+^[CuCl_2_]^−^ (**4**). (Scheme 4). We use several analysis methods to determine the magnetic state of compound **4**: a dismutation of Cu^I^ in Cu^0^ and Cu^II^ would be assessed paramagnetic, whereas simple dissociation of Cu^I^ would remain diamagnetic. ^1^H and ^13^C NMR measurements are measurable in the standard chemical shift range. Moreover, a toluene solution of **4** is found EPR silent, which is also in agreement with SQUID analysis results, invalidating the hypothesis of the formation of paramagnetic species.

**Figure 4:**
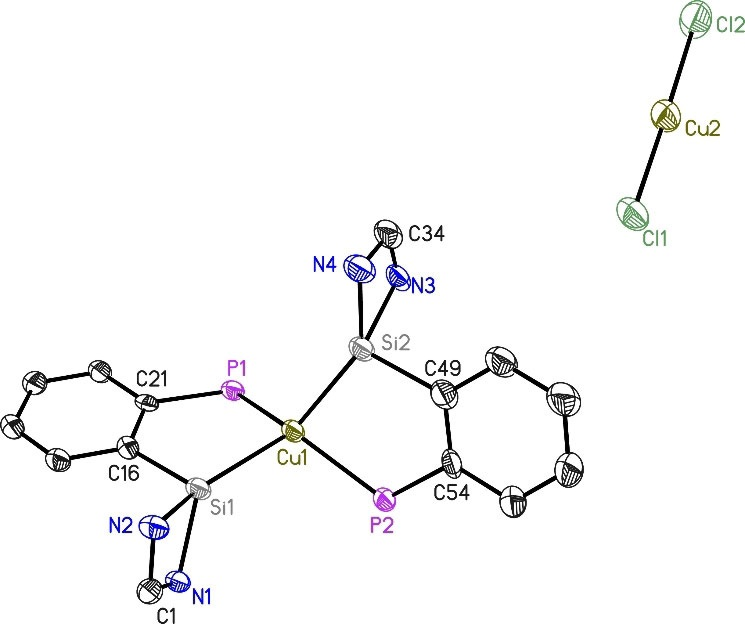
Crystal structure of one formula unit of [L_2_Cu]^+^[CuCl_2_]^−^ (**4**). The anisotropic displacement parameters are depicted at the 50 % probability level. Phenyl groups, *t*Bu groups and hydrogen atoms are omitted for clarity. Selected bond lengths [Å] and angles [°]: Si1−Cu1 2.2637(16); Si2−Cu1 2.2728(16); P1−Cu1 2.2982(15); P2−Cu1 2.2807(16); Si1−N1 1.867(4); Si1−N2 1.838(5); Si2−N3 1.857(4); Si2−N4 1.841(5); Si1−C16 1.883(5); P1−C21 1.832(5); P2−C54 1.840(5); Si1‐Cu1‐Si2 135.45(7); Si1‐Cu1‐ P2 111.09(6); Si2‐Cu1‐P2 87.34(6); Si1‐Cu1‐P1 87.68(6); Si2‐Cu1‐P1 111.07(6); N1‐Si1‐C16 101.2(2); N2‐Si1‐C16 103.9(2); N1‐Si1‐N2 70.0(2).

The ^31^P NMR of compound **4** shows single resonance at −8.7 ppm (Figure S14) which exhibits downfield shift of 2.5 ppm when compared with ligand **1**. Single crystals of **4** were obtained by storing a concentrated toluene solution at −30 °C for 2 days in a freezer. Compound **4** crystallizes in space group *P* with two units of [L_2_Cu]^+^[CuCl_2_]^−^ and nine toluene molecules. The crystal structure reveals that the copper is in the spiro center of the two five‐membered rings (Figure 4). The central copper atom is featuring a distorted tetrahedral geometry, possibly due to the steric arrangement of the phosphine phosphorus and the *tert*‐butyl groups. Comparison with already reported Cu−X bond lengths (X=P, Si, Cl; see the Supporting Information) also suggests that compound **4** is diamagnetic and that copper chloride is not oxidized, but rather dissociates to Cu^+^ and [CuCl_2_]^−^. The Si1−Cu1 and Si2−Cu1 distances are 2.264 and 2.273 Å, which is comparable to other Si−Cu distances in copper(I) complexes^[24]^ (see the Supporting Information). The P1−Cu1 and P2−Cu1 bonds are quite long, about 2.28–2.3 Å, which is actually comparable to Cu^0^−P bond lengths. However, although slightly larger than average, it still falls in the acceptable range of 2.2 to 2.4 Å for copper(I) complexes.[^25a^, ^25b^] Additionally, the [CuCl_2_]^−^ unit features two short Cu−Cl bonds of 2.099 and 2.104 Å. This distance is closer to the one of copper monochloride than average distances for Cu^II^−Cl about 2.3 Å (see the Supporting Information). It is not entirely excluded that Cu^I^ remains unoxidized but the presented analysis overall strongly suggests that the obtained complex **4** is diamagnetic.

**Figure 5:**
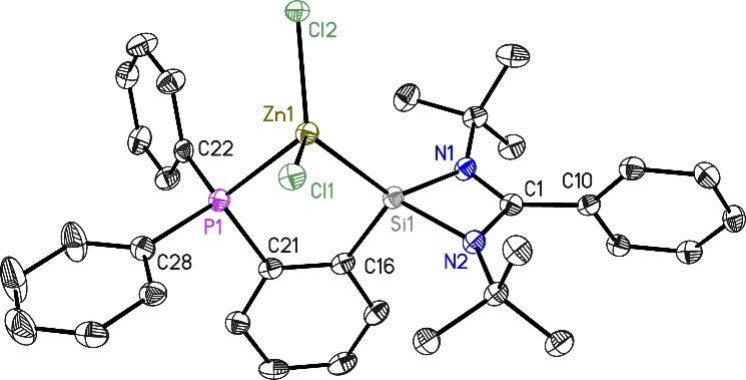
Molecular structure of PhC(N*t*Bu)_2_SiZnCl_2_C_6_H_4_PPh_2_ (**5**). The anisotropic displacement parameters are depicted at the 50 % probability level. Hydrogen atoms are omitted for clarity. Selected bond lengths [Å] and angles [°]: Si1−Zn1 2.3947(8); Zn1−P1 2.4812(8); Zn1−Cl1 2.2661(7); Zn1−Cl2 2.2463(10); Si1−N1 1.8231(19); Si1−N2 1.820(2); Si1−C16 1.879(2); P1−C21 1.838(2); P1−C22 1.821(2); P1−C28 1.821(2); Si1‐Zn1‐P1 85.41(2); Zn1‐P1‐C21 104.78(8); Zn1‐P1‐C22 115.09(8); Zn1‐P1‐C28 120.15(8); Zn1‐Si1‐C16 106.14(7); N1‐Si1‐N2 71.88(9); Zn1‐Si1‐N1 132.52(7); Zn1‐Si1‐N2 125.27(6).

The reaction of **1** with ZnCl_2_ leads to the formation of a chelate complex LZnCl_2_ (L=PhC(N*t*Bu)_2_SiC_6_H_4_PPh_2_; **5**) where the ligand exhibits chelating mode of coordination. The ^31^P NMR of **5** shows a single resonance at −26.7 ppm (Figure S17) which exhibits a upfield shift of 15.5 ppm in comparison with the ligand **1**. Crystals suitable for single‐crystal X‐ray analysis are obtained from THF solution after two weeks in the freezer at −30 °C. Compound **5** crystallizes in the monoclinic space group *P*2_1_/*c*, presenting one single molecule of **5** in the asymmetric unit. In this complex, both phosphine and silylene moieties are participating in metal coordination (Figure 5). The Zn1−Si1 bond is somewhat shorter than the Zn1−P1 bond, indicative of a preference for the silylene moiety. The slight distortion of the tetrahedral geometry around the Zn atom is due to the P1‐Zn1‐Si1 angle of about 85°, shortened to adapt to the backbone rigid structure.

Both the complexes **4** and **5** are crystalline solids, soluble in common organic solvents, and stable at room temperature under inert atmosphere. Melting points of complexes **4** and **5** were found to be 280 and 270 °C, respectively.

**Scheme 5:**
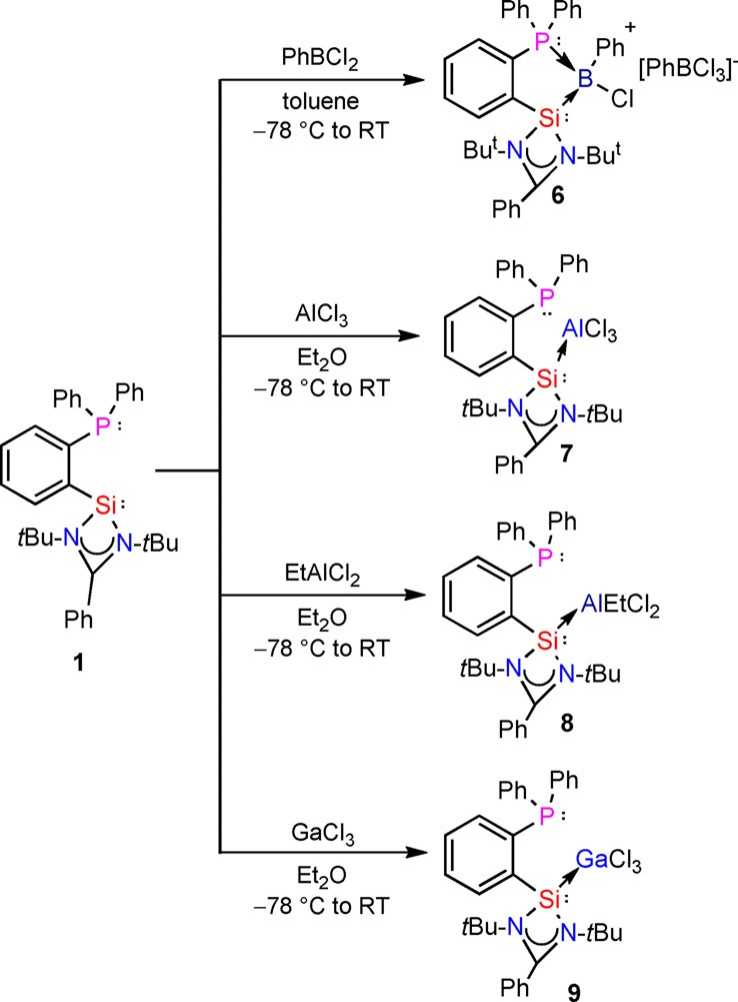
Reactions of **1** with PhBCl_2_, AlCl_3_, EtAlCl_2_ and GaCl_3_.

**Figure 6:**
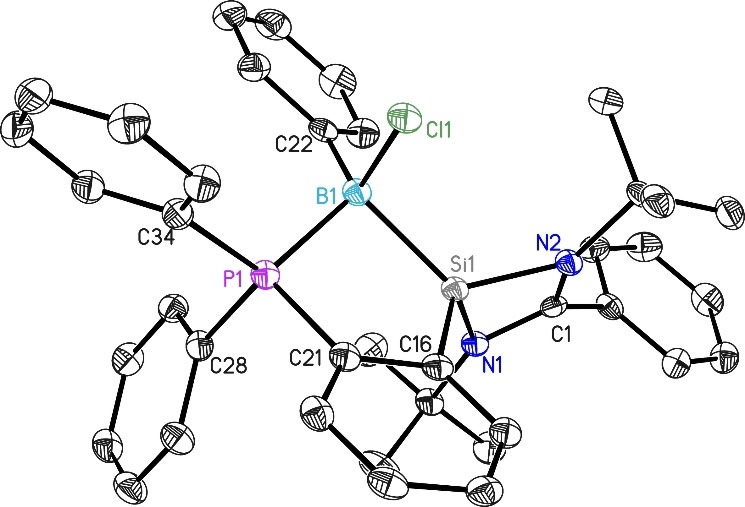
Molecular structure of [LBPhCl]^+^[PhBCl_2_Cl]^−^ (**6**). The anisotropic displacement parameters are depicted at the 50 % probability level. Hydrogen atoms and [PhBCl_2_Cl]^−^ are omitted for clarity. Selected bond lengths [Å] and angles [°]: Si1−B1 2.018(2); P1−B1 1.982(2); B1−Cl1 1.904(2); Si1−N1 1.8120(17); Si1−N2 1.7945(17); Si1−C16 1.855(2); P1−C21 1.814(2); P1−C28 1.8076(19); P1−C34 1.803(2); Si1‐B1‐P1 95.33(10); B1‐P1‐C21 101.57(9); B1‐Si1‐C16 100.86(9); N1‐Si1‐N2 73.09(8).

Similar to the formation of the copper complex **4**, ligand **1** reacted with PhBCl_2_ in toluene at −78 °C. This leads to a quite unusual dissociation of PhBCl_2_ into the product [LBPhCl]^+^[PhBCl_3_]^−^ (**6**; Scheme 5) as confirmed by single‐crystal X‐ray diffraction. Crystals were obtained after two weeks at room temperature in toluene. Compound **6** crystallizes in the triclinic centrosymmetric space group *P*‐1, with no residual solvent molecule in the asymmetric unit. Interestingly, the ligand chelates the small boron atom with both coordinating moieties. The boron center is almost perfectly tetrahedral, and its coordination to the ligand induces a distortion on the phosphine moiety instead of modifying the tetrahedral geometry on the boron atom (Figure 6). This suggests that the small size of the boron atom enables it to fit inside of the chelating moiety, however through a shrinking of the host pocket. All bonds to boron are indeed shorter than with any other guest atom of the present study (see Table 1 for a detailed comparison).

**Table 1:** Comparison of selected distances and bond angles for **1**–**9**.

	Distances [Å]					Bond angles [°]			
	X	X−Si	Si−N	X−P	Distance X from SiPCC plane	Si‐X‐P	N‐Si‐X	N‐Si‐C16/C49	N1‐Si‐N2
**1**	–		1.8589(13)	–				100.02(6)	69.28(6)
			1.8698(13)					99.99(7)	
**2**	Se	2.1330(5)	1.8224(9)	–	0.0768(6)		116.19(3)	116.50(4)	72.04(4)
			1.8249(9)				117.52(3)	111.61(4)	
**3**	Cl1		1.832(2)	–	0.3184(19)			140.05(11)	69.42(9)
			1.937(2)					97.41(10)	
**4**	Cu	2.2637(16)	1.867(4)	2.2982(15)	0.506(4)	87.34(6)	128.60(17)	101.2(2)	70.0(2)
		2.2728(16)	1.838(5)			111.09(6)	139.11(15)	103.9(2)	70.5(2)
			1.857(4)	2.2807(16)	0.495(4)	86.68(6)	128.23(16)	100.1(2)	
			1.841(5)			111.07(6)	140.41(17)	103.4(2)	
**5**	Zn	2.3947(8)	1.8231(19)	2.4812 (8)	0.4105(19)	85.41(2)	132.52(7)	109.55(9)	71.88(9)
			1.820(2)				125.27(6)	106.73(10)	
**6**	B	2.018(2)	1.8120(17)	1.982 (2)	0.751(2)	95.33(10)	125.06(9)	116.98(8)	73.09(8)
			1.7945(17)				123.11(8)	117.77(9)	
**7**	Al	2.4509(8)	1.8176(12)	3.5920(7)	0.3186(16)		108.19(4)	107.09(6)	72.11(5)
			1.8149(12)				111.08(4)	110.07(6)	
**8**	Al	2.4988(14)	1.820(3)	3.3587(14)	0.115(4)		114.44(9)	105.92(14)	71.43(12)
			1.826(3)				118.29(10)	104.60(13)	
**9**	Ga	2.3904(6)	1.8053(12)	3.5640(6)	0.3189(15)		109.68(4)	112.04(6)	72.66(5)
			1.8073(12)			107.08(4)	108.87(6)

Surprised by the ability of ligand **1** in the dissociation of CuCl and PhBCl_2_, we investigated the reaction with Al salts for the formation of ionic aluminium compounds.

**Figure 7:**
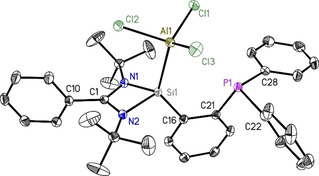
Molecular structure of PhC(N*t*Bu)_2_SiAlCl_3_C_6_H_4_PPh_2_ (**7**). The anisotropic displacement parameters are depicted at the 50 % probability level. Hydrogen atoms are omitted for clarity. Selected bond lengths [Å] and angles [°]: Si1−Al1 2.4509(8); Al1−Cl1 2.1321(6); Al1−Cl2 2.1751(6); Al1−Cl3 2.1392(6); Si1−N1 1.8176(12); Si1−N2 1.8149(12); Si1−C16 1.8705(14); P1−C21 1.8464(15); P1−C22 1.8412(15); P1−C28 1.8329(15); Al1‐Si1‐C16 131.98(5); Al1‐Si1‐N1 108.19(4); Al1‐Si1‐N2 111.08(4); N1‐Si1‐N2 72.11(5).

The reaction of **1** with AlCl_3_ in diethyl ether at −78 °C resulted in the formation of compound **7**. Crystals are grown from CH_2_Cl_2_ at 0 °C. Compound **7** crystallizes in the monoclinic space group *P*2_1_/*c* with one molecule of **7** and one molecule of CH_2_Cl_2_ in the asymmetric unit. The aluminium atom is in a distorted tetrahedral environment: all Cl‐Al‐Cl angles are about 109°, but the Si‐Al‐Cl2 angle is remarkably smaller (98.77(3)°), probably to allow an additional weaker interaction between Al and the pending phosphine group (Figure 7). Single‐crystal X‐ray analysis also reveals that the Al atom is bounded to the silylene moiety while the phosphorus donor atom is “only” oriented towards the metal. This shows as expected that silylene is a stronger σ‐donor ligand compared to the P atom of phosphane.

**Figure 8:**
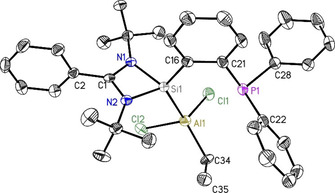
Molecular structure of (**8**). The anisotropic displacement parameters are depicted at the 50 % probability level. Hydrogen atoms are omitted for clarity. Selected bond lengths [Å] and angles [°]: Si1−Al1 2.4988(14); Al1−Cl1 2.215(3); Al1−Cl2 2.2123(14); Al1−C34 1.952(5); Si1−N1 1.820(3); Si1−N2 1.826(3); Si1−C16 1.880(3); P1−C21 1.837(4); P1−C22 1.825(3); P1−C28 1.829(3); Al1‐Si1‐C16 127.90(11); Al1‐Si1‐N1 114.44(9); Al1‐Si1‐N2 118.29(10); N1‐Si1‐N2 71.43(12).

In the same spirit, the reaction of **1** with EtAlCl_2_ and GaCl_3_ in diethyl ether resulted in the formation of compounds **8** and **9**. Compound **8** crystallizes in the orthorhombic space group *P*2_1_2_1_2_1_ with one molecule of **8** in the asymmetric unit. The Al^III^ is placed in a tetrahedral environment, with a deviation comparable to **7** (one angle is 95.53(5)° and the others are about 109°) to leave some space for the phosphino group oriented towards the aluminium atom. The distance between Al and P atoms is shorter than in **7** (3.36 and 3.59 Å respectively), which is probably due to the additional flexibility given by the ethyl moiety in comparison with the halide (Figure 8).

Single‐crystal X‐ray analysis reveals that compounds **7** and **9** are isostructural. Indeed, the gallium atom is also exhibiting a tetrahedral coordination environment, with similar angle values. A deviation from perfectly tetrahedral geometry is again observed (the av. angle value from the sum of all angles is 109° but the angle values range from 100 to 118°) and attributed to the structural adaptation at the metal center in order to acknowledge the preferred orientation of the phosphorus towards the aluminium atom. The distances between the two atoms is similar to those of compounds **7** and **8**, about 3.56 Å.

**Figure 9:**
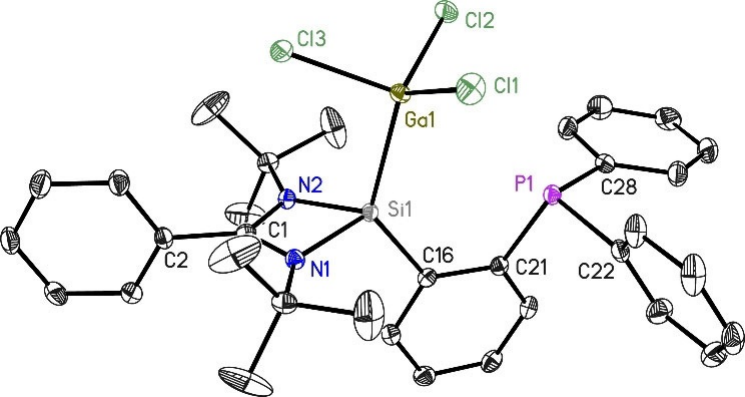
Molecular structure of PhC(N*t*Bu)_2_SiGaCl_3_C_6_H_4_PPh_2_ (**9**). The anisotropic displacement parameters are depicted at the 50 % probability level. Hydrogen atoms are omitted for clarity. Selected bond lengths [Å] and angles [°]: Si1−Ga1 2.3904(6); Ga1−Cl1 2.1917(4); Ga1−Cl2 2.1830(5); Ga1−Cl3 2.2425(5); Si1−N1 1.8053(12); Si1−N2 1.8073(12); Si1−C16 1.8627(14); P1−C21 1.8471(14); P1−C22 1.8399(14); P1−C28 1.8315(15); Ga1‐Si1‐C16 131.02(5); Ga1‐Si1‐N1 109.68(4); Ga1‐Si1‐N2 107.08(4); N1‐Si1‐N2 72.66(5).

The ligand **1** coordinates through the silylene moiety in monodentate fashion in compounds **8** and **9** with a noticeable orientation of the phosphane moiety towards Al and Ga, respectively. Thus, considering complexes **2**, **4**, **5**, **7**–**9**, the newly designed hybrid phosphane‐silylene ligand shows different coordination behavior depending on the metal center. Probably due to the higher Lewis acidity of aluminium, the lattice energy for the formation of the [LAlCl_2_]^+^ [AlCl_4_]^−^ salt in **7** (or **8** or **9**) is not high enough. Selected bond lengths and angles for all compounds **1**–**9** are reported in Table 1. Overall trends for bond and angle variations can be summarized as the following. While the oxidation of the silylene results in a shrinking of the Si−N bonds from **1** (mean 1.86 Å) to **2** (mean 1.82 Å), the chlorination cancels the equivalence of the two Si−N bonds in **3** (1.832 and 1.937 Å) influencing the C1−N bonds (1.353(3) and 1.315(3) Å in **3** compared to both 1.3364(19) Å in **1**). The attached phosphane moiety is displaced, as observable in the *N*‐Si‐C16 bond angle (100.02(6) and 99.99(7)° in **1** and 140.05(11) and 97.41(10)° in **3**). Interestingly, in complexes **4**, **5** and **6**, both the phosphane and silylene moieties are efficiently coordinating the host atom **4**. This is the only complex featuring a transition metal coordinated to two ligands without any additional halide coordination. The copper center has a highly distorted tetrahedral environment (from 88 to 135°). In contrast to all other complexes **5**–**9**, the Si−N bond lengths (1.838(5) to 1.867(4) Å) are not significantly shortened compared to the bonds in **1** (mean 1.86 Å). For both Si atoms there is one shorter bond (mean 1.84 Å compared to mean 1.86 Å) coming along with a larger Cu‐Si‐N angle (mean 128° compared to 139°). Also the N‐Si‐C (mean 102°) and the N‐Si‐N (mean 70°) bond angles in **4** are closest to the values in **1** (100° and 69°, respectively), while **5**–**9** have larger values (104.60(13) in **8** to 117.77(9)° in **6**, and 71.43(12) in **8** to 73.09(8)° in **6**). Only in **6** the X−P distance is shorter than the X−Si bond. It is also the structure with the largest distance of X from the SiPCC plane although the B atom has smallest atomic radius. In the complexes **7**–**9**, no bonding with the phosphorus atom is observed, but the phosphane moiety is oriented towards the host atom, as a hint of potential weak interaction. The two aluminium complexes **7** and **8** do not exhibit noticeable differences, although the distance between the non‐bonding Al and P decreases about 0.23 Å (3.5920(7) Å, 3.3587(14) Å). A similar value of 3.5640(6) Å in **9** (isomorphous to **7**) between Ga and P is observable (Figure 9).

## Conclusions

In summary, we have synthesized a new hybrid bidentate phosphane‐silylene ligand and studied its coordination behavior with transition metal and main group precursors. Reaction of **1** with Se and GeCl_2_ resulted in selective oxidation of silylene moiety to form complexes **2** and **3**, respectively. The strong σ‐donating ligand **1** on reaction with CuCl and PhBCl_2_ results in dissociation leading to the formation of compounds **4** and **6**, respectively where the ligand displays a chelating mode of coordination. In complex **5**, the phosphane moiety also participates in bonding.

The ligand **1** then offers a different coordination environment for the LAlCl_3_ (**7**), LEtAlCl_2_ (**8**) and LGaCl_3_ (**9**) complexes: while the host atom is bonded to the silicon, the phosphane moiety is obviously preferably oriented towards it as well, which indicates that the two atoms are interacting together. Thus, the overall reactivity of the newly developed ligand (**1**) confirms that silylene is a strong σ‐donating ligand compared to phosphane. All compounds were fully characterized by single‐crystal X‐ray studies and various spectroscopic studies.

## Experimental Section

All manipulations were carried out under a dry argon or nitrogen atmosphere using Schlenk line and glovebox techniques. Tetrahydrofuran, toluene and hexane were dried by refluxing with sodium/potassium under N_2_ prior to use. The NMR (^1^H, ^13^C, ^31^P and ^29^Si) spectra were recorded on Bruker Avance II 500 MHz spectrometer. Melting points of compounds were measured in a sealed glass tube using the Büchi‐540 instrument. Elemental analyses were performed by the Analytisches Labor des Instituts für Anorganische Chemie der Universität Göttingen. Commercial reagents were purchased from Aldrich, Acros, or Alfa‐Aesar Chemical Co. and used as received. Heteroleptic chlorosilylene LSiCl (L=PhC(N*t*Bu)_2_) was prepared according to the literature.^[23]^

**Synthesis of [PhC(N*t*Bu)_2_SiC_6_H_4_PPh_2_] (1**): LSiCl (0.295 g, 1.0 mmol) and Ph_2_PC_6_H_4_Li (0.268 g, 1.0 mmol) were placed in a 100 mL round bottom flask. Toluene (40 mL) was added under stirring at −78 °C and the reaction mixture was stirred for 1 day. After filtration, the filtrate was concentrated to 5 mL under vacuum and stored at −30 °C in a freezer. After 24 h, purple crystals were obtained and collected by filtration (0.436 g, 84 %). Mp: 185 °C. ^1^H NMR (C_6_D_6_, 298 K): *δ*=1.11 (s, 18 H, *t*Bu), 7.00–7.02 (m, 1 H, Ph), 7.07–7.10 (m, 3 H, Ph), 7.19–7.27 (m, 8 H, Ph), 7.42–7.49 (m, 2 H, Ph), 7.70–7.75 (m, 4 H, Ph), 7.89–7.91 ppm (br, 1 H, Ph); ^13^C{^1^H} NMR (CD_2_Cl_2_, 298 K): *δ*=31.18, 56.70, 128.01, 128.35, 129.74, 129.84, 132.44, 132.53, 133.38 ppm; ^31^P{^1^H} NMR (202 MHz, 298 K, C_6_D_6_): *δ*=−11.2 ppm; ^29^Si{^1^H} NMR (99 MHz, 298 K, C_6_D_6_): *δ*=18.52 ppm (^1^ *J*_SiP_=410 Hz); elemental analysis calcd (%) for C_33_H_37_N_2_PSi: (MW=520.24): C 76.12, H 7.16, N 5.38; found: C 76.22, H 7.29, N 5.62.

**Synthesis of [(PhC(N*t*Bu)_2_C_6_H_4_PPh_2_)Se] (2)**: Ligand **1** (0.520 g, 1.0 mmol) and Se (0.79 g, 1.0 mmol) were placed in a 100 mL round bottom flask. Toluene (30 mL) was added under stirring at room temperature. The reaction mixture was stirred for 1 day. After solvent evaporation the white precipitate was washed with hexane two times and dissolved in distilled THF and concentrated to 5 mL under vacuum and stored at −30 °C in a freezer. After 4 weeks, colorless crystals were obtained and collected by filtration, (0.49 g, 82 %). Mp: 230 °C; ^1^H NMR (CD_2_Cl_2_, 298 K): *δ*=1.07 (s, 18 H, *t*Bu), 7.13–7.15 (m, 1 H, Ph), 7.22–7.25 (m, 4 H, Ph), 7.33–7.34 (m, 6 H, Ph), 7.38–7.40 (m, 1 H, Ph), 7.50–7.57 (m, 6 H, Ph), 7.90–7.92 ppm (m, 1 H, Ph), ^13^C{^1^H} NMR (CD_2_Cl_2_, 298 K): *δ*=26.12, 31.29, 56.18, 68.29, 127.62, 128.56, 128.73, 128.90, 129.12, 129.17, 131.30, 131.34, 133.50, 133.65, 137.13, 137.14, 138.83, 138.93, 141.33, 141.65, 176.30, 176.31 ppm; ^31^P{^1^H} NMR (202 MHz, 298 K, CD_2_Cl_2_) *δ*=−12.3 ppm; ^29^Si{^1^H} NMR (99 MHz, 298 K, CD_2_Cl_2_) *δ*=12.67 ppm (^1^ *J*_SiP_=25 Hz); elemental analysis calcd (%) for C_33_H_38_N_2_PSeSi (MW=600.16): C 65.98, H 6.38, N 4.66; found: C 66.23, H 6.54, N 4.83.

**Synthesis of [(PhC(N*t*Bu)_2_C_6_H_4_PPh_2_)Cl_2_] (3)**: Ligand **1** (0.260 g, 0.5 mmol) and GeCl_2_.dioxane (0.072 g, 0.5 mmol) were placed in a 100 mL round bottom flask. Diethyl ether (30 mL) was added under stirring at −78 °C. The reaction mixture was stirred overnight. After solvent evaporation the orange precipitate was washed with hexane two times and dissolved in distilled methylene chloride and concentrated to 5 mL under vacuum and stored at −30 °C in a freezer. After one week, yellow colored crystals were obtained and collected by filtration, (0.18 g, 62 %). Mp: 205 °C.; ^1^H NMR (C_6_D_6_, 298 K): *δ*=1.15 (s, 18 H, *t*Bu), 6.96–6.98 (m, 1 H, Ph), 7.00–7.06 (m, br, 9 H, Ph), 7.11–7.16 (m, 4 H, Ph), 7.31–7.36 (m, 4 H, Ph), 7.82–7.85 ppm (m, 1 H, Ph); ^13^C{^1^H} NMR (C_6_D_6_, 298 K): *δ*=21.38, 31.14, 55.77, 125.64, 127.48, 128.51, 128.53, 128.85, 128.90, 129.27, 130.41, 130.91, 131,60, 133.30, 133.44, 136.66, 136.67, 137.83, 139.07, 139.17, 142.32, 142.64, 175.27, 175.29 ppm; ^31^P{^1^H} NMR (202 MHz, 298 K, C_6_D_6_) *δ*=12.2 ppm; ^29^Si{1H} NMR (99 MHz, 298 K, CD_2_Cl_2_) *δ*=−25.06 ppm (^1^ *J*_SiP_=370 Hz); elemental analysis calcd (%) for C_33_H_38_Cl_2_N_2_PSi (MW=591.19): C 66.88, H 6.46, N 4.73; found: C 67.14, H 6.58, N 4.85.

**Synthesis of [(PhC(N*t*Bu)_2_C_6_H_4_PPh_2_)_2_Cu] (4)**: Ligand **1** (0.52 g, 1.0 mmol) and CuCl (0.99 g, 1.0 mmol) were placed in a 100 mL round bottom flask. Toluene (30 mL) was added under stirring at room temperature. The reaction mixture was stirred for 1 day. After filtration, the filtrate was concentrated to 5 mL under vacuum and stored at −30 °C in a freezer. After one week, yellow colored crystals were obtained and collected by filtration (0.49 g, 40 %). Mp: 280 °C.; ^1^H NMR (C_7_D_8_, 298 K): *δ*=1.05 (s, 18 H, *t*Bu), 1.18 (s, 18 H, *t*Bu), 6.73–6.78 (m, s, 4 H, Ph), 6.85–7.09 (m, 22 H, Ph), 7.20–7.22 (m, 2 H, Ph), 7.34–7.39 (m, 2 H, Ph), 7.51–7.55 (m, 2 H, Ph), 7.98–8.10 ppm (m, 6 H, Ph); ^13^C{^1^H} NMR (C_6_D_6_, 298 K): *δ*=15.55, 21.34, 29.14, 31.00, 31.86, 32.92, 54.42, 56.05, 65.88, 125.61, 128.43, 128.46, 129.27, 130.14, 133.24, 134.77, 134.88, 140.91, 152.92, 166.07 ppm; ^31^P{^1^H} NMR (202 MHz, 298 K, C_7_D_8_) *δ*=−8.7 ppm; elemental analysis calcd (%) for C_66_H_76_Cl_2_Cu_2_N_2_P_2_Si_2_ (MW=1238.30): C 63.85, H 6.17, N 4.51; found: C 63.98, H 6.44, N 4.64.

**Synthesis of [(PhC(N*t*Bu)_2_C_6_H_4_PPh_2_)ZnCl_2_] (5)**: Ligand **1** (0.52 g, 1.0 mmol) and ZnCl_2_ (0.99 g, 1.0 mmol) were placed in a 100 mL round bottom flask. Toluene (30 mL) was added under stirring at room temperature. The reaction mixture was stirred overnight. After solvent evaporation the white precipitate was washed with hexane two times and dissolved in distilled THF and concentrated to 5 mL under vacuum and stored at −30 °C in a freezer. After two weeks, colorless crystals were obtained and collected by filtration, (0.39 g, 60 %). Mp: 270 °C.; ^1^H NMR (CD_2_Cl_2_, 298 K): *δ*=1.19 (s, 18 H, *t*Bu), 7.05–7.28 (m, 3 H, Ph), 7.38–7.45 (m, 2 H, Ph), 7.52–7.62 (m, 10 H, Ph), 7.78–7.82 (m, 1 H, Ph), 8.95 ppm (br s, 3 H, Ph); ^13^C{^1^H} NMR (CD_2_Cl_2_, 298 K): *δ*=21.69, 31.28, 55.39, 125.78, 128.70, 129.22, 129.29, 129.51, 130.84, 130.85, 131.86, 134.71, 134.82, 136.50 ppm; ^31^P{^1^H} NMR (202 MHz, 298 K, CD_2_Cl_2_) *δ*=−26.7 ppm; ^29^Si{^1^H} NMR (99 MHz, 298 K, CD_2_Cl_2_) *δ*=31.57 ppm (^1^ *J*_SiP_=840 Hz); elemental analysis calcd (%) for C_33_H_37_Cl_2_N_2_PSiZn (MW=654.11): C 60.33, H 5.68, N 4.26; found: C 60.49, H 5.71, N 4.54.

**Synthesis of [(PhC(N*t*Bu)_2_C_6_H_4_PPh_2_)BPhCl] (6)**: Ligand **1** (0.52 g, 1.0 mmol) was placed in a 100 mL round bottom flask and dissolved in 50 mL toluene. PhBCl_2_ (0.130 mL, 1 mmol) was added under stirring at −78 °C. The reaction mixture was stirred overnight. After filtration, the filtrate was concentrated to 5 mL under vacuum and stored at room temperature. After two weeks, colorless crystals were obtained and collected by filtration, (0.58 g, 70 %), Mp: 185 °C; ^1^H NMR (CD_2_Cl_2_, 298 K): *δ*=1.00 (s, 18 H, *t*Bu), 7.12–7.16 (m, 3 H, Ph), 7.15–7.18 (m, 4 H, Ph), 7.22–7.25 (m, 5 H, Ph), 7.39–7.41 (m, 4 H, Ph), 7.42–7.45 (m, 3 H, Ph), 7.53–7.55 (m, 5 H, Ph), 7.56–7.60 ppm (m, 5 H, Ph); ^13^C{^1^H} NMR (CD_2_Cl_2_, 298 K): *δ*=29.30, 30.96, 31.25, 31.46, 31.90, 57.81, 126.84, 127.27, 128.54, 129.02, 129.17, 129.61, 129.79, 130.12, 133.05, 133.68, 136.10 ppm; ^31^P{^1^H} NMR (202 MHz, 298 K, CD_2_Cl_2_) *δ*=3.6 ppm; ^11^B{^1^H} NMR (CD_2_Cl_2_, 298 K): *δ*=10.44 ppm; elemental analysis calcd (%) for C_45_H_48_B_2_Cl_4_N_2_PSi (MW=837.22): C 64.39, H 5.76, N 3.34; found: C 64.71, H 5.84, N 3.55.

**Synthesis of [(PhC(N*t*Bu)_2_C_6_H_4_PPh_2_)AlCl_3_] (7)**: Ligand **1** (0.26 g, 0.5 mmol) and AlCl_3_ (0.067 g, 0.5 mmol) were placed in a 100 mL round bottom flask. Diethyl ether (30 mL) was added under stirring at −78 °C. The reaction mixture was stirred overnight. After solvent evaporation, the white precipitate was washed twice with hexane, dissolved in distilled methylene chloride and concentrated to 5 mL under vacuum and stored at 0 °C in a freezer. After one week, colorless needle shape crystals were obtained and collected by filtration, (0.25 g, 78 %); Mp: 240 °C.; ^1^H NMR (CD_2_Cl_2_, 298 K): *δ*=1.10 (s, 18 H, *t*Bu), 7.36 (s, 9 H, Ph), 7.44–7.49 (m, 1 H, Ph), 7.58–7.66 (m, 7 H, Ph), 7.75–7.85 ppm (m, 2 H, Ph); ^13^C{^1^H} NMR (CD_2_Cl_2_, 298 K): *δ*=14.52, 30.83, 31.19, 55.77, 128.27, 128.62, 131.37, 132.42, 133.33 ppm; ^31^P{^1^H} NMR (202 MHz, 298 K, CD_2_Cl_2_) *δ*=−17.3 ppm; elemental analysis calcd (%) for C_33_H_38_AlCl_3_N_2_PSi (MW=653.14): C 60.51, H 5.85, N 4.28; found: C 60.73, H 5.89, N 4.42.

**Synthesis of [(PhC(N*t*Bu)_2_C_6_H_4_PPh_2_)AlEtCl_2_] (8)**: Ligand **1** (0.52 g, 1.0 mmol) was placed in a 100 mL round bottom flask and dissolved in 30 mL diethyl ether. EtAlCl_2_ (0.50 mL, 1 m) was added under stirring at −78 °C. The reaction mixture was stirred overnight. After filtration, the filtrate was concentrated to 5 mL under vacuum and stored at −30 °C in a freezer. After one week, colorless crystals were obtained and collected by filtration, (0.44 g, 68 %); Mp: 120 °C.; ^1^H NMR (C_6_D_6_, 298 K): *δ*=1.03 (s, 18 H, *t*Bu), 1.77 (t, *J*=15 Hz, 3 H, Me), 6.88–7.07 (m, 12 H, Ph), 7.23 (br s, 2 H, Ph), 7.53 (br s, 3 H, Ph), 7.78–7.97 ppm (m, 2 H, Ph); ^13^C{^1^H} NMR (C_6_D_6_, 298 K): *δ*=15.54, 31.10, 65.88, 127.91, 128.10, 128.29, 128.42, 128.54, 128.77, 130.77, 131.11 ppm; ^31^P{^1^H} NMR (202 MHz, 298 K, C_6_D_6_) *δ*=−16.4 ppm; elemental analysis calcd (%) for C_35_H_43_AlCl_2_N_2_PSi (MW=647.21): C 64.81, H 6.68, N 4.32; found: C 65.08, H 6.77, N 4.49.

**Synthesis of [(PhC(N*t*Bu)_2_C_6_H_4_PPh_2_)GaCl_3_] (9)**: Ligand **1** (0.26 g, 0.5 mmol) and GaCl_3_ (0.088 g, 0.5 mmol) were placed in a 100 mL round bottom flask. Diethyl ether (30 mL) was added under stirring at −78 °C. The reaction mixture was stirred overnight. After solvent evaporation the white precipitate was washed with hexane two times and dissolved in distilled methylene chloride and concentrated to 5 mL under vacuum and stored at 0 °C in a freezer. After one week, colorless crystals were obtained and collected by filtration, (0.29 g, 85 %); Mp: 220 °C.;^1^H NMR (CD_2_Cl_2_, 298 K): *δ*=1.09 (s, 18 H, *t*Bu), 7.24 (br s, 3 H, Ph), 7.36 (s, 8 H, Ph), 7.58–7.64 ppm (m, 8 H, Ph); ^13^C{^1^H} NMR (CD_2_Cl_2_, 298 K): *δ*=15.66, 31.32, 31.56, 31.67, 66.21, 129.47, 129.52, 129.58, 129.68, 130.88, 132.30, 133.49, 134.01 ppm; ^31^P{^1^H} NMR (202 MHz, 298 K, CD_2_Cl_2_) *δ*=−13.3 ppm; ^29^Si{^1^H} NMR (99 MHz, 298 K, CD_2_Cl_2_): *δ*=11.25 ppm (^1^ *J*_SiP_=880 Hz); ^71^Ga{^1^H} NMR (CD_2_Cl_2_, 298 K, ppm) *δ*=250.56 ppm; elemental analysis calcd (%) for C_33_H_38_GaCl_3_N_2_PSi (MW=695.08): C 58.80, H 5.49, N 4.01; found: C 58.94, H 5.79, N 4.32.

**Crystallographic details**: The datasets were collected on an Incoatec Mo/Ag Microsource^[26]^ (**1, 3, 7**–**8**) and on a Bruker TXS‐Mo rotating anode (**2, 4**–**6**) with mirror optics and an APEX II detector with a D8 goniometer. The data were integrated with SAINT.^[27]^ A multiscan absorption correction and a 3λ^[28]^ correction (**1**–**3**, **5**–**9**) was applied using SADABS.^[29]^ For **4** the multi‐scan absorption correction was applied using TWINABS.^[30]^ The structures were solved by SHELXT^[31]^ and refined on F^2^ using SHELXL^[32]^ in the graphical user interface ShelXle.^[33]^ Full crystallographic data is available in the Supporting Information. Deposition numbers 1994257 (**1**); 1994258 (**2**), 1994259 (**3**), 1994260 (**4**), 1994261 (**5**), 1994262 (**6**) 1994263 (**7**) 1994264 (**8**) and 1994265 (**9**) contain the supplementary crystallographic data for this paper. These data are provided free of charge by the joint Cambridge Crystallographic Data Centre and Fachinformationszentrum Karlsruhe Access Structures service www.ccdc.cam.ac.uk/structures.

## Conflict of interest

The authors declare no conflict of interests.

## Supporting information

As a service to our authors and readers, this journal provides supporting information supplied by the authors. Such materials are peer reviewed and may be re‐organized for online delivery, but are not copy‐edited or typeset. Technical support issues arising from supporting information (other than missing files) should be addressed to the authors.

Supplementary
