# Supplementary material for: Synthesis and Coordination Behavior of a New Hybrid Bidentate Ligand with Phosphine and Silylene Donors
Source: Chemistry. 2020 Dec 14;27(5):1744–52. doi: 10.1002/chem.202003513 (PMC7898821; doi:10.1002/chem.202003513)
Supplement: Supplementary file 1 — Supplementary [file CHEM-27-1744-s001.pdf]

Chemistry–A European Journal

Supporting Information

Synthesis and Coordination Behavior of a New Hybrid Bidentate Ligand with Phosphine and Silylene Donors

Mohd Nazish, Mujahuddin M. Siddiqui, Samir Kumar Sarkar, Annika Münch, Christina M. Legendre, Regine Herbst-Irmer, Dietmar Stalke,* and Herbert W. Roesky*[a]

Table of Contents

S1. X-ray crystallographic analysis 2

S1.1. Crystal structure of **1** 5

S1.2. Crystal structure of **2** 7

S1.3. Crystal structure of **3** 10

S1.4. Crystal structure of **4** 13

S1.5. Crystal structure of **5** 20

S1.6. Crystal structure of **6** 23

S1.7. Crystal structure of **7** 26

S1.8. Crystal structure of **8** 29

S1.9. Crystal structure of **9** 32

S2 NMR spectra 35

S3 Mass Spectra 535

S4 Comparison graphs of Copper complexes 57

References 3559

S1. X-ray crystallographic analysis

The datasets were collected on an Incoatec Mo/Ag Microsource[S1] (**1,3,7-8**) and on a Bruker TXS-Mo rotating anode (**2,4-6**) with mirror optics and an APEX II detector with a D8 goniometer. The data were integrated with SAINT.[S2] A multi-scan absorption correction 3λ[S3] correction was applied using SADABS[S4] (**1**-**3**,**5**-**9**). A multi-scan absorption correction was applied using TWINABS (**4**) [S5]. The structures were solved by SHELXT[S6] and refined on F^2 using SHELXL[S7] in the graphical user interface ShelXle.[S8] For the refinement of disordered moieties, distance restraints and restraints for the anisotropic displacement parameters were used.[S9]

Table S1 Crystal data at 100 K.

Compound	**1**	**2**	**3**	**4**	**5**	**6**	**7**	**8**	**9**
Formula	$C_{33}H_{37}N_2PSi$	$C_{39}H_{49}N_2O_{1.5}PSeSi$	$C_{33}H_{37}Cl_2N_2PSi$	$C_{97.5}H_{110}Cl_2Cu_2N_4P_2Si_2$	$C_{33}H_{37}Cl_2N_2PSiZn$	$C_{45}H_{47}B_2Cl_4N_2PSi$	$C_{33.5}H_{38}AlCl_4N_2PSi$	$C_{35}H_{42}AlCl_2N_2PSi$	$C_{33.5}H_{38}Cl_4N_2PSiGa$
Mol. w., g mol^{-1}	520.70	707.82	591.60	1653.99	656.97	838.32	696.50	647.64	739.24
CCDC no.	1994257	1994258	1994259	1994260	1994261	1994262	1994263	1994264	1994265
Wavelength, Å	0.56086	0.71073	0.71073	0.71073	0.71073	0.71073	0.71073	0.71073	0.71073
Crystal system	Monoclinic	Monoclinic	Monoclinic	Triclinic	Monoclinic	Triclinic	Monoclinic	Orthorhombic	Monoclinic
Space group	$C2/c$	$C2/c$	$P2_1/n$	$P\bar{1}$	$P2_1/c$	$P\bar{1}$	$P2_1/c$	$P2_12_12_1$	$P2_1/c$
a, *b*, Å	15.896(2)	36.923(8)	8.783(2)	14.480(2)	17.348(3)	9.207(2)	9.089(2)	8.822(2)	9.034(2)
b, Å	12.895(2)	10.143(2)	19.561(3)	22.792(2)	10.356(2)	13.994(2)	17.068(2)	16.509(2)	17.084(2)
c, Å	29.689(3)	20.797(4)	17.975(3)	28.789(3)	19.683(7)	17.954(3)	22.497(3)	23.720(3)	22.542(3)
α				99.83(2)		108.67(3)			
β	97.67(3)	110.56(2)	93.51(3)	102.72(3)	113.70(2)	97.55(2)	95.11(2)		95.28(2)
γ				99.98(2)		95.43(2)			
V, Å^3	6031.2(14)	7293(3)	3082.4(10)	8909(2)	3237.9(15)	2149.4(8)	3476.1(10)	3454.6(10)	3464.3(10)

Z	8	8	4	4	4	2	4	4	4
Density Mg/m^3	1.147	1.289	1.275	1.233	1.348	1.295	1.331	1.245	1.417
Absorption coefficient, mm^{-1}	0.087	1.141	0.327	0.648	1.035	0.375	0.473	0.321	1.210
Crystal size, mm	0.635 x 0.404 x 0.292	0.384 x 0.383 x 0.287	0.243 x 0.170 x 0.143	0.289 x 0.198 x 0.130	0.302 x 0.128 x 0.042	0.334 x 0.184 x 0.164	0.281 x 0.223 x 0.113	0.190 x 0.146 x 0.125	0.528 x 0.463 x 0.332
Theta range,°	1.61 to 20.30	1.18 to 30.54	1.54 to 25.39	1.47 to 25.12	2.26 to 26.82	2.26 to 26.53	1.82 to 26.42	1.50 to 25.39	1.50 to 26.39
Refl. measured	94961	121654	50154	108376	57492	44441	76991	31197	57981
Refl. unique	5974	10844	5663	30666	6795	8889	7136	6360	7096
R_{int}	0.0413	0.0270	0.0968	0.0829	0.0746	0.0496	0.0352	0.0532	0.0331
Data/ restr./para.	5974 / 0 / 340	10844 / 544 / 532	5663 / 0 / 358	31322 / 7081 / 2197	6795 / 0 / 367	8889 / 72 / 502	7136 / 377 / 419	6360 / 89 / 413	7096 / 20 / 403
R_1 [I>2σ(I)]	0.0394	0.0252	0.0462	0.0615	0.0337	0.0399	0.0279	0.0352	0.0229
wR_2 (all refl.)	0.0932	0.0677	0.1174	0.1580	0.0791	0.1097	0.0722	0.0807	0.0579
Absolute structure parameter [S10]	-	-	-	-	-	-	-	0.05(3)	-
Extinction coefficient	-	0.00069(4)	-	-	-	-	-	-	-
$\Delta\rho_{fin}$, e Å^{-3}	0.354 / -0.303	0.452 / -0.402	0.549 / -0.370	1.308 / -0.424	0.403 /-0.395	0.704 / -0.394	0.343 / -0.275	0.277 / -0.263	0.391 /-0.295

S1.1. Crystal structure of **1**

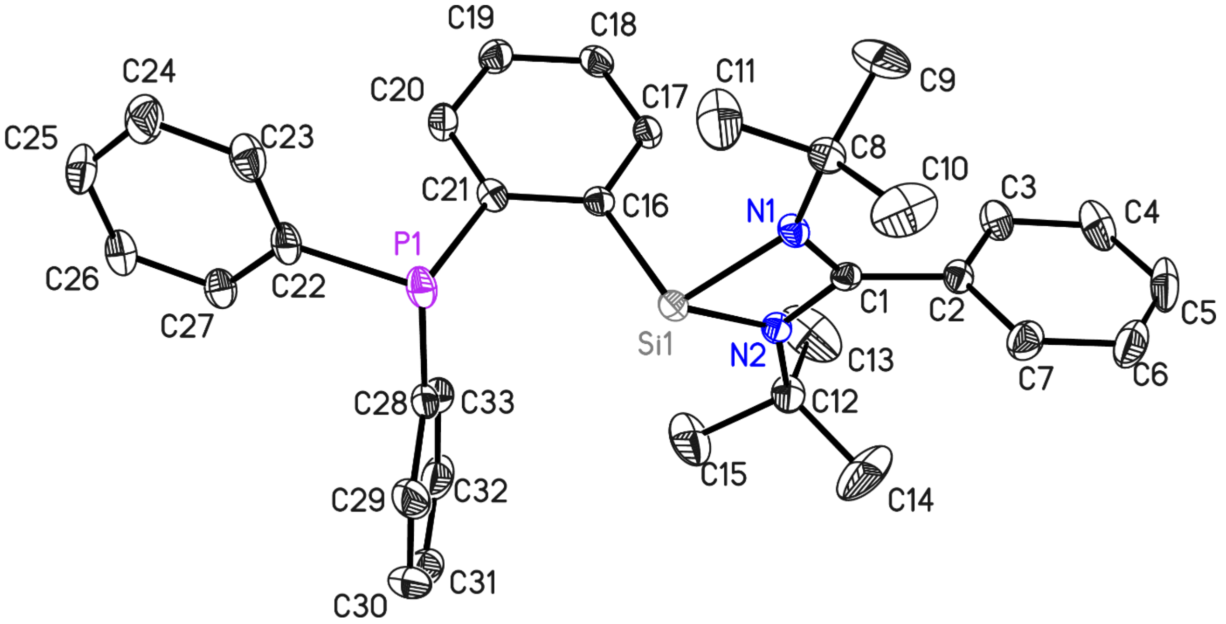

Figure 1 Molecular structure of **1** with thermal ellipsoids at 50% probability level. The hydrogen atoms are omitted for clarity.

Table 2. Bond lengths [Å] and angles [°] for **1**.

P(1)-C(28)	1.8283(18)
P(1)-C(22)	1.8384(17)
P(1)-C(21)	1.8452(16)
Si(1)-N(1)	1.8595(13)
Si(1)-N(2)	1.8696(13)
Si(1)-C(16)	1.9315(15)
Si(1)-C(1)	2.3370(16)
N(1)-C(1)	1.3364(19)
N(1)-C(8)	1.4695(19)
C(1)-N(2)	1.3346(19)
C(1)-C(2)	1.486(2)
N(2)-C(12)	1.4738(19)
C(2)-C(3)	1.388(2)
C(2)-C(7)	1.391(2)
C(3)-C(4)	1.391(2)
C(4)-C(5)	1.381(3)
C(5)-C(6)	1.379(3)
C(6)-C(7)	1.381(2)
C(8)-C(9)	1.515(2)
C(8)-C(10)	1.520(2)
C(8)-C(11)	1.523(2)
C(12)-C(14)	1.510(3)
C(12)-C(15)	1.515(2)
C(12)-C(13)	1.527(3)
C(16)-C(17)	1.398(2)
C(16)-C(21)	1.410(2)
C(17)-C(18)	1.386(2)
C(18)-C(19)	1.385(2)
C(19)-C(20)	1.383(2)
C(20)-C(21)	1.397(2)
C(22)-C(27)	1.388(2)
C(22)-C(23)	1.397(2)
C(23)-C(24)	1.382(3)
C(24)-C(25)	1.386(3)
C(25)-C(26)	1.382(3)
C(26)-C(27)	1.387(2)
C(28)-C(33)	1.391(3)
C(28)-C(29)	1.395(2)
C(29)-C(30)	1.389(3)
C(30)-C(31)	1.377(4)
C(31)-C(32)	1.373(3)
C(32)-C(33)	1.389(2)

C(28)-P(1)-C(22)	101.20(8)	C(10)-C(8)-C(11)	109.39(17)
C(28)-P(1)-C(21)	102.31(7)	N(2)-C(12)-C(14)	112.45(15)
C(22)-P(1)-C(21)	101.37(7)	N(2)-C(12)-C(15)	105.64(13)
N(1)-Si(1)-N(2)	69.28(6)	C(14)-C(12)-C(15)	109.87(18)
N(1)-Si(1)-C(16)	100.02(6)	N(2)-C(12)-C(13)	109.11(15)
N(2)-Si(1)-C(16)	99.99(7)	C(14)-C(12)-C(13)	111.7(2)
N(1)-Si(1)-C(1)	34.84(5)	C(15)-C(12)-C(13)	107.80(17)
N(2)-Si(1)-C(1)	34.80(5)	C(17)-C(16)-C(21)	117.44(14)
C(16)-Si(1)-C(1)	105.97(6)	C(17)-C(16)-Si(1)	122.25(11)
C(1)-N(1)-C(8)	132.29(13)	C(21)-C(16)-Si(1)	120.31(11)
C(1)-N(1)-Si(1)	92.51(9)	C(18)-C(17)-C(16)	122.08(14)
C(8)-N(1)-Si(1)	134.66(10)	C(19)-C(18)-C(17)	119.73(15)
N(2)-C(1)-N(1)	105.05(13)	C(20)-C(19)-C(18)	119.69(15)
N(2)-C(1)-C(2)	126.90(13)	C(19)-C(20)-C(21)	120.82(15)
N(1)-C(1)-C(2)	127.81(13)	C(20)-C(21)-C(16)	120.23(14)
N(2)-C(1)-Si(1)	53.08(8)	C(20)-C(21)-P(1)	121.94(12)
N(1)-C(1)-Si(1)	52.65(8)	C(16)-C(21)-P(1)	117.77(11)
C(2)-C(1)-Si(1)	168.64(11)	C(27)-C(22)-C(23)	118.34(15)
C(1)-N(2)-C(12)	130.84(13)	C(27)-C(22)-P(1)	125.02(13)
C(1)-N(2)-Si(1)	92.12(9)	C(23)-C(22)-P(1)	116.59(13)
C(12)-N(2)-Si(1)	135.07(11)	C(24)-C(23)-C(22)	120.90(17)
C(3)-C(2)-C(7)	119.73(15)	C(23)-C(24)-C(25)	119.99(17)
C(3)-C(2)-C(1)	120.76(14)	C(26)-C(25)-C(24)	119.79(17)
C(7)-C(2)-C(1)	119.44(14)	C(25)-C(26)-C(27)	120.09(17)
C(2)-C(3)-C(4)	119.72(16)	C(26)-C(27)-C(22)	120.87(16)
C(5)-C(4)-C(3)	119.95(18)	C(33)-C(28)-C(29)	118.32(18)
C(6)-C(5)-C(4)	120.45(16)	C(33)-C(28)-P(1)	123.65(13)
C(5)-C(6)-C(7)	119.89(17)	C(29)-C(28)-P(1)	118.02(15)
C(6)-C(7)-C(2)	120.25(17)	C(30)-C(29)-C(28)	120.2(2)
N(1)-C(8)-C(9)	109.96(13)	C(31)-C(30)-C(29)	120.6(2)
N(1)-C(8)-C(10)	111.84(14)	C(32)-C(31)-C(30)	119.8(2)
C(9)-C(8)-C(10)	110.66(16)	C(31)-C(32)-C(33)	120.1(2)
N(1)-C(8)-C(11)	104.94(13)	C(32)-C(33)-C(28)	120.94(18)
C(9)-C(8)-C(11)	109.90(16)		

S1.2. Crystal structure of **2**

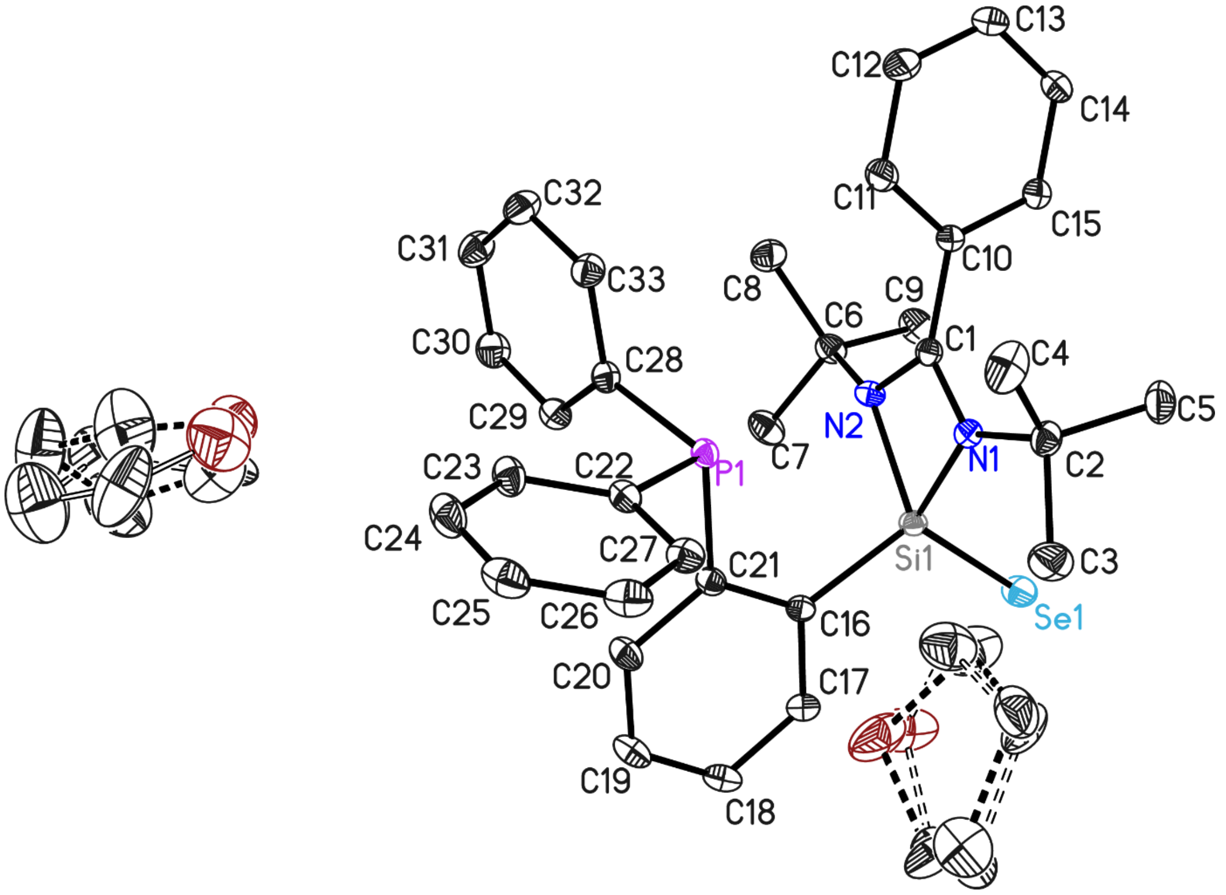

Figure 2 Molecular structure of **2** with thermal ellipsoids at 50% probability level. It consists of two fragments, one complex molecule and one and a half THF solvent molecules. The hydrogen atoms are omitted for clarity

The THF molecules are disordered in two and four positions, from which two were related by a crystallographic two-fold axis. Both were refined with distance restraints and restraints for the anisotropic displacement parameters. The occupancy of the major components refined to 0.500(17) and 0.347(7), respectively.

Table 3. Bond lengths [Å] and angles [°] for **2**.

		Si(1)-N(2)	1.8249(9)
Se(1)-Si(1)	2.1330(5)	Si(1)-C(16)	1.8847(10)
P(1)-C(28)	1.8248(10)	Si(1)-C(1)	2.2748(10)
P(1)-C(22)	1.8366(10)	N(1)-C(1)	1.3364(12)
P(1)-C(21)	1.8439(10)	N(1)-C(2)	1.4833(13)
Si(1)-N(1)	1.8224(9)	C(1)-N(2)	1.3466(12)

C(1)-C(10)	1.4834(13)	C(34A)-C(35A)	1.534(9)
N(2)-C(6)	1.4841(13)	C(35A)-C(36A)	1.529(8)
C(2)-C(4)	1.5252(15)	C(36A)-C(37A)	1.504(9)
C(2)-C(3)	1.5294(15)	O(2)-C(41)	1.385(8)
C(2)-C(5)	1.5300(15)	O(2)-C(38)	1.419(8)
C(6)-C(8)	1.5288(15)	C(38)-C(39)	1.505(9)
C(6)-C(7)	1.5306(14)	C(39)-C(40)	1.520(8)
C(6)-C(9)	1.5329(14)	C(40)-C(41)	1.521(8)
C(10)-C(11)	1.3910(14)	O(2A)-C(38A)	1.391(12)
C(10)-C(15)	1.3933(13)	O(2A)-C(41A)	1.406(11)
C(11)-C(12)	1.3886(15)	C(38A)-C(39A)	1.481(13)
C(12)-C(13)	1.3883(16)	C(39A)-C(40A)	1.544(14)
C(13)-C(14)	1.3850(16)	C(40A)-C(41A)	1.498(13)
C(14)-C(15)	1.3903(14)	C(28)-P(1)-C(22)	101.72(5)
C(16)-C(17)	1.4084(13)	C(28)-P(1)-C(21)	104.19(4)
C(16)-C(21)	1.4151(13)	C(22)-P(1)-C(21)	100.03(4)
C(17)-C(18)	1.3915(14)	N(1)-Si(1)-N(2)	72.04(4)
C(18)-C(19)	1.3879(15)	N(1)-Si(1)-C(16)	116.50(4)
C(19)-C(20)	1.3880(14)	N(2)-Si(1)-C(16)	111.61(4)
C(20)-C(21)	1.4035(13)	N(1)-Si(1)-Se(1)	116.19(3)
C(22)-C(23)	1.3979(14)	N(2)-Si(1)-Se(1)	117.52(3)
C(22)-C(27)	1.3986(14)	C(16)-Si(1)-Se(1)	115.75(3)
C(23)-C(24)	1.3952(15)	N(1)-Si(1)-C(1)	35.97(4)
C(24)-C(25)	1.3871(18)	N(2)-Si(1)-C(1)	36.30(4)
C(25)-C(26)	1.3886(16)	C(16)-Si(1)-C(1)	123.36(4)
C(26)-C(27)	1.3930(14)	Se(1)-Si(1)-C(1)	120.82(3)
C(28)-C(29)	1.3967(14)	C(1)-N(1)-C(2)	131.25(8)
C(28)-C(33)	1.4035(14)	C(1)-N(1)-Si(1)	90.80(6)
C(29)-C(30)	1.3933(15)	C(2)-N(1)-Si(1)	137.22(7)
C(30)-C(31)	1.3872(15)	N(1)-C(1)-N(2)	106.14(8)
C(31)-C(32)	1.3939(15)	N(1)-C(1)-C(10)	127.16(8)
C(32)-C(33)	1.3863(15)	N(2)-C(1)-C(10)	126.69(8)
O(1)-C(37)	1.437(8)	N(1)-C(1)-Si(1)	53.23(5)
O(1)-C(34)	1.476(9)	N(2)-C(1)-Si(1)	53.34(5)
C(34)-C(35)	1.514(9)	C(10)-C(1)-Si(1)	173.26(7)
C(35)-C(36)	1.520(9)	C(1)-N(2)-C(6)	130.53(8)
C(36)-C(37)	1.507(9)	C(1)-N(2)-Si(1)	90.36(6)
O(1A)-C(37A)	1.405(9)	C(6)-N(2)-Si(1)	134.22(7)
O(1A)-C(34A)	1.469(9)	N(1)-C(2)-C(4)	111.59(8)

N(1)-C(2)-C(3)	105.46(8)	C(25)-C(24)-C(23)	120.40(10)
C(4)-C(2)-C(3)	109.59(10)	C(24)-C(25)-C(26)	119.75(10)
N(1)-C(2)-C(5)	109.73(8)	C(25)-C(26)-C(27)	120.13(10)
C(4)-C(2)-C(5)	110.69(9)	C(26)-C(27)-C(22)	120.60(10)
C(3)-C(2)-C(5)	109.65(9)	C(29)-C(28)-C(33)	118.64(9)
N(2)-C(6)-C(8)	111.85(8)	C(29)-C(28)-P(1)	124.93(8)
N(2)-C(6)-C(7)	105.43(8)	C(33)-C(28)-P(1)	116.33(7)
C(8)-C(6)-C(7)	109.43(9)	C(30)-C(29)-C(28)	120.45(10)
N(2)-C(6)-C(9)	109.56(8)	C(31)-C(30)-C(29)	120.25(10)
C(8)-C(6)-C(9)	110.88(8)	C(30)-C(31)-C(32)	119.93(10)
C(7)-C(6)-C(9)	109.54(9)	C(33)-C(32)-C(31)	119.83(10)
C(11)-C(10)-C(15)	120.33(9)	C(32)-C(33)-C(28)	120.88(9)
C(11)-C(10)-C(1)	120.16(9)	C(37)-O(1)-C(34)	104.3(7)
C(15)-C(10)-C(1)	119.51(8)	O(1)-C(34)-C(35)	102.8(7)
C(12)-C(11)-C(10)	119.40(10)	C(34)-C(35)-C(36)	104.0(7)
C(13)-C(12)-C(11)	120.39(10)	C(37)-C(36)-C(35)	104.2(7)
C(14)-C(13)-C(12)	120.15(10)	O(1)-C(37)-C(36)	98.7(8)
C(13)-C(14)-C(15)	119.94(10)	C(37A)-O(1A)-C(34A)	107.6(6)
C(14)-C(15)-C(10)	119.79(9)	O(1A)-C(34A)-C(35A)	106.8(6)
C(17)-C(16)-C(21)	117.83(8)	C(36A)-C(35A)-C(34A)	104.8(6)
C(17)-C(16)-Si(1)	114.48(7)	C(37A)-C(36A)-C(35A)	101.2(6)
C(21)-C(16)-Si(1)	127.56(7)	O(1A)-C(37A)-C(36A)	111.7(7)
C(18)-C(17)-C(16)	122.12(9)	C(41)-O(2)-C(38)	106.0(6)
C(19)-C(18)-C(17)	119.40(9)	O(2)-C(38)-C(39)	103.8(6)
C(18)-C(19)-C(20)	119.75(9)	C(38)-C(39)-C(40)	99.4(7)
C(19)-C(20)-C(21)	121.49(9)	C(39)-C(40)-C(41)	104.2(5)
C(20)-C(21)-C(16)	119.30(9)	O(2)-C(41)-C(40)	106.9(5)
C(20)-C(21)-P(1)	119.33(7)	C(38A)-O(2A)-C(41A)	109.5(11)
C(16)-C(21)-P(1)	121.24(7)	O(2A)-C(38A)-C(39A)	108.3(11)
C(23)-C(22)-C(27)	118.84(9)	C(38A)-C(39A)-C(40A)	105.7(11)
C(23)-C(22)-P(1)	124.85(8)	C(41A)-C(40A)-C(39A)	99.8(9)
C(27)-C(22)-P(1)	116.31(7)	O(2A)-C(41A)-C(40A)	109.3(11)
C(24)-C(23)-C(22)	120.28(10)		

S1.3. Crystal structure of **3**

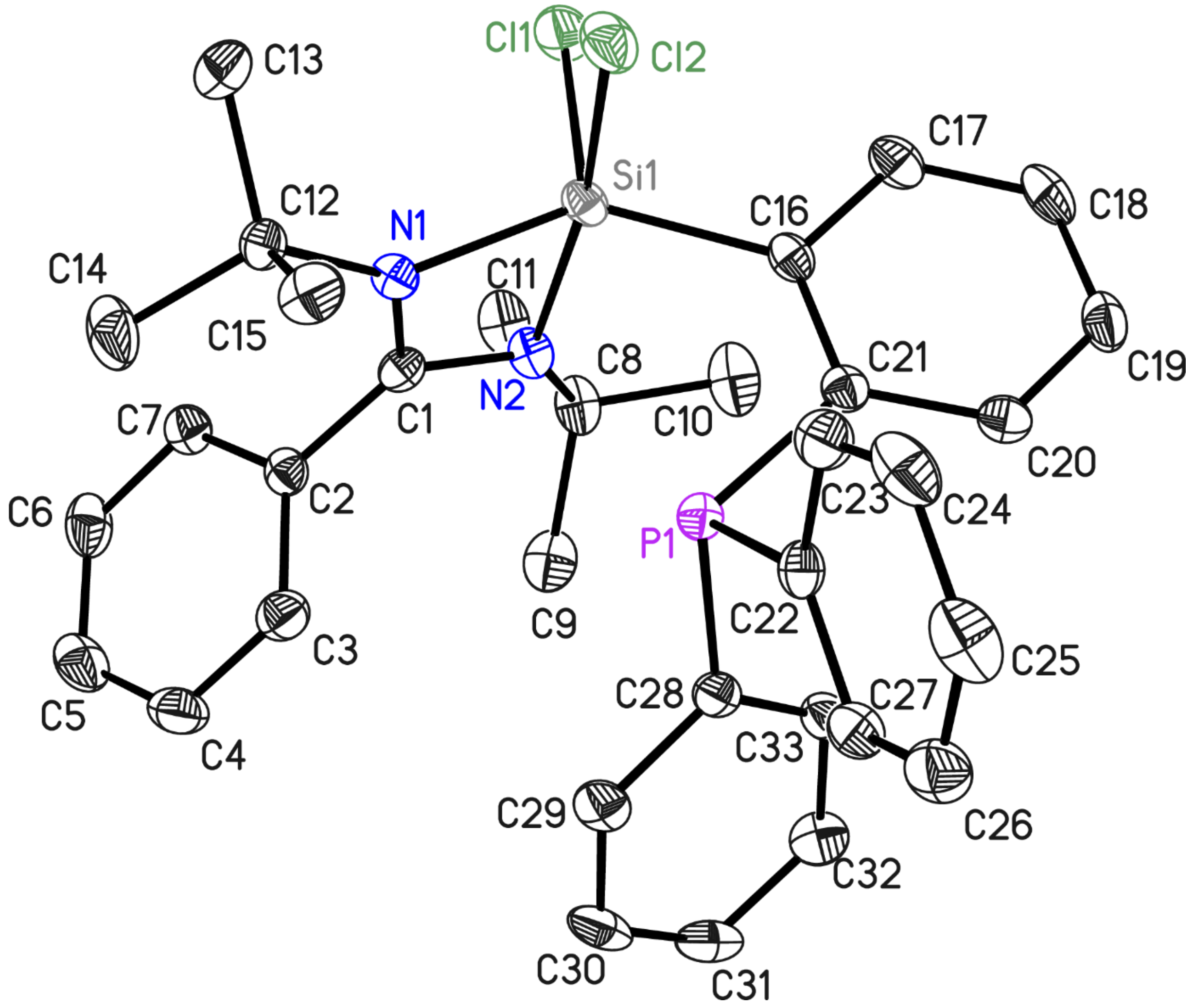

Figure 3 Molecular structure of **3** with thermal ellipsoids at 50% probability level. The hydrogen atoms are omitted for clarity.

Table 4. Bond lengths [Å] and angles [°] for **3**.

C(1)-N(2)	1.315(3)	Si(1)-Cl(2)	2.2113(11)
C(1)-N(1)	1.353(3)	P(1)-C(21)	1.828(3)
C(1)-C(2)	1.492(4)	P(1)-C(22)	1.837(3)
C(1)-Si(1)	2.341(3)	P(1)-C(28)	1.838(3)
Si(1)-N(1)	1.832(2)	N(1)-C(12)	1.507(3)
Si(1)-C(16)	1.896(3)	N(2)-C(8)	1.502(3)
Si(1)-N(2)	1.937(2)	C(2)-C(7)	1.387(4)
Si(1)-Cl(1)	2.0934(10)	C(2)-C(3)	1.395(4)
		C(3)-C(4)	1.383(4)
		C(4)-C(5)	1.377(4)

C(5)-C(6)	1.383(4)	N(1)-Si(1)-Cl(2)	97.68(7)
C(6)-C(7)	1.391(4)	C(16)-Si(1)-Cl(2)	89.39(8)
C(8)-C(10)	1.530(4)	N(2)-Si(1)-Cl(2)	166.14(7)
C(8)-C(11)	1.532(4)	Cl(1)-Si(1)-Cl(2)	94.55(4)
C(8)-C(9)	1.538(4)	N(1)-Si(1)-C(1)	35.25(9)
C(12)-C(15)	1.525(4)	C(16)-Si(1)-C(1)	123.13(10)
C(12)-C(13)	1.528(4)	N(2)-Si(1)-C(1)	34.18(9)
C(12)-C(14)	1.542(4)	Cl(1)-Si(1)-C(1)	102.97(7)
C(16)-C(21)	1.398(4)	Cl(2)-Si(1)-C(1)	132.81(7)
C(16)-C(17)	1.403(4)	C(21)-P(1)-C(22)	100.49(12)
C(17)-C(18)	1.383(4)	C(21)-P(1)-C(28)	104.50(12)
C(18)-C(19)	1.385(4)	C(22)-P(1)-C(28)	103.00(12)
C(19)-C(20)	1.380(4)	C(1)-N(1)-C(12)	130.5(2)
C(20)-C(21)	1.402(4)	C(1)-N(1)-Si(1)	93.39(16)
C(22)-C(27)	1.392(4)	C(12)-N(1)-Si(1)	136.00(17)
C(22)-C(23)	1.402(4)	C(1)-N(2)-C(8)	128.4(2)
C(23)-C(24)	1.374(4)	C(1)-N(2)-Si(1)	89.97(16)
C(24)-C(25)	1.383(4)	C(8)-N(2)-Si(1)	139.92(17)
C(25)-C(26)	1.382(4)	C(7)-C(2)-C(3)	120.0(3)
C(26)-C(27)	1.386(4)	C(7)-C(2)-C(1)	121.8(2)
C(28)-C(33)	1.398(4)	C(3)-C(2)-C(1)	118.2(2)
C(28)-C(29)	1.400(4)	C(4)-C(3)-C(2)	119.9(3)
C(29)-C(30)	1.384(4)	C(5)-C(4)-C(3)	120.2(3)
C(30)-C(31)	1.384(4)	C(4)-C(5)-C(6)	120.1(3)
C(31)-C(32)	1.383(4)	C(5)-C(6)-C(7)	120.5(3)
C(32)-C(33)	1.386(4)	C(2)-C(7)-C(6)	119.3(3)
		N(2)-C(8)-C(10)	107.8(2)
N(2)-C(1)-N(1)	107.2(2)	N(2)-C(8)-C(11)	109.1(2)
N(2)-C(1)-C(2)	126.3(2)	C(10)-C(8)-C(11)	108.7(2)
N(1)-C(1)-C(2)	126.4(2)	N(2)-C(8)-C(9)	112.2(2)
N(2)-C(1)-Si(1)	55.85(13)	C(10)-C(8)-C(9)	108.6(2)
N(1)-C(1)-Si(1)	51.36(13)	C(11)-C(8)-C(9)	110.3(2)
C(2)-C(1)-Si(1)	177.70(19)	N(1)-C(12)-C(15)	109.1(2)
N(1)-Si(1)-C(16)	140.05(11)	N(1)-C(12)-C(13)	107.9(2)
N(1)-Si(1)-N(2)	69.42(9)	C(15)-C(12)-C(13)	112.2(2)
C(16)-Si(1)-N(2)	97.41(10)	N(1)-C(12)-C(14)	114.0(2)
N(1)-Si(1)-Cl(1)	108.26(8)	C(15)-C(12)-C(14)	107.5(2)
C(16)-Si(1)-Cl(1)	110.29(9)	C(13)-C(12)-C(14)	106.2(2)
N(2)-Si(1)-Cl(1)	94.39(7)	C(21)-C(16)-C(17)	118.1(2)

C(21)-C(16)-Si(1)	123.52(19)	C(23)-C(24)-C(25)	120.4(3)
C(17)-C(16)-Si(1)	118.3(2)	C(26)-C(25)-C(24)	119.8(3)
C(18)-C(17)-C(16)	121.4(3)	C(25)-C(26)-C(27)	120.2(3)
C(17)-C(18)-C(19)	120.0(3)	C(26)-C(27)-C(22)	120.5(3)
C(20)-C(19)-C(18)	119.6(3)	C(33)-C(28)-C(29)	118.1(2)
C(19)-C(20)-C(21)	120.9(3)	C(33)-C(28)-P(1)	124.6(2)
C(16)-C(21)-C(20)	119.9(2)	C(29)-C(28)-P(1)	117.1(2)
C(16)-C(21)-P(1)	117.53(19)	C(30)-C(29)-C(28)	120.9(3)
C(20)-C(21)-P(1)	122.5(2)	C(31)-C(30)-C(29)	120.1(3)
C(27)-C(22)-C(23)	118.4(2)	C(32)-C(31)-C(30)	119.8(3)
C(27)-C(22)-P(1)	124.3(2)	C(31)-C(32)-C(33)	120.4(3)
C(23)-C(22)-P(1)	117.3(2)	C(32)-C(33)-C(28)	120.7(3)
C(24)-C(23)-C(22)	120.7(3)		

Crystal structure of **4**

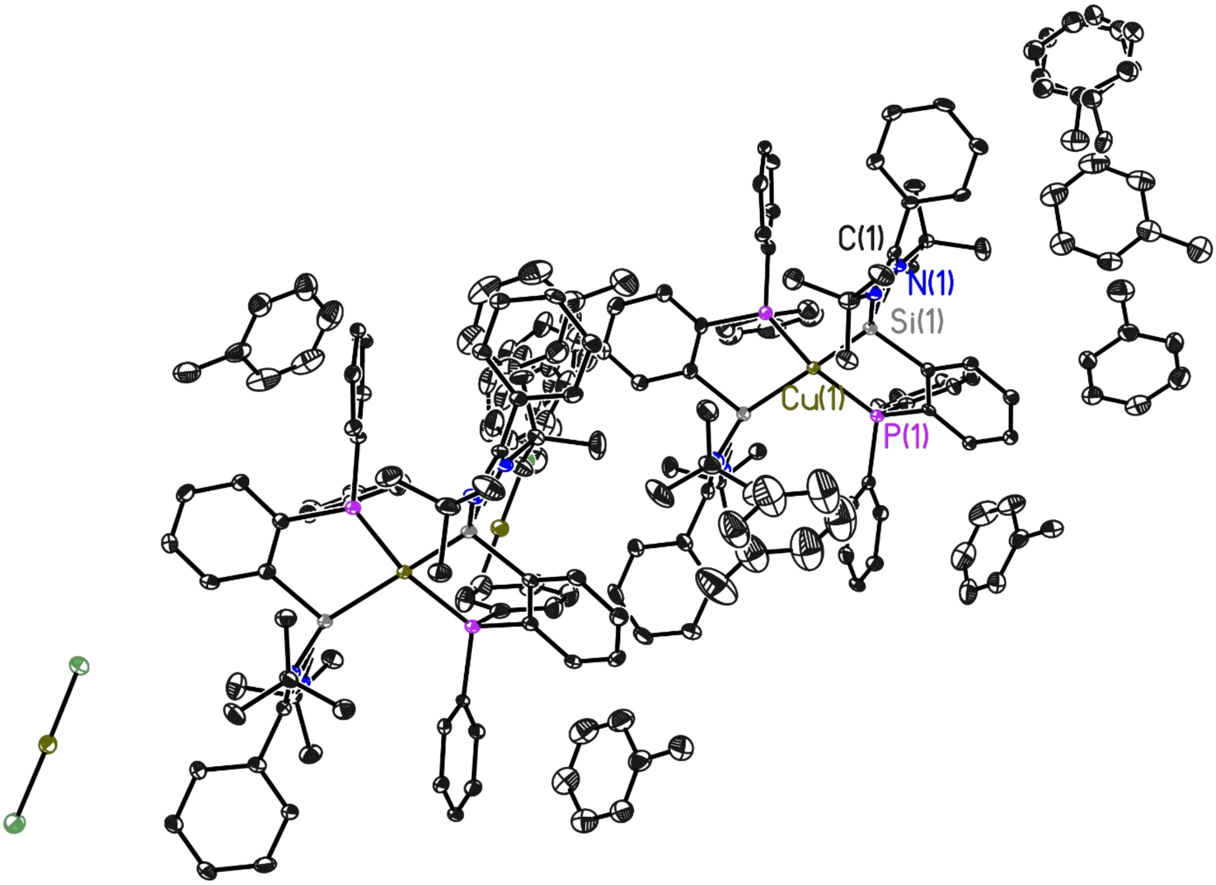

Figure 2 Molecular structure of **4** with thermal ellipsoids at 50% probability level. The hydrogen atoms are omitted for clarity.

The structure crystallizes as a non-merohedral twin. The fractional contribution of the minor domain refined to 0.4155(8). In the asymmetric unit there are nine toluene molecules, one of them is disordered about two positions and one about three positions. They were refined with distance restraints and restraints for the anisotropic displacement parameters. The major positions refined to 0.55(2), and 0.413(3) and 0.341(3).

Table 5. Bond lengths [Å] and angles [°] for **4**.

Si(1)-N(2)	1.838(5)
Si(1)-N(1)	1.867(4)
Si(1)-C(16)	1.883(5)
Si(1)-Cu(1)	2.2637(16)
Si(1)-C(1)	2.305(5)
P(1)-C(21)	1.832(5)
P(1)-C(28)	1.833(5)
P(1)-C(22)	1.835(6)
P(1)-Cu(1)	2.2982(15)
N(1)-C(1)	1.310(7)
N(1)-C(6)	1.483(7)
C(1)-N(2)	1.336(7)
C(1)-C(10)	1.496(7)
Cu(1)-Si(2)	2.2728(16)
Cu(1)-P(2)	2.2807(16)
Si(2)-N(4)	1.841(5)
Si(2)-N(3)	1.857(4)
Si(2)-C(49)	1.898(5)
Si(2)-C(34)	2.302(6)
P(2)-C(61)	1.809(6)
P(2)-C(55)	1.836(5)
P(2)-C(54)	1.840(5)

Cu(2)-Cl(1)	2.0988(16)	N(8)-C(67)	1.339(7)
Cu(2)-Cl(2)	2.1040(17)	N(8)-C(68)	1.469(7)
N(2)-C(2)	1.484(7)	C(10)-C(15)	1.389(7)
C(2)-C(5)	1.508(8)	C(10)-C(11)	1.392(7)
C(2)-C(4)	1.524(8)	C(11)-C(12)	1.389(8)
C(2)-C(3)	1.534(8)	C(12)-C(13)	1.378(8)
Si(3)-N(6)	1.832(5)	C(13)-C(14)	1.377(8)
Si(3)-N(5)	1.851(4)	C(14)-C(15)	1.373(7)
Si(3)-C(115)	1.882(5)	C(16)-C(17)	1.397(7)
Si(3)-Cu(3)	2.2696(16)	C(16)-C(21)	1.417(7)
Si(3)-C(100)	2.303(5)	C(17)-C(18)	1.374(7)
P(3)-C(121)	1.824(6)	C(18)-C(19)	1.388(7)
P(3)-C(127)	1.837(5)	C(19)-C(20)	1.378(7)
P(3)-C(120)	1.841(5)	C(20)-C(21)	1.391(7)
P(3)-Cu(3)	2.2791(15)	C(22)-C(23)	1.379(8)
Cl(3)-Cu(4)	2.0970(16)	C(22)-C(27)	1.396(7)
N(3)-C(34)	1.315(8)	C(23)-C(24)	1.375(8)
N(3)-C(35)	1.482(8)	C(24)-C(25)	1.389(8)
Cu(3)-Si(4)	2.2701(15)	C(25)-C(26)	1.372(9)
Cu(3)-P(4)	2.3065(15)	C(26)-C(27)	1.372(8)
Si(4)-N(8)	1.850(4)	C(28)-C(33)	1.390(7)
Si(4)-N(7)	1.851(4)	C(28)-C(29)	1.392(7)
Si(4)-C(82)	1.899(5)	C(29)-C(30)	1.390(8)
Si(4)-C(67)	2.311(5)	C(30)-C(31)	1.371(8)
P(4)-C(88)	1.826(6)	C(31)-C(32)	1.389(8)
P(4)-C(94)	1.831(5)	C(32)-C(33)	1.398(7)
P(4)-C(87)	1.848(5)	C(34)-C(43)	1.499(7)
Cl(4)-Cu(4)	2.0951(15)	C(35)-C(38)	1.517(8)
N(4)-C(34)	1.344(8)	C(35)-C(37)	1.530(8)
N(4)-C(39)	1.471(8)	C(35)-C(36)	1.543(7)
N(5)-C(100)	1.311(7)	C(39)-C(41)	1.515(9)
N(5)-C(101)	1.491(7)	C(39)-C(42)	1.526(10)
C(6)-C(9)	1.518(7)	C(39)-C(40)	1.529(9)
C(6)-C(7)	1.521(8)	C(43)-C(44)	1.386(8)
C(6)-C(8)	1.528(7)	C(43)-C(48)	1.392(8)
N(6)-C(100)	1.345(6)	C(44)-C(45)	1.376(8)
N(6)-C(105)	1.452(7)	C(45)-C(46)	1.371(9)
N(7)-C(67)	1.324(7)	C(46)-C(47)	1.390(9)
N(7)-C(72)	1.483(7)	C(47)-C(48)	1.382(8)

C(49)-C(54)	1.389(8)	C(89)-C(90)	1.379(8)
C(49)-C(50)	1.414(7)	C(90)-C(91)	1.353(8)
C(50)-C(51)	1.364(8)	C(91)-C(92)	1.375(9)
C(51)-C(52)	1.377(9)	C(92)-C(93)	1.389(9)
C(52)-C(53)	1.378(8)	C(94)-C(99)	1.386(8)
C(53)-C(54)	1.400(7)	C(94)-C(95)	1.387(7)
C(55)-C(60)	1.374(8)	C(95)-C(96)	1.394(7)
C(55)-C(56)	1.389(8)	C(96)-C(97)	1.383(9)
C(56)-C(57)	1.403(8)	C(97)-C(98)	1.365(8)
C(57)-C(58)	1.354(9)	C(98)-C(99)	1.385(7)
C(58)-C(59)	1.370(8)	C(100)-C(109)	1.495(7)
C(59)-C(60)	1.387(7)	C(101)-C(102)	1.499(8)
C(61)-C(62)	1.377(8)	C(101)-C(104)	1.515(7)
C(61)-C(66)	1.398(8)	C(101)-C(103)	1.528(8)
C(62)-C(63)	1.375(9)	C(105)-C(108)	1.516(9)
C(63)-C(64)	1.385(10)	C(105)-C(107)	1.521(8)
C(64)-C(65)	1.367(10)	C(105)-C(106)	1.534(8)
C(65)-C(66)	1.380(10)	C(109)-C(110)	1.378(7)
C(67)-C(76)	1.490(7)	C(109)-C(114)	1.387(7)
C(68)-C(71)	1.521(7)	C(110)-C(111)	1.390(7)
C(68)-C(69)	1.539(7)	C(111)-C(112)	1.378(8)
C(68)-C(70)	1.540(7)	C(112)-C(113)	1.375(8)
C(72)-C(75)	1.523(8)	C(113)-C(114)	1.372(7)
C(72)-C(74)	1.525(7)	C(115)-C(120)	1.399(7)
C(72)-C(73)	1.531(7)	C(115)-C(116)	1.410(7)
C(76)-C(77)	1.376(7)	C(116)-C(117)	1.367(7)
C(76)-C(81)	1.403(7)	C(117)-C(118)	1.376(8)
C(77)-C(78)	1.374(7)	C(118)-C(119)	1.372(8)
C(78)-C(79)	1.389(8)	C(119)-C(120)	1.401(7)
C(79)-C(80)	1.373(7)	C(121)-C(126)	1.382(8)
C(80)-C(81)	1.388(7)	C(121)-C(122)	1.386(8)
C(82)-C(87)	1.390(7)	C(122)-C(123)	1.392(10)
C(82)-C(83)	1.404(7)	C(123)-C(124)	1.348(10)
C(83)-C(84)	1.372(7)	C(124)-C(125)	1.350(9)
C(84)-C(85)	1.391(8)	C(125)-C(126)	1.367(9)
C(85)-C(86)	1.374(7)	C(127)-C(132)	1.380(7)
C(86)-C(87)	1.402(7)	C(127)-C(128)	1.387(7)
C(88)-C(93)	1.377(8)	C(128)-C(129)	1.388(7)
C(88)-C(89)	1.397(7)	C(129)-C(130)	1.375(8)

C(130)-C(131)	1.376(8)	N(4)-Si(2)-C(34)	35.7(2)
C(131)-C(132)	1.395(7)	N(3)-Si(2)-C(34)	34.8(2)
N(2)-Si(1)-N(1)	70.0(2)	C(49)-Si(2)-C(34)	104.6(2)
N(2)-Si(1)-C(16)	103.9(2)	Cu(1)-Si(2)-C(34)	147.96(14)
N(1)-Si(1)-C(16)	101.2(2)	C(61)-P(2)-C(55)	105.9(2)
N(2)-Si(1)-Cu(1)	139.11(15)	C(61)-P(2)-C(54)	103.5(3)
N(1)-Si(1)-Cu(1)	128.60(17)	C(55)-P(2)-C(54)	99.7(2)
C(16)-Si(1)-Cu(1)	106.21(16)	C(61)-P(2)-Cu(1)	118.28(18)
N(2)-Si(1)-C(1)	35.42(19)	C(55)-P(2)-Cu(1)	119.51(18)
N(1)-Si(1)-C(1)	34.6(2)	C(54)-P(2)-Cu(1)	107.21(19)
C(16)-Si(1)-C(1)	105.8(2)	Cl(1)-Cu(2)-Cl(2)	178.25(9)
Cu(1)-Si(1)-C(1)	146.91(14)	C(1)-N(2)-C(2)	131.8(5)
C(21)-P(1)-C(28)	99.6(2)	C(1)-N(2)-Si(1)	91.7(4)
C(21)-P(1)-C(22)	103.0(2)	C(2)-N(2)-Si(1)	134.9(3)
C(28)-P(1)-C(22)	104.8(2)	N(2)-C(2)-C(5)	112.6(5)
C(21)-P(1)-Cu(1)	106.46(17)	N(2)-C(2)-C(4)	109.6(5)
C(28)-P(1)-Cu(1)	117.07(16)	C(5)-C(2)-C(4)	111.0(5)
C(22)-P(1)-Cu(1)	122.57(17)	N(2)-C(2)-C(3)	105.5(4)
C(1)-N(1)-C(6)	132.1(4)	C(5)-C(2)-C(3)	108.8(5)
C(1)-N(1)-Si(1)	91.3(3)	C(4)-C(2)-C(3)	109.2(5)
C(6)-N(1)-Si(1)	135.5(4)	N(6)-Si(3)-N(5)	70.4(2)
N(1)-C(1)-N(2)	106.9(5)	N(6)-Si(3)-C(115)	102.9(2)
N(1)-C(1)-C(10)	125.6(5)	N(5)-Si(3)-C(115)	100.7(2)
N(2)-C(1)-C(10)	127.4(5)	N(6)-Si(3)-Cu(3)	138.67(15)
N(1)-C(1)-Si(1)	54.1(3)	N(5)-Si(3)-Cu(3)	129.86(16)
N(2)-C(1)-Si(1)	52.9(3)	C(115)-Si(3)-Cu(3)	106.40(16)
C(10)-C(1)-Si(1)	179.6(5)	N(6)-Si(3)-C(100)	35.72(19)
Si(1)-Cu(1)-Si(2)	135.45(7)	N(5)-Si(3)-C(100)	34.7(2)
Si(1)-Cu(1)-P(2)	111.09(6)	C(115)-Si(3)-C(100)	104.8(2)
Si(2)-Cu(1)-P(2)	87.34(6)	Cu(3)-Si(3)-C(100)	147.99(14)
Si(1)-Cu(1)-P(1)	87.68(6)	C(121)-P(3)-C(127)	103.8(2)
Si(2)-Cu(1)-P(1)	111.31(6)	C(121)-P(3)-C(120)	106.4(2)
P(2)-Cu(1)-P(1)	130.36(6)	C(127)-P(3)-C(120)	98.8(2)
N(4)-Si(2)-N(3)	70.6(2)	C(121)-P(3)-Cu(3)	118.10(18)
N(4)-Si(2)-C(49)	103.4(2)	C(127)-P(3)-Cu(3)	119.89(16)
N(3)-Si(2)-C(49)	100.0(2)	C(120)-P(3)-Cu(3)	107.42(17)
N(4)-Si(2)-Cu(1)	140.41(17)	C(34)-N(3)-C(35)	132.2(4)
N(3)-Si(2)-Cu(1)	128.23(16)	C(34)-N(3)-Si(2)	91.4(4)
C(49)-Si(2)-Cu(1)	105.86(17)	C(35)-N(3)-Si(2)	135.7(4)

Si(3)-Cu(3)-Si(4)	135.54(7)	C(67)-N(7)-Si(4)	91.9(3)
Si(3)-Cu(3)-P(3)	87.02(6)	C(72)-N(7)-Si(4)	136.2(4)
Si(4)-Cu(3)-P(3)	110.79(5)	C(67)-N(8)-C(68)	132.5(4)
Si(3)-Cu(3)-P(4)	111.32(6)	C(67)-N(8)-Si(4)	91.5(3)
Si(4)-Cu(3)-P(4)	87.54(6)	C(68)-N(8)-Si(4)	134.4(3)
P(3)-Cu(3)-P(4)	131.32(6)	C(15)-C(10)-C(11)	119.2(5)
N(8)-Si(4)-N(7)	70.3(2)	C(15)-C(10)-C(1)	119.5(4)
N(8)-Si(4)-C(82)	103.1(2)	C(11)-C(10)-C(1)	121.2(5)
N(7)-Si(4)-C(82)	101.9(2)	C(12)-C(11)-C(10)	120.0(5)
N(8)-Si(4)-Cu(3)	139.43(15)	C(13)-C(12)-C(11)	120.0(5)
N(7)-Si(4)-Cu(3)	128.38(15)	C(14)-C(13)-C(12)	119.8(5)
C(82)-Si(4)-Cu(3)	106.13(16)	C(15)-C(14)-C(13)	120.8(5)
N(8)-Si(4)-C(67)	35.38(19)	C(14)-C(15)-C(10)	120.1(5)
N(7)-Si(4)-C(67)	34.94(19)	C(17)-C(16)-C(21)	117.4(4)
C(82)-Si(4)-C(67)	106.0(2)	C(17)-C(16)-Si(1)	124.9(4)
Cu(3)-Si(4)-C(67)	146.95(13)	C(21)-C(16)-Si(1)	117.7(4)
C(88)-P(4)-C(94)	104.1(2)	C(18)-C(17)-C(16)	121.8(5)
C(88)-P(4)-C(87)	104.2(2)	C(17)-C(18)-C(19)	120.2(5)
C(94)-P(4)-C(87)	99.1(2)	C(20)-C(19)-C(18)	119.6(5)
C(88)-P(4)-Cu(3)	123.63(17)	C(19)-C(20)-C(21)	120.8(5)
C(94)-P(4)-Cu(3)	116.41(18)	C(20)-C(21)-C(16)	120.1(5)
C(87)-P(4)-Cu(3)	106.10(17)	C(20)-C(21)-P(1)	121.8(4)
C(34)-N(4)-C(39)	133.3(5)	C(16)-C(21)-P(1)	118.1(4)
C(34)-N(4)-Si(2)	91.2(4)	C(23)-C(22)-C(27)	117.4(5)
C(39)-N(4)-Si(2)	135.1(4)	C(23)-C(22)-P(1)	119.2(4)
Cl(4)-Cu(4)-Cl(3)	177.09(8)	C(27)-C(22)-P(1)	123.3(4)
C(100)-N(5)-C(101)	131.3(4)	C(24)-C(23)-C(22)	122.0(5)
C(100)-N(5)-Si(3)	91.8(3)	C(23)-C(24)-C(25)	119.7(6)
C(101)-N(5)-Si(3)	135.8(4)	C(26)-C(25)-C(24)	119.0(6)
N(1)-C(6)-C(9)	111.3(4)	C(27)-C(26)-C(25)	120.9(5)
N(1)-C(6)-C(7)	106.7(4)	C(26)-C(27)-C(22)	120.9(5)
C(9)-C(6)-C(7)	108.3(5)	C(33)-C(28)-C(29)	118.8(5)
N(1)-C(6)-C(8)	109.4(4)	C(33)-C(28)-P(1)	116.1(4)
C(9)-C(6)-C(8)	111.3(5)	C(29)-C(28)-P(1)	124.9(4)
C(7)-C(6)-C(8)	109.8(5)	C(30)-C(29)-C(28)	120.7(6)
C(100)-N(6)-C(105)	132.4(5)	C(31)-C(30)-C(29)	120.2(5)
C(100)-N(6)-Si(3)	91.6(4)	C(30)-C(31)-C(32)	120.1(5)
C(105)-N(6)-Si(3)	135.1(3)	C(31)-C(32)-C(33)	119.9(6)
C(67)-N(7)-C(72)	130.5(4)	C(28)-C(33)-C(32)	120.2(5)

N(3)-C(34)-N(4)	106.9(5)	C(55)-C(56)-C(57)	118.6(6)
N(3)-C(34)-C(43)	125.4(6)	C(58)-C(57)-C(56)	121.5(6)
N(4)-C(34)-C(43)	127.7(6)	C(57)-C(58)-C(59)	119.7(5)
N(3)-C(34)-Si(2)	53.7(3)	C(58)-C(59)-C(60)	119.9(6)
N(4)-C(34)-Si(2)	53.1(3)	C(55)-C(60)-C(59)	120.9(5)
C(43)-C(34)-Si(2)	179.1(5)	C(62)-C(61)-C(66)	117.8(6)
N(3)-C(35)-C(38)	106.0(4)	C(62)-C(61)-P(2)	119.0(5)
N(3)-C(35)-C(37)	110.6(5)	C(66)-C(61)-P(2)	123.0(5)
C(38)-C(35)-C(37)	109.5(5)	C(63)-C(62)-C(61)	122.0(7)
N(3)-C(35)-C(36)	111.2(5)	C(62)-C(63)-C(64)	119.6(7)
C(38)-C(35)-C(36)	108.3(4)	C(65)-C(64)-C(63)	119.3(7)
C(37)-C(35)-C(36)	111.2(5)	C(64)-C(65)-C(66)	121.2(7)
N(4)-C(39)-C(41)	106.3(5)	C(65)-C(66)-C(61)	120.1(7)
N(4)-C(39)-C(42)	109.5(5)	N(7)-C(67)-N(8)	106.3(4)
C(41)-C(39)-C(42)	109.1(6)	N(7)-C(67)-C(76)	127.5(5)
N(4)-C(39)-C(40)	111.7(6)	N(8)-C(67)-C(76)	126.3(5)
C(41)-C(39)-C(40)	109.3(5)	N(7)-C(67)-Si(4)	53.2(2)
C(42)-C(39)-C(40)	110.8(6)	N(8)-C(67)-Si(4)	53.1(3)
C(44)-C(43)-C(48)	119.5(5)	C(76)-C(67)-Si(4)	179.4(4)
C(44)-C(43)-C(34)	119.8(5)	N(8)-C(68)-C(71)	106.0(4)
C(48)-C(43)-C(34)	120.5(5)	N(8)-C(68)-C(69)	112.6(4)
C(45)-C(44)-C(43)	121.0(6)	C(71)-C(68)-C(69)	109.2(4)
C(46)-C(45)-C(44)	119.6(6)	N(8)-C(68)-C(70)	109.6(4)
C(45)-C(46)-C(47)	120.2(5)	C(71)-C(68)-C(70)	109.8(5)
C(48)-C(47)-C(46)	120.5(6)	C(69)-C(68)-C(70)	109.6(5)
C(47)-C(48)-C(43)	119.2(6)	N(7)-C(72)-C(75)	106.3(4)
C(54)-C(49)-C(50)	117.9(5)	N(7)-C(72)-C(74)	109.2(4)
C(54)-C(49)-Si(2)	118.0(4)	C(75)-C(72)-C(74)	110.8(5)
C(50)-C(49)-Si(2)	124.1(4)	N(7)-C(72)-C(73)	111.3(4)
C(51)-C(50)-C(49)	121.1(6)	C(75)-C(72)-C(73)	108.3(5)
C(50)-C(51)-C(52)	120.4(5)	C(74)-C(72)-C(73)	110.8(5)
C(51)-C(52)-C(53)	120.4(6)	C(77)-C(76)-C(81)	119.9(5)
C(52)-C(53)-C(54)	119.6(6)	C(77)-C(76)-C(67)	120.4(4)
C(49)-C(54)-C(53)	120.7(5)	C(81)-C(76)-C(67)	119.7(4)
C(49)-C(54)-P(2)	117.9(4)	C(78)-C(77)-C(76)	120.5(5)
C(53)-C(54)-P(2)	121.3(4)	C(77)-C(78)-C(79)	119.9(5)
C(60)-C(55)-C(56)	119.3(5)	C(80)-C(79)-C(78)	120.2(5)
C(60)-C(55)-P(2)	116.8(4)	C(79)-C(80)-C(81)	120.3(5)
C(56)-C(55)-P(2)	123.8(4)	C(80)-C(81)-C(76)	119.2(5)

C(87)-C(82)-C(83)	118.1(5)	N(6)-C(105)-C(107)	111.3(5)
C(87)-C(82)-Si(4)	117.7(4)	C(108)-C(105)-C(107)	110.7(5)
C(83)-C(82)-Si(4)	124.2(4)	N(6)-C(105)-C(106)	104.9(4)
C(84)-C(83)-C(82)	121.6(5)	C(108)-C(105)-C(106)	107.7(5)
C(83)-C(84)-C(85)	119.4(5)	C(107)-C(105)-C(106)	109.1(5)
C(86)-C(85)-C(84)	120.4(5)	C(110)-C(109)-C(114)	119.7(5)
C(85)-C(86)-C(87)	120.0(5)	C(110)-C(109)-C(100)	120.0(4)
C(82)-C(87)-C(86)	120.4(5)	C(114)-C(109)-C(100)	120.2(4)
C(82)-C(87)-P(4)	118.8(4)	C(109)-C(110)-C(111)	120.0(5)
C(86)-C(87)-P(4)	120.8(4)	C(112)-C(111)-C(110)	119.8(5)
C(93)-C(88)-C(89)	117.8(5)	C(113)-C(112)-C(111)	120.0(5)
C(93)-C(88)-P(4)	119.1(4)	C(114)-C(113)-C(112)	120.4(5)
C(89)-C(88)-P(4)	123.1(4)	C(113)-C(114)-C(109)	120.0(5)
C(90)-C(89)-C(88)	120.5(5)	C(120)-C(115)-C(116)	116.9(4)
C(91)-C(90)-C(89)	120.3(6)	C(120)-C(115)-Si(3)	117.9(4)
C(90)-C(91)-C(92)	121.1(6)	C(116)-C(115)-Si(3)	125.2(4)
C(91)-C(92)-C(93)	118.6(6)	C(117)-C(116)-C(115)	122.2(5)
C(88)-C(93)-C(92)	121.7(6)	C(116)-C(117)-C(118)	119.9(5)
C(99)-C(94)-C(95)	118.0(5)	C(119)-C(118)-C(117)	120.2(5)
C(99)-C(94)-P(4)	116.8(4)	C(118)-C(119)-C(120)	120.4(5)
C(95)-C(94)-P(4)	125.0(4)	C(115)-C(120)-C(119)	120.5(5)
C(94)-C(95)-C(96)	120.5(6)	C(115)-C(120)-P(3)	117.5(4)
C(97)-C(96)-C(95)	120.3(5)	C(119)-C(120)-P(3)	122.0(4)
C(98)-C(97)-C(96)	119.6(5)	C(126)-C(121)-C(122)	118.0(5)
C(97)-C(98)-C(99)	120.2(6)	C(126)-C(121)-P(3)	117.0(4)
C(98)-C(99)-C(94)	121.4(5)	C(122)-C(121)-P(3)	125.0(5)
N(5)-C(100)-N(6)	106.2(4)	C(121)-C(122)-C(123)	119.6(6)
N(5)-C(100)-C(109)	127.6(5)	C(124)-C(123)-C(122)	119.6(6)
N(6)-C(100)-C(109)	126.2(5)	C(123)-C(124)-C(125)	122.3(7)
N(5)-C(100)-Si(3)	53.5(3)	C(124)-C(125)-C(126)	118.4(7)
N(6)-C(100)-Si(3)	52.7(3)	C(125)-C(126)-C(121)	122.0(6)
C(109)-C(100)-Si(3)	178.4(4)	C(132)-C(127)-C(128)	119.3(4)
N(5)-C(101)-C(102)	106.2(4)	C(132)-C(127)-P(3)	124.2(4)
N(5)-C(101)-C(104)	111.0(4)	C(128)-C(127)-P(3)	116.3(4)
C(102)-C(101)-C(104)	109.7(5)	C(127)-C(128)-C(129)	120.6(5)
N(5)-C(101)-C(103)	109.7(5)	C(130)-C(129)-C(128)	119.8(6)
C(102)-C(101)-C(103)	108.9(5)	C(129)-C(130)-C(131)	119.9(5)
C(104)-C(101)-C(103)	111.2(5)	C(130)-C(131)-C(132)	120.6(5)
N(6)-C(105)-C(108)	112.9(5)	C(127)-C(132)-C(131)	119.8(5)

S1.5. Crystal structure of **5**

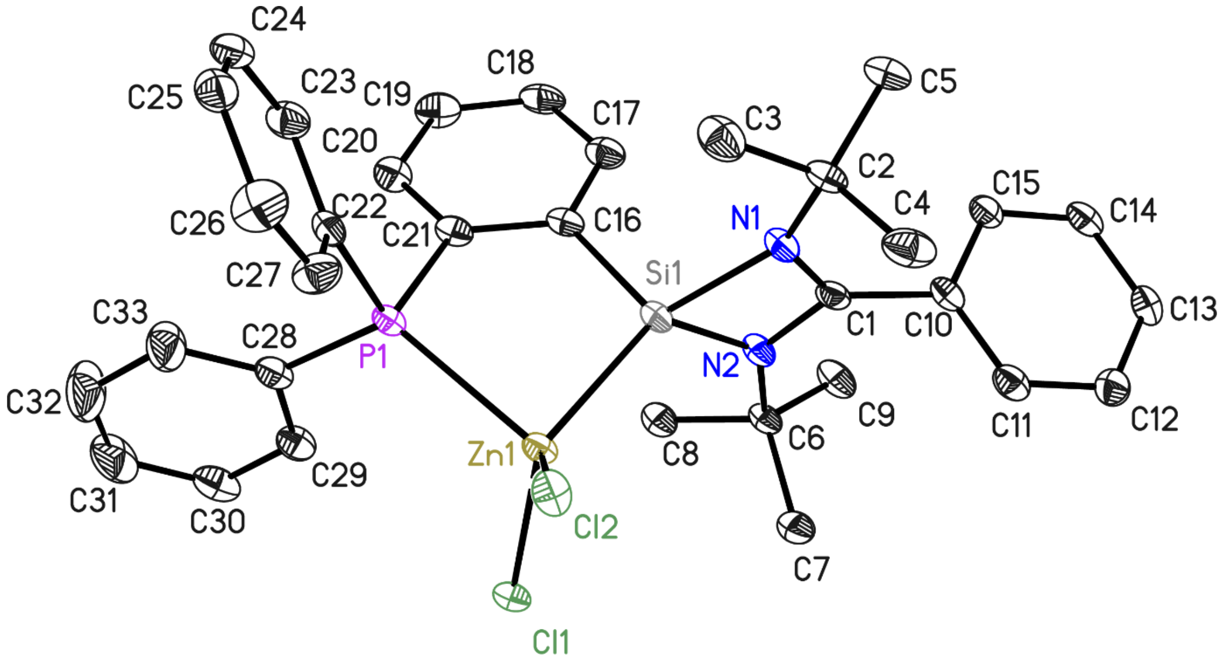

Figure 5 Molecular structure of **5** with thermal ellipsoids at 50% probability level. The hydrogen atoms are omitted for clarity

Table 6. Bond lengths [Å] and angles [°] for **5**.

Zn(1)-Cl(2)	2.2463(10)
Zn(1)-Cl(1)	2.2661(7)
Zn(1)-Si(1)	2.3947(8)
Zn(1)-P(1)	2.4812(8)
P(1)-C(28)	1.821(2)
P(1)-C(22)	1.821(2)
P(1)-C(21)	1.838(2)
Si(1)-N(2)	1.820(2)
Si(1)-N(1)	1.8231(19)
Si(1)-C(16)	1.879(2)
Si(1)-C(1)	2.278(2)
N(1)-C(1)	1.339(3)
N(1)-C(2)	1.486(3)
C(1)-N(2)	1.340(3)
C(1)-C(10)	1.483(3)
N(2)-C(6)	1.488(3)
C(2)-C(3)	1.519(3)
C(2)-C(4)	1.523(3)
C(2)-C(5)	1.529(3)
C(6)-C(9)	1.524(3)
C(6)-C(7)	1.524(3)
C(6)-C(8)	1.527(3)
C(10)-C(11)	1.387(3)
C(10)-C(15)	1.397(3)
C(11)-C(12)	1.379(3)
C(12)-C(13)	1.384(3)
C(13)-C(14)	1.385(3)
C(14)-C(15)	1.384(3)
C(16)-C(17)	1.406(3)
C(16)-C(21)	1.410(3)
C(20)-C(19)	1.384(3)
C(20)-C(21)	1.398(3)
C(17)-C(18)	1.382(3)
C(19)-C(18)	1.388(4)
C(22)-C(23)	1.385(3)
C(22)-C(27)	1.393(3)

C(23)-C(24)	1.386(3)	N(2)-C(1)-Si(1)	53.01(11)
C(24)-C(25)	1.382(4)	C(10)-C(1)-Si(1)	173.73(16)
C (25)-C(26)	1.374(4)	C(1)-N(2)-C(6)	132.28(19)
C(26)-C(27)	1.382(4)	C(1)-N(2)-Si(1)	90.96(14)
C(28)-C(33)	1.383(4)	C(6)-N(2)-Si(1)	136.09(14)
C(28)-C(29)	1.392(3)	N(1)-C(2)-C(3)	105.75(18)
C(29)-C(30)	1.388(3)	N(1)-C(2)-C(4)	110.58(18)
C(30)-C(31)	1.372(4)	C(3)-C(2)-C(4)	109.8(2)
C(31)-C(32)	1.378(4)	N(1)-C(2)-C(5)	110.31(19)
C(32)-C(33)	1.393(4)	C(3)-C(2)-C(5)	109.9(2)
Cl(2)-Zn(1)-Cl(1)	112.36(3)	C(4)-C(2)-C(5)	110.36(19)
Cl(2)-Zn(1)-Si(1)	121.61(2)	N(2)-C(6)-C(9)	112.55(18)
Cl(1)-Zn(1)-Si(1)	113.69(3)	N(2)-C(6)-C(7)	109.74(18)
Cl(2)-Zn(1)-P(1)	114.78(3)	C(9)-C(6)-C(7)	110.58(19)
Cl(1)-Zn(1)-P(1)	104.97(3)	N(2)-C(6)-C(8)	104.68(18)
Si(1)-Zn(1)-P(1)	85.41(2)	C(9)-C(6)-C(8)	109.49(19)
C(28)-P(1)-C(22)	105.62(11)	C(7)-C(6)-C(8)	109.62(18)
C(28)-P(1)-C(21)	103.93(10)	C(11)-C(10)-C(15)	120.2(2)
C(22)-P(1)-C(21)	105.85(10)	C(11)-C(10)-C(1)	119.25(19)
C(28)-P(1)-Zn(1)	120.15(8)	C(15)-C(10)-C(1)	120.3(2)
C(22)-P(1)-Zn(1)	115.09(8)	C(12)-C(11)-C(10)	120.1(2)
C(21)-P(1)-Zn(1)	104.78(8)	C(11)-C(12)-C(13)	119.9(2)
N(2)-Si(1)-N(1)	71.88(9)	C(12)-C(13)-C(14)	120.4(2)
N(2)-Si(1)-C(16)	106.73(10)	C(15)-C(14)-C(13)	120.2(2)
N(1)-Si(1)-C(16)	109.55(9)	C(14)-C(15)-C(10)	119.3(2)
N(2)-Si(1)-C(1)	36.03(8)	C(17)-C(16)-C(21)	117.9(2)
N(1)-Si(1)-C(1)	35.99(8)	C(17)-C(16)-Si(1)	120.84(17)
C(16)-Si(1)-C(1)	115.04(9)	C(21)-C(16)-Si(1)	120.79(16)
N(2)-Si(1)-Zn(1)	125.27(6)	C(19)-C(20)-C(21)	120.8(2)
N(1)-Si(1)-Zn(1)	132.52(7)	C(18)-C(17)-C(16)	121.6(2)
C(16)-Si(1)-Zn(1)	106.14(7)	C(20)-C(19)-C(18)	119.9(2)
C(1)-Si(1)-Zn(1)	138.30(6)	C(17)-C(18)-C(19)	119.9(2)
C(1)-N(1)-C(2)	130.73(19)	C(23)-C(22)-C(27)	118.8(2)
C(1)-N(1)-Si(1)	90.87(14)	C(23)-C(22)-P(1)	124.42(18)
C(2)-N(1)-Si(1)	136.68(15)	C(27)-C(22)-P(1)	116.71(17)
N(1)-C(1)-N(2)	105.89(19)	C(20)-C(21)-C(16)	119.9(2)
N(1)-C(1)-C(10)	129.2(2)	C(20)-C(21)-P(1)	120.04(17)
N(2)-C(1)-C(10)	124.9(2)	C(16)-C(21)-P(1)	120.06(16)
N(1)-C(1)-Si(1)	53.14(11)	C(22)-C(23)-C(24)	120.4(2)

C(25)-C(24)-C(23)	120.4(2)	C(29)-C(28)-P(1)	118.23(18)
C(26)-C(25)-C(24)	119.6(2)	C(30)-C(29)-C(28)	120.7(2)
C(25)-C(26)-C(27)	120.4(2)	C(31)-C(30)-C(29)	119.9(2)
C(26)-C(27)-C(22)	120.5(2)	C(30)-C(31)-C(32)	120.1(3)
C(33)-C(28)-C(29)	118.9(2)	C(31)-C(32)-C(33)	120.4(3)
C(33)-C(28)-P(1)	122.83(19)	C(28)-C(33)-C(32)	120.1(3)

S1.6. Crystal structure of **6**

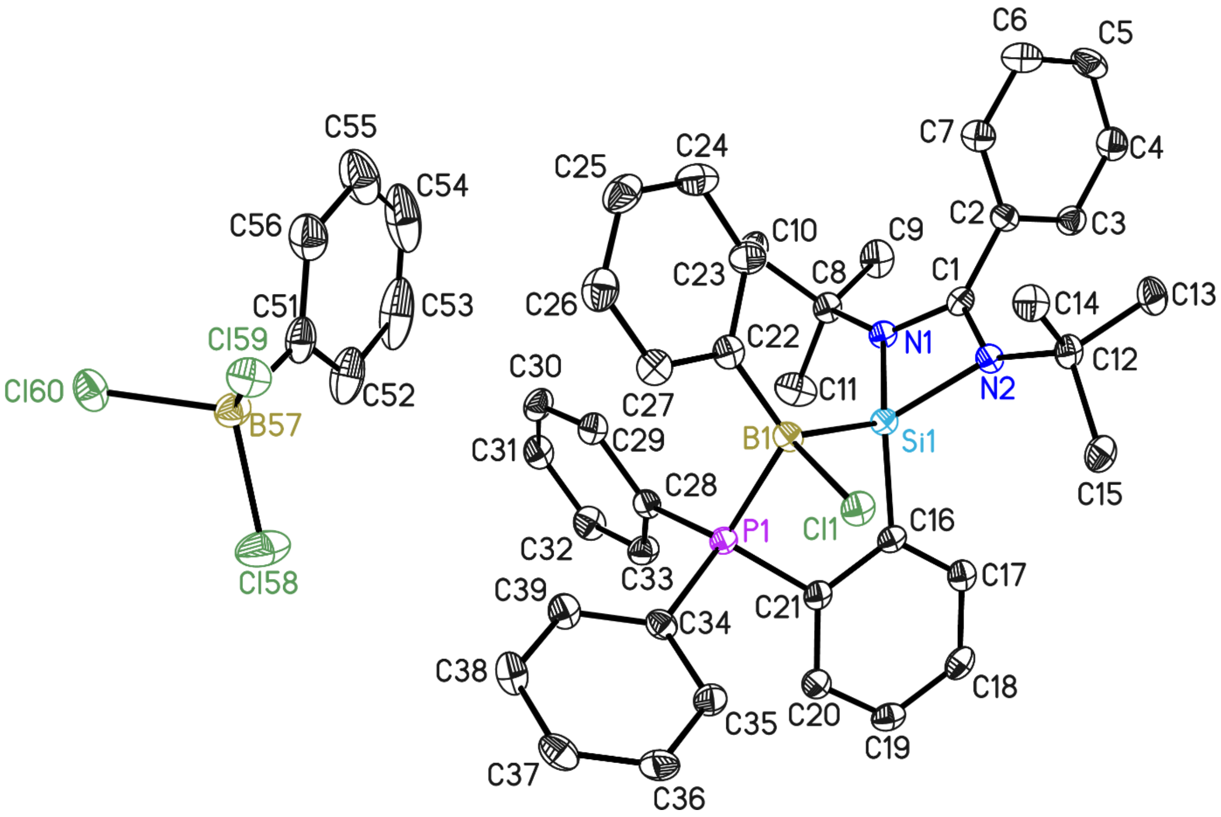

Figure 6 Molecular structure of **6** with thermal ellipsoids at 50% probability level. The hydrogen atoms are omitted for clarity.

The anisotropic displacement parameters of the carbon atoms of the BCl_3Ph anion were refined with restraints.

Table 7. Bond lengths [Å] and angles [°] **6**.

B(1)-C(22)	1.578(3)
B(1)-Cl(1)	1.904(2)
B(1)-P(1)	1.982(2)
B(1)-Si(1)	2.018(2)
B(57)-C(51)	1.588(3)
B(57)-Cl(58)	1.865(2)
B(57)-Cl(59)	1.882(2)
B(57)-Cl(60)	1.892(2)
P(1)-C(34)	1.803(2)
P(1)-C(28)	1.8076(19)
P(1)-C(21)	1.814(2)
Si(1)-N(2)	1.7945(17)
Si(1)-N(1)	1.8120(17)
Si(1)-C(16)	1.855(2)
Si(1)-C(1)	2.251(2)
N(1)-C(1)	1.337(2)
N(1)-C(8)	1.492(2)
N(2)-C(1)	1.348(2)
N(2)-C(12)	1.495(2)
C(1)-C(2)	1.477(3)
C(2)-C(7)	1.384(3)
C(2)-C(3)	1.387(3)
C(3)-C(4)	1.382(3)
C(4)-C(5)	1.382(3)

C(5)-C(6)	1.385(3)	C(22)-B(1)-P(1)	120.10(14)
C(6)-C(7)	1.379(3)	Cl(1)-B(1)-P(1)	99.11(10)
C(8)-C(11)	1.514(3)	C(22)-B(1)-Si(1)	124.83(14)
C(8)-C(9)	1.521(3)	Cl(1)-B(1)-Si(1)	101.69(10)
C(8)-C(10)	1.525(3)	P(1)-B(1)-Si(1)	95.33(10)
C(12)-C(14)	1.522(3)	C(51)-B(57)-Cl(58)	113.17(16)
C(12)-C(13)	1.523(3)	C(51)-B(57)-Cl(59)	111.80(15)
C(12)-C(15)	1.524(3)	Cl(58)-B(57)-Cl(59)	106.89(12)
C(16)-C(17)	1.396(3)	C(51)-B(57)-Cl(60)	110.05(14)
C(16)-C(21)	1.411(3)	Cl(58)-B(57)-Cl(60)	108.20(12)
C(17)-C(18)	1.385(3)	Cl(59)-B(57)-Cl(60)	106.43(12)
C(18)-C(19)	1.386(3)	C(34)-P(1)-C(28)	107.51(9)
C(19)-C(20)	1.393(3)	C(34)-P(1)-C(21)	110.67(9)
C(20)-C(21)	1.387(3)	C(28)-P(1)-C(21)	106.80(9)
C(22)-C(23)	1.393(3)	C(34)-P(1)-B(1)	115.32(9)
C(22)-C(27)	1.397(3)	C(28)-P(1)-B(1)	114.55(9)
C(23)-C(24)	1.394(3)	C(21)-P(1)-B(1)	101.57(9)
C(24)-C(25)	1.377(3)	N(2)-Si(1)-N(1)	73.09(8)
C(25)-C(26)	1.386(3)	N(2)-Si(1)-C(16)	117.77(9)
C(26)-C(27)	1.382(3)	N(1)-Si(1)-C(16)	116.98(8)
C(28)-C(33)	1.392(3)	N(2)-Si(1)-B(1)	123.11(8)
C(28)-C(29)	1.394(3)	N(1)-Si(1)-B(1)	125.06(9)
C(29)-C(30)	1.390(3)	C(16)-Si(1)-B(1)	100.86(9)
C(30)-C(31)	1.379(3)	N(2)-Si(1)-C(1)	36.78(7)
C(31)-C(32)	1.384(3)	N(1)-Si(1)-C(1)	36.45(7)
C(32)-C(33)	1.388(3)	C(16)-Si(1)-C(1)	127.31(8)
C(34)-C(39)	1.391(3)	B(1)-Si(1)-C(1)	131.82(8)
C(34)-C(35)	1.398(3)	C(1)-N(1)-C(8)	131.71(16)
C(35)-C(36)	1.384(3)	C(1)-N(1)-Si(1)	89.94(12)
C(36)-C(37)	1.382(3)	C(8)-N(1)-Si(1)	138.35(13)
C(37)-C(38)	1.386(3)	C(1)-N(2)-C(12)	131.92(16)
C(38)-C(39)	1.387(3)	C(1)-N(2)-Si(1)	90.35(12)
C(51)-C(56)	1.387(3)	C(12)-N(2)-Si(1)	135.23(13)
C(51)-C(52)	1.394(3)	N(1)-C(1)-N(2)	106.24(16)
C(52)-C(53)	1.407(4)	N(1)-C(1)-C(2)	127.54(17)
C(53)-C(54)	1.364(5)	N(2)-C(1)-C(2)	126.13(17)
C(54)-C(55)	1.370(4)	N(1)-C(1)-Si(1)	53.61(9)
C(55)-C(56)	1.392(3)	N(2)-C(1)-Si(1)	52.87(9)
C(22)-B(1)-Cl(1)	111.45(13)	C(2)-C(1)-Si(1)	172.89(14)

C(7)-C(2)-C(3)	120.73(18)	C(27)-C(22)-B(1)	121.52(18)
C(7)-C(2)-C(1)	118.67(18)	C(22)-C(23)-C(24)	122.3(2)
C(3)-C(2)-C(1)	120.57(17)	C(25)-C(24)-C(23)	119.6(2)
C(4)-C(3)-C(2)	119.10(19)	C(24)-C(25)-C(26)	119.6(2)
C(3)-C(4)-C(5)	120.3(2)	C(27)-C(26)-C(25)	120.1(2)
C(4)-C(5)-C(6)	120.3(2)	C(26)-C(27)-C(22)	122.0(2)
C(7)-C(6)-C(5)	119.8(2)	C(33)-C(28)-C(29)	119.80(18)
C(6)-C(7)-C(2)	119.8(2)	C(33)-C(28)-P(1)	120.10(15)
N(1)-C(8)-C(11)	105.76(16)	C(29)-C(28)-P(1)	120.10(15)
N(1)-C(8)-C(9)	112.70(16)	C(30)-C(29)-C(28)	119.55(19)
C(11)-C(8)-C(9)	109.07(18)	C(31)-C(30)-C(29)	120.41(19)
N(1)-C(8)-C(10)	108.69(16)	C(30)-C(31)-C(32)	120.29(19)
C(11)-C(8)-C(10)	110.69(18)	C(31)-C(32)-C(33)	119.9(2)
C(9)-C(8)-C(10)	109.88(17)	C(32)-C(33)-C(28)	120.10(19)
N(2)-C(12)-C(14)	108.34(16)	C(39)-C(34)-C(35)	120.03(18)
N(2)-C(12)-C(13)	112.81(16)	C(39)-C(34)-P(1)	118.99(15)
C(14)-C(12)-C(13)	109.58(17)	C(35)-C(34)-P(1)	120.73(15)
N(2)-C(12)-C(15)	105.60(15)	C(36)-C(35)-C(34)	119.54(19)
C(14)-C(12)-C(15)	111.45(16)	C(37)-C(36)-C(35)	120.26(19)
C(13)-C(12)-C(15)	109.04(17)	C(36)-C(37)-C(38)	120.44(19)
C(17)-C(16)-C(21)	118.53(17)	C(37)-C(38)-C(39)	119.8(2)
C(17)-C(16)-Si(1)	127.26(15)	C(38)-C(39)-C(34)	119.89(19)
C(21)-C(16)-Si(1)	114.13(14)	C(56)-C(51)-C(52)	116.8(2)
C(18)-C(17)-C(16)	120.47(18)	C(56)-C(51)-B(57)	120.3(2)
C(17)-C(18)-C(19)	120.65(19)	C(52)-C(51)-B(57)	122.7(2)
C(18)-C(19)-C(20)	119.84(19)	C(51)-C(52)-C(53)	120.4(3)
C(21)-C(20)-C(19)	119.82(19)	C(54)-C(53)-C(52)	121.0(3)
C(20)-C(21)-C(16)	120.67(18)	C(53)-C(54)-C(55)	119.7(2)
C(20)-C(21)-P(1)	123.65(15)	C(54)-C(55)-C(56)	119.6(3)
C(16)-C(21)-P(1)	115.64(14)	C(51)-C(56)-C(55)	122.6(2)
C(23)-C(22)-C(27)	116.41(19)		
C(23)-C(22)-B(1)	121.99(18)		

S1.7. Crystal structure of **7**

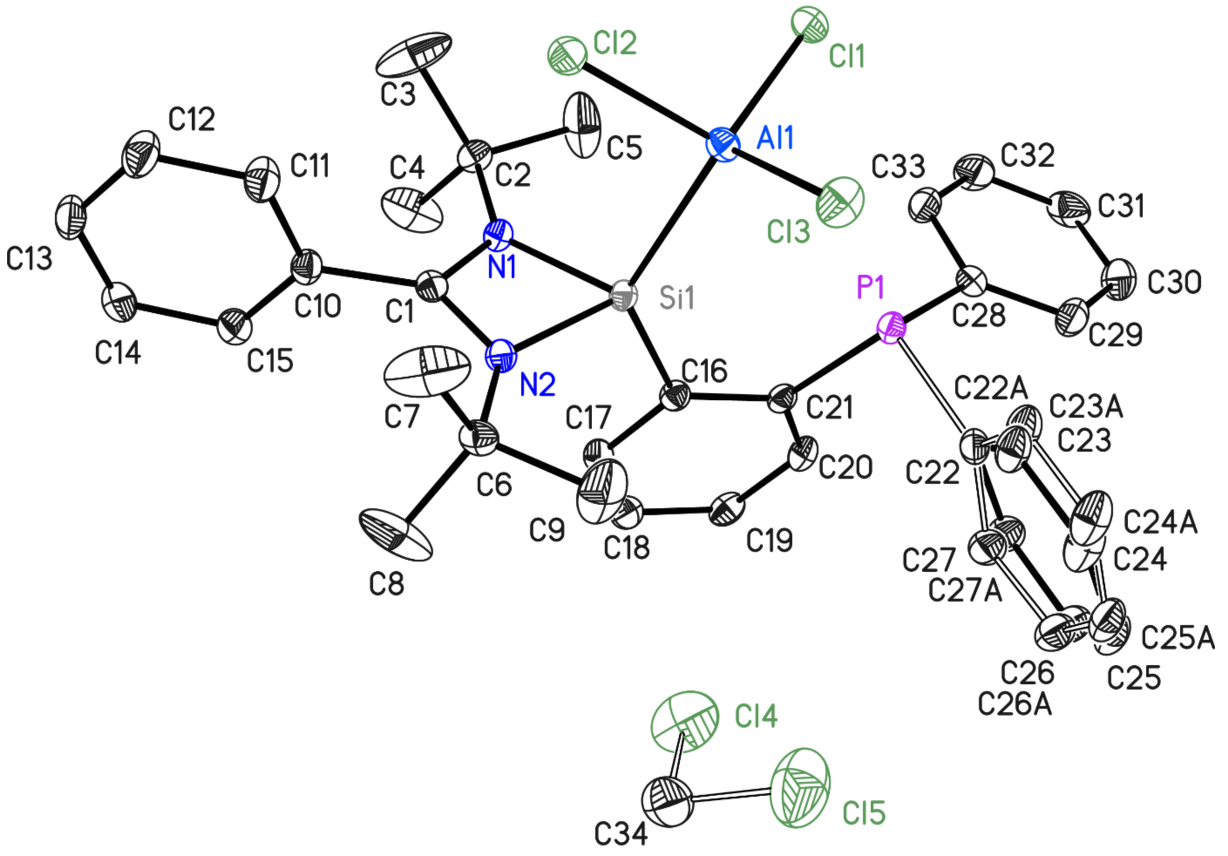

Figure 7 Molecular structure of **7** with thermal ellipsoids at 50% probability level. It consists of two fragments, one complex molecule and half a DCM solvent molecule on a special position. The hydrogen atoms are omitted for clarity.

One phenyl ring and the DCM molecule are disordered. For the refinement of the DCM molecule, distance restraints and restraints for the anisotropic displacement parameters were used. The occupancy of the main position of the phenyl ring refined to 0.790(17).

Table 8. Bond lengths [Å] and angles [°] for **7**

Cl(1)-Al(1)	2.1321(6)
Cl(2)-Al(1)	2.1751(6)
Cl(3)-Al(1)	2.1392(6)
Si(1)-N(2)	1.8149(12)
Si(1)-N(1)	1.8176(12)
Si(1)-C(16)	1.8705(14)
Si(1)-C(1)	2.2725(15)
Si(1)-Al(1)	2.4509(8)
N(1)-C(1)	1.3367(18)
N(1)-C(2)	1.4895(17)
N(2)-C(1)	1.3447(18)
N(2)-C(6)	1.4848(17)
C(1)-C(10)	1.4835(19)
C(2)-C(5)	1.512(2)
C(2)-C(4)	1.515(2)
C(2)-C(3)	1.519(2)
C(6)-C(7)	1.514(2)
C(6)-C(8)	1.515(2)
C(6)-C(9)	1.521(2)
C(10)-C(15)	1.388(2)
C(10)-C(11)	1.390(2)
C(11)-C(12)	1.386(2)

C(12)-C(13)	1.385(2)	N(2)-Si(1)-Al(1)	111.08(4)
C(13)-C(14)	1.381(2)	N(1)-Si(1)-Al(1)	108.19(4)
C(14)-C(15)	1.390(2)	C(16)-Si(1)-Al(1)	131.98(5)
C(16)-C(17)	1.406(2)	C(1)-Si(1)-Al(1)	111.71(4)
C(16)-C(21)	1.4088(19)	Cl(1)-Al(1)-Cl(3)	110.30(3)
C(17)-C(18)	1.384(2)	Cl(1)-Al(1)-Cl(2)	108.73(3)
C(18)-C(19)	1.392(2)	Cl(3)-Al(1)-Cl(2)	106.56(2)
C(19)-C(20)	1.387(2)	Cl(1)-Al(1)-Si(1)	115.04(2)
C(20)-C(21)	1.400(2)	Cl(3)-Al(1)-Si(1)	116.22(2)
C(21)-P(1)	1.8464(15)	Cl(2)-Al(1)-Si(1)	98.77(3)
P(1)-C(28)	1.8329(15)	C(1)-N(1)-C(2)	132.26(12)
P(1)-C(22A)	1.8412(15)	C(1)-N(1)-Si(1)	90.87(9)
P(1)-C(22)	1.8412(15)	C(2)-N(1)-Si(1)	136.48(9)
C(22)-C(23)	1.380(4)	C(1)-N(2)-C(6)	131.90(11)
C(22)-C(27)	1.417(3)	C(1)-N(2)-Si(1)	90.73(8)
C(23)-C(24)	1.402(4)	C(6)-N(2)-Si(1)	136.42(9)
C(24)-C(25)	1.383(4)	N(1)-C(1)-N(2)	105.75(11)
C(25)-C(26)	1.389(4)	N(1)-C(1)-C(10)	125.53(13)
C(26)-C(27)	1.390(3)	N(2)-C(1)-C(10)	128.70(12)
C(22A)-C(27A)	1.353(10)	N(1)-C(1)-Si(1)	53.11(7)
C(22A)-C(23A)	1.426(12)	N(2)-C(1)-Si(1)	53.00(7)
C(23A)-C(24A)	1.384(13)	C(10)-C(1)-Si(1)	173.24(10)
C(24A)-C(25A)	1.356(12)	N(1)-C(2)-C(5)	105.44(12)
C(25A)-C(26A)	1.372(12)	N(1)-C(2)-C(4)	109.68(12)
C(26A)-C(27A)	1.387(12)	C(5)-C(2)-C(4)	110.11(15)
C(28)-C(29)	1.389(2)	N(1)-C(2)-C(3)	111.89(12)
C(28)-C(33)	1.397(2)	C(5)-C(2)-C(3)	109.92(16)
C(29)-C(30)	1.392(2)	C(4)-C(2)-C(3)	109.73(16)
C(30)-C(31)	1.379(2)	N(2)-C(6)-C(7)	111.52(12)
C(31)-C(32)	1.387(2)	N(2)-C(6)-C(8)	109.86(12)
C(32)-C(33)	1.382(2)	C(7)-C(6)-C(8)	111.02(16)
C(34)-Cl(4)	1.728(5)	N(2)-C(6)-C(9)	105.12(12)
C(34)-Cl(5)	1.814(5)	C(7)-C(6)-C(9)	109.41(16)
N(2)-Si(1)-N(1)	72.11(5)	C(8)-C(6)-C(9)	109.75(16)
N(2)-Si(1)-C(16)	110.07(6)	C(15)-C(10)-C(11)	120.14(13)
N(1)-Si(1)-C(16)	107.09(6)	C(15)-C(10)-C(1)	120.40(13)
N(2)-Si(1)-C(1)	36.28(5)	C(11)-C(10)-C(1)	119.34(13)
N(1)-Si(1)-C(1)	36.03(5)	C(12)-C(11)-C(10)	119.75(14)
C(16)-Si(1)-C(1)	116.04(6)	C(13)-C(12)-C(11)	120.09(15)

C(14)-C(13)-C(12)	120.25(14)	C(25)-C(24)-C(23)	120.7(4)
C(13)-C(14)-C(15)	120.04(14)	C(24)-C(25)-C(26)	119.5(3)
C(10)-C(15)-C(14)	119.72(14)	C(25)-C(26)-C(27)	120.1(3)
C(17)-C(16)-C(21)	118.76(13)	C(26)-C(27)-C(22)	120.7(2)
C(17)-C(16)-Si(1)	117.42(10)	C(27A)-C(22A)-C(23A)	118.4(9)
C(21)-C(16)-Si(1)	123.23(11)	C(27A)-C(22A)-P(1)	128.9(6)
C(18)-C(17)-C(16)	121.49(13)	C(23A)-C(22A)-P(1)	112.3(8)
C(17)-C(18)-C(19)	119.29(13)	C(24A)-C(23A)-C(22A)	119.7(13)
C(20)-C(19)-C(18)	120.23(13)	C(25A)-C(24A)-C(23A)	119.6(15)
C(19)-C(20)-C(21)	120.94(13)	C(24A)-C(25A)-C(26A)	121.4(15)
C(20)-C(21)-C(16)	119.09(13)	C(25A)-C(26A)-C(27A)	119.2(12)
C(20)-C(21)-P(1)	122.58(10)	C(22A)-C(27A)-C(26A)	121.3(10)
C(16)-C(21)-P(1)	118.29(10)	C(29)-C(28)-C(33)	118.55(13)
C(28)-P(1)-C(22A)	103.33(7)	C(29)-C(28)-P(1)	125.25(12)
C(28)-P(1)-C(22)	103.33(7)	C(33)-C(28)-P(1)	116.20(11)
C(28)-P(1)-C(21)	100.44(7)	C(28)-C(29)-C(30)	120.72(15)
C(22A)-P(1)-C(21)	101.64(6)	C(31)-C(30)-C(29)	120.00(15)
C(22)-P(1)-C(21)	101.64(6)	C(30)-C(31)-C(32)	119.89(15)
C(23)-C(22)-C(27)	118.3(2)	C(33)-C(32)-C(31)	120.17(15)
C(23)-C(22)-P(1)	119.0(2)	C(32)-C(33)-C(28)	120.62(14)
C(27)-C(22)-P(1)	122.71(18)	Cl(4)-C(34)-Cl(5)	109.8(3)
C(22)-C(23)-C(24)	120.7(3)		

S1.8. Crystal structure of **8**

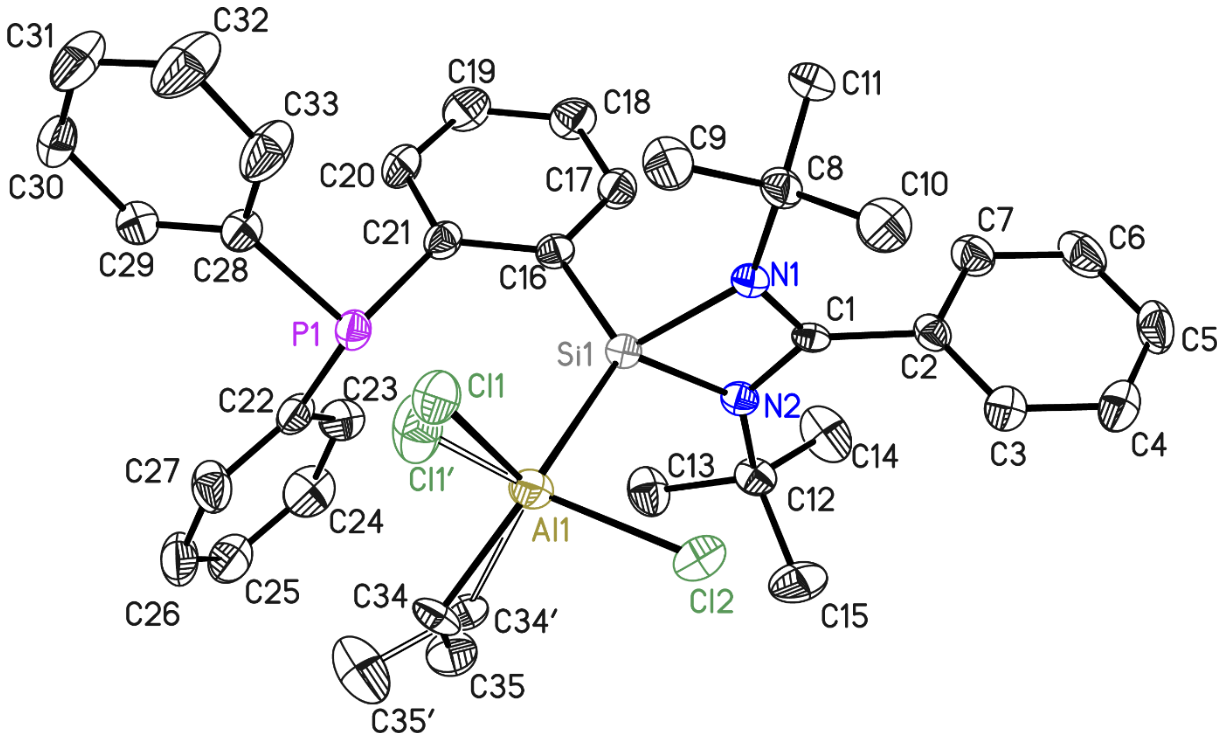

Figure 8 Molecular structure of **8** with thermal ellipsoids at 50% probability level. The hydrogen atoms are omitted for clarity.

The disordered ethyl molecule (C34, C35) and Cl1 were refined on two positions. The occupancy of the main position refined to 0.809(7). For the refinement, distance restraints and restraints for the anisotropic displacement parameters were used.

Table 9. Bond lengths [Å] and angles [°] for **8**.

P(1)-C(22)	1.825(3)
P(1)-C(28)	1.829(3)
P(1)-C(21)	1.837(4)
N(1)-C(1)	1.337(4)
N(1)-C(8)	1.483(4)
N(1)-Si(1)	1.820(3)
C(1)-N(2)	1.336(4)
C(1)-C(2)	1.487(5)
C(1)-Si(1)	2.289(3)
Cl(2)-Al(1)	2.2123(14)
N(2)-C(12)	1.492(4)
N(2)-Si(1)	1.826(3)
C(2)-C(3)	1.384(5)
C(2)-C(7)	1.398(5)
C(3)-C(4)	1.388(5)
C(4)-C(5)	1.378(5)
C(5)-C(6)	1.376(6)
C(6)-C(7)	1.384(5)
C(8)-C(10)	1.518(5)
C(8)-C(11)	1.524(5)
C(8)-C(9)	1.526(5)
C(12)-C(14)	1.507(5)
C(12)-C(13)	1.521(5)
C(12)-C(15)	1.526(5)
C(16)-C(17)	1.400(5)
C(16)-C(21)	1.402(5)
C(16)-Si(1)	1.880(3)
C(17)-C(18)	1.391(5)

C(18)-C(19)	1.388(5)	C(7)-C(2)-C(1)	120.1(3)
C(19)-C(20)	1.375(5)	C(2)-C(3)-C(4)	120.5(3)
C(20)-C(21)	1.407(5)	C(5)-C(4)-C(3)	119.9(4)
C(22)-C(23)	1.391(5)	C(6)-C(5)-C(4)	119.8(4)
C(22)-C(27)	1.395(5)	C(5)-C(6)-C(7)	121.1(4)
C(23)-C(24)	1.391(5)	C(6)-C(7)-C(2)	119.2(4)
C(24)-C(25)	1.382(6)	N(1)-C(8)-C(10)	111.4(3)
C(25)-C(26)	1.387(6)	N(1)-C(8)-C(11)	108.3(3)
C(26)-C(27)	1.381(5)	C(10)-C(8)-C(11)	111.8(3)
C(28)-C(29)	1.390(5)	N(1)-C(8)-C(9)	105.4(3)
C(28)-C(33)	1.405(5)	C(10)-C(8)-C(9)	110.4(3)
C(29)-C(30)	1.383(5)	C(11)-C(8)-C(9)	109.2(3)
C(30)-C(31)	1.376(5)	N(2)-C(12)-C(14)	111.4(3)
C(31)-C(32)	1.383(5)	N(2)-C(12)-C(13)	105.1(3)
C(32)-C(33)	1.366(6)	C(14)-C(12)-C(13)	109.3(3)
Si(1)-Al(1)	2.4988(14)	N(2)-C(12)-C(15)	109.3(3)
Al(1)-C(34)	1.952(5)	C(14)-C(12)-C(15)	111.1(3)
Al(1)-C(34')	2.000(17)	C(13)-C(12)-C(15)	110.5(3)
Al(1)-Cl(1')	2.057(14)	C(17)-C(16)-C(21)	118.2(3)
Al(1)-Cl(1)	2.215(3)	C(17)-C(16)-Si(1)	119.6(3)
C(34)-C(35)	1.474(6)	C(21)-C(16)-Si(1)	122.2(3)
C(34')-C(35')	1.41(2)	C(18)-C(17)-C(16)	121.8(3)
C(22)-P(1)-C(28)	105.78(15)	C(19)-C(18)-C(17)	119.1(3)
C(22)-P(1)-C(21)	102.69(16)	C(20)-C(19)-C(18)	120.5(3)
C(28)-P(1)-C(21)	100.22(16)	C(19)-C(20)-C(21)	120.6(3)
C(1)-N(1)-C(8)	131.9(3)	C(16)-C(21)-C(20)	119.7(3)
C(1)-N(1)-Si(1)	91.60(19)	C(16)-C(21)-P(1)	118.3(3)
C(8)-N(1)-Si(1)	136.5(2)	C(20)-C(21)-P(1)	122.0(3)
N(2)-C(1)-N(1)	105.5(3)	C(23)-C(22)-C(27)	117.8(3)
N(2)-C(1)-C(2)	126.7(3)	C(23)-C(22)-P(1)	122.4(3)
N(1)-C(1)-C(2)	127.8(3)	C(27)-C(22)-P(1)	118.8(3)
N(2)-C(1)-Si(1)	52.90(16)	C(22)-C(23)-C(24)	120.6(4)
N(1)-C(1)-Si(1)	52.66(15)	C(25)-C(24)-C(23)	120.4(4)
C(2)-C(1)-Si(1)	177.8(2)	C(24)-C(25)-C(26)	119.9(4)
C(1)-N(2)-C(12)	131.9(3)	C(27)-C(26)-C(25)	119.3(4)
C(1)-N(2)-Si(1)	91.4(2)	C(26)-C(27)-C(22)	121.9(4)
C(12)-N(2)-Si(1)	136.4(2)	C(29)-C(28)-C(33)	118.0(3)
C(3)-C(2)-C(7)	119.5(3)	C(29)-C(28)-P(1)	126.7(3)
C(3)-C(2)-C(1)	120.4(3)	C(33)-C(28)-P(1)	115.3(3)

C(30)-C(29)-C(28)	120.8(3)	C(1)-Si(1)-Al(1)	122.27(9)
C(31)-C(30)-C(29)	120.2(3)	C(34')-Al(1)-Cl(1')	114.9(7)
C(30)-C(31)-C(32)	119.7(4)	C(34)-Al(1)-Cl(2)	109.93(18)
C(33)-C(32)-C(31)	120.5(4)	C(34')-Al(1)-Cl(2)	107.4(7)
C(32)-C(33)-C(28)	120.7(4)	Cl(1')-Al(1)-Cl(2)	109.3(7)
N(1)-Si(1)-N(2)	71.43(12)	C(34)-Al(1)-Cl(1)	109.10(18)
N(1)-Si(1)-C(16)	105.92(14)	Cl(2)-Al(1)-Cl(1)	104.12(14)
N(2)-Si(1)-C(16)	104.60(13)	C(34)-Al(1)-Si(1)	123.93(15)
N(1)-Si(1)-C(1)	35.74(11)	C(34')-Al(1)-Si(1)	107.8(7)
N(2)-Si(1)-C(1)	35.70(11)	Cl(1')-Al(1)-Si(1)	119.6(6)
C(16)-Si(1)-C(1)	109.78(13)	Cl(2)-Al(1)-Si(1)	95.53(5)
N(1)-Si(1)-Al(1)	114.44(9)	Cl(1)-Al(1)-Si(1)	111.74(13)
N(2)-Si(1)-Al(1)	118.29(10)	C(35)-C(34)-Al(1)	120.7(4)
C(16)-Si(1)-Al(1)	127.90(11)	C(35')-C(34')-Al(1)	125.5(17)

S1.9. Crystal structure of **9**

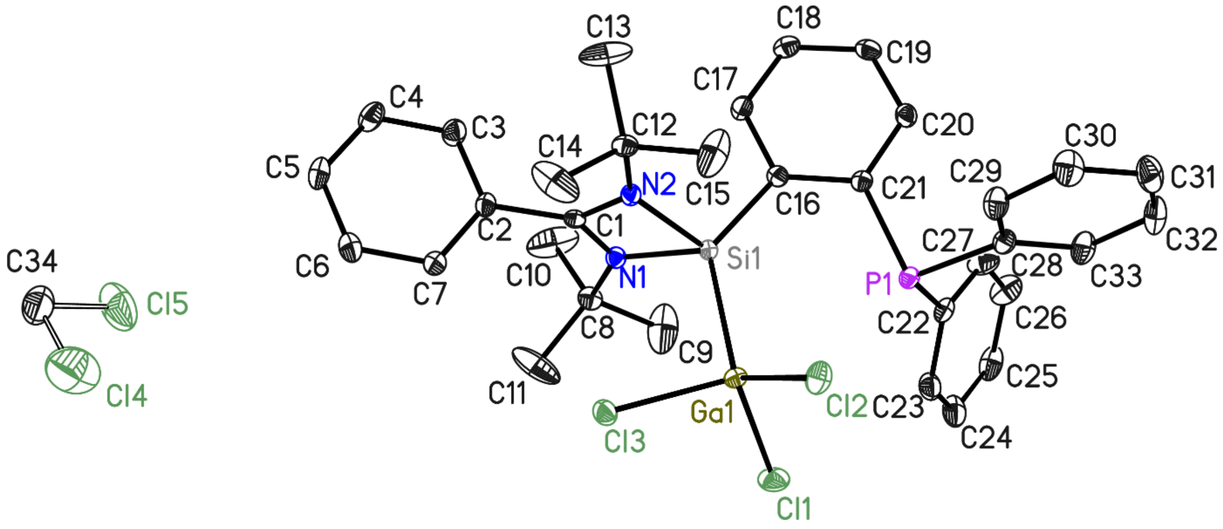

Figure 9 Molecular structure of **9** with thermal ellipsoids at 50% probability level. It consists of two fragments, one complex molecule and one DCM solvent molecule (disordered on a special position). The hydrogen atoms are omitted for clarity

For the refinement of the DCM molecule, distance restraints and restraints for the anisotropic displacement parameters were used.

Structures **7** and **9** are isomorphous.

Table 10. Bond lengths [Å] and angles [°] **9**

Cl(4)-C(34)	1.799(5)
Cl(5)-C(34)	1.735(5)
Ga(1)-Cl(2)	2.1830(5)
Ga(1)-Cl(1)	2.1917(4)
Ga(1)-Cl(3)	2.2425(5)
Ga(1)-Si(1)	2.3904(6)
P(1)-C(28)	1.8315(15)
P(1)-C(22)	1.8399(14)
P(1)-C(21)	1.8471(14)
Si(1)-N(1)	1.8053(12)
Si(1)-N(2)	1.8073(12)
Si(1)-C(16)	1.8627(14)
Si(1)-C(1)	2.2618(15)
N(1)-C(1)	1.3466(18)
N(1)-C(8)	1.4853(17)
C(1)-N(2)	1.3379(18)
C(1)-C(2)	1.4836(19)
N(2)-C(12)	1.4901(17)
C(2)-C(7)	1.390(2)
C(2)-C(3)	1.390(2)
C(3)-C(4)	1.392(2)
C(4)-C(5)	1.384(2)
C(5)-C(6)	1.386(2)
C(6)-C(7)	1.390(2)
C(8)-C(11)	1.514(2)
C(8)-C(10)	1.516(2)
C(8)-C(9)	1.519(2)
C(12)-C(13)	1.514(2)
C(12)-C(15)	1.514(2)
C(12)-C(14)	1.522(2)
C(16)-C(17)	1.4086(19)
C(16)-C(21)	1.4088(19)
C(17)-C(18)	1.384(2)
C(18)-C(19)	1.391(2)

C(19)-C(20)	1.391(2)	N(1)-C(1)-C(2)	128.68(12)
C(20)-C(21)	1.3998(19)	N(2)-C(1)-Si(1)	53.04(7)
C(22)-C(23)	1.391(2)	N(1)-C(1)-Si(1)	52.95(7)
C(22)-C(27)	1.398(2)	C(2)-C(1)-Si(1)	174.34(10)
C(23)-C(24)	1.398(2)	C(1)-N(2)-C(12)	132.38(12)
C(24)-C(25)	1.378(3)	C(1)-N(2)-Si(1)	90.70(9)
C(25)-C(26)	1.388(2)	C(12)-N(2)-Si(1)	136.63(9)
C(26)-C(27)	1.387(2)	C(7)-C(2)-C(3)	120.53(13)
C(28)-C(33)	1.392(2)	C(7)-C(2)-C(1)	119.26(12)
C(28)-C(29)	1.399(2)	C(3)-C(2)-C(1)	120.05(12)
C(29)-C(30)	1.383(2)	C(2)-C(3)-C(4)	119.38(14)
C(30)-C(31)	1.388(2)	C(5)-C(4)-C(3)	120.22(14)
C(31)-C(32)	1.381(2)	C(4)-C(5)-C(6)	120.21(14)
C(32)-C(33)	1.393(2)	C(5)-C(6)-C(7)	120.08(14)
Cl(5)-C(34)-Cl(4)	110.6(3)	C(6)-C(7)-C(2)	119.57(14)
Cl(2)-Ga(1)-Cl(1)	107.60(2)	N(1)-C(8)-C(11)	111.29(12)
Cl(2)-Ga(1)-Cl(3)	106.721(18)	N(1)-C(8)-C(10)	110.00(12)
Cl(1)-Ga(1)-Cl(3)	104.554(18)	C(11)-C(8)-C(10)	110.81(16)
Cl(2)-Ga(1)-Si(1)	117.634(18)	N(1)-C(8)-C(9)	105.22(12)
Cl(1)-Ga(1)-Si(1)	118.277(17)	C(11)-C(8)-C(9)	109.26(16)
Cl(3)-Ga(1)-Si(1)	100.31(2)	C(10)-C(8)-C(9)	110.10(16)
C(28)-P(1)-C(22)	103.65(6)	N(2)-C(12)-C(13)	109.47(12)
C(28)-P(1)-C(21)	100.43(6)	N(2)-C(12)-C(15)	105.27(11)
C(22)-P(1)-C(21)	101.39(6)	C(13)-C(12)-C(15)	110.48(15)
N(1)-Si(1)-N(2)	72.66(5)	N(2)-C(12)-C(14)	111.85(12)
N(1)-Si(1)-C(16)	112.04(6)	C(13)-C(12)-C(14)	109.97(15)
N(2)-Si(1)-C(16)	108.87(6)	C(15)-C(12)-C(14)	109.72(15)
N(1)-Si(1)-C(1)	36.54(5)	C(17)-C(16)-C(21)	119.04(12)
N(2)-Si(1)-C(1)	36.26(5)	C(17)-C(16)-Si(1)	116.30(10)
C(16)-Si(1)-C(1)	118.15(6)	C(21)-C(16)-Si(1)	124.08(10)
N(1)-Si(1)-Ga(1)	109.68(4)	C(18)-C(17)-C(16)	121.22(13)
N(2)-Si(1)-Ga(1)	107.08(4)	C(17)-C(18)-C(19)	119.38(13)
C(16)-Si(1)-Ga(1)	131.02(5)	C(20)-C(19)-C(18)	120.31(13)
C(1)-Si(1)-Ga(1)	110.58(4)	C(19)-C(20)-C(21)	120.83(13)
C(1)-N(1)-C(8)	132.18(12)	C(20)-C(21)-C(16)	119.00(12)
C(1)-N(1)-Si(1)	90.51(8)	C(20)-C(21)-P(1)	122.69(11)
C(8)-N(1)-Si(1)	136.52(9)	C(16)-C(21)-P(1)	118.27(10)
N(2)-C(1)-N(1)	105.74(11)	C(23)-C(22)-C(27)	118.37(13)
N(2)-C(1)-C(2)	125.57(12)	C(23)-C(22)-P(1)	117.66(11)

C(27)-C(22)-P(1)	123.97(11)	C(33)-C(28)-P(1)	125.39(11)
C(22)-C(23)-C(24)	120.39(15)	C(29)-C(28)-P(1)	116.05(11)
C(25)-C(24)-C(23)	120.48(15)	C(30)-C(29)-C(28)	120.71(14)
C(24)-C(25)-C(26)	119.79(14)	C(29)-C(30)-C(31)	120.11(15)
C(27)-C(26)-C(25)	119.78(15)	C(32)-C(31)-C(30)	119.90(15)
C(26)-C(27)-C(22)	121.19(15)	C(31)-C(32)-C(33)	120.06(15)
C(33)-C(28)-C(29)	118.56(13)	C(28)-C(33)-C(32)	120.61(14)

NMR Spectra for compounds **1-9**

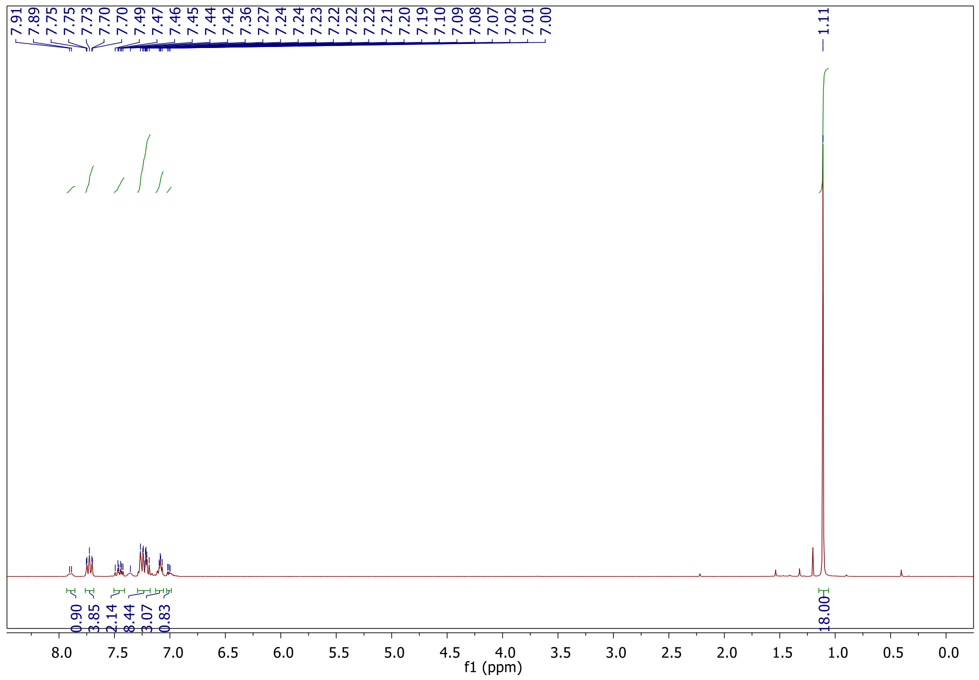

Fig. 1: ^{1}H NMR of compound **1**.

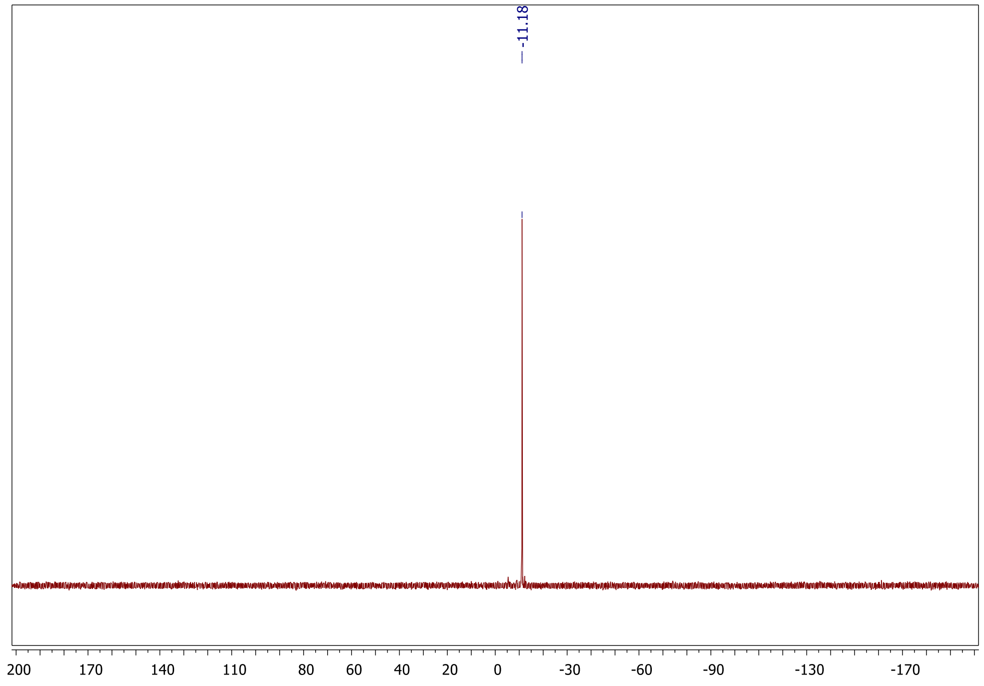

Fig. 2: ^{31}P NMR of compound **1**.

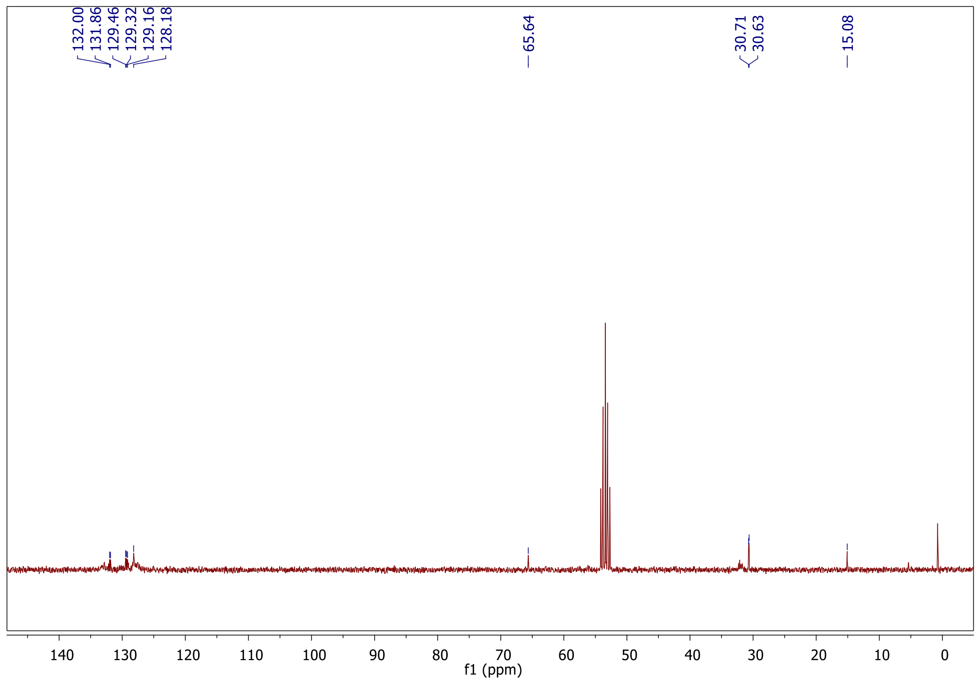

Fig. 3: ^{13}C NMR of compound **1**.

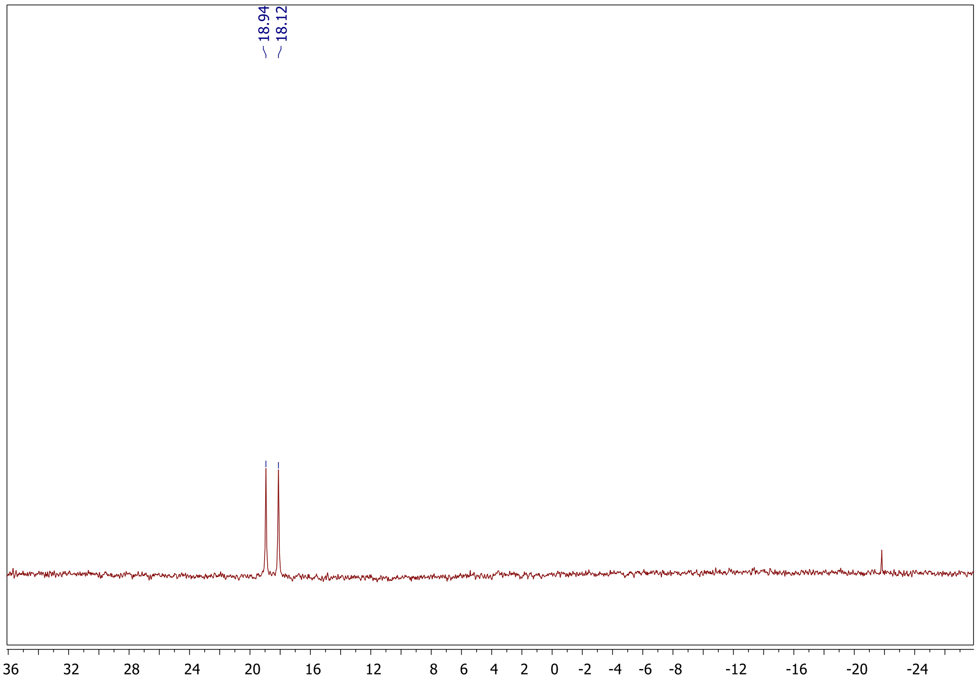

Fig. 4: ^{29}Si NMR of compound **1**.

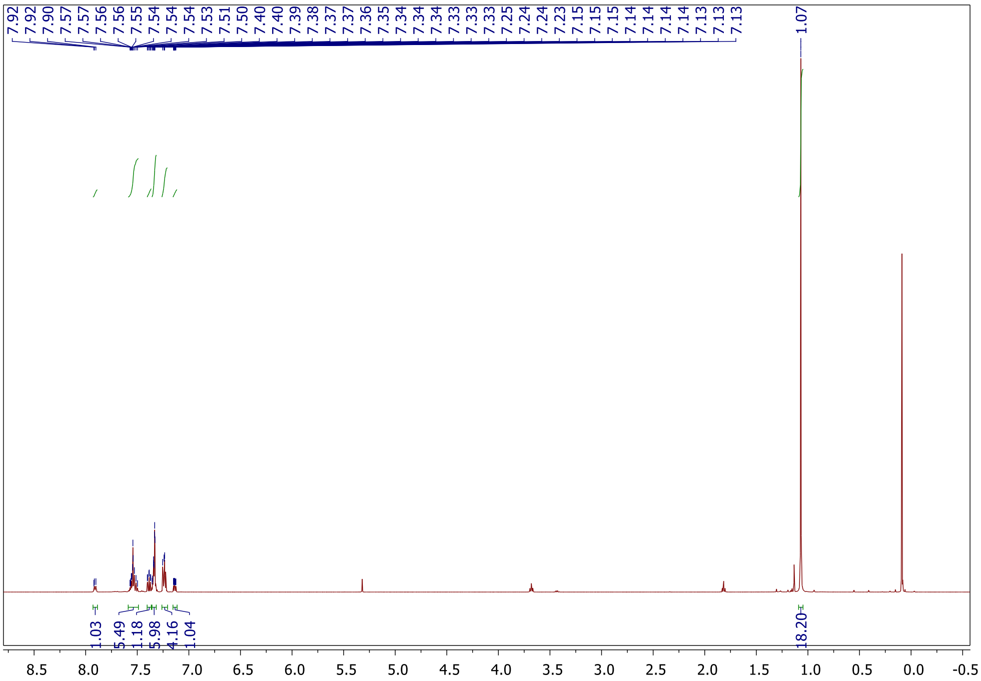

Fig. 5: ^{1}H NMR of compound **2**.

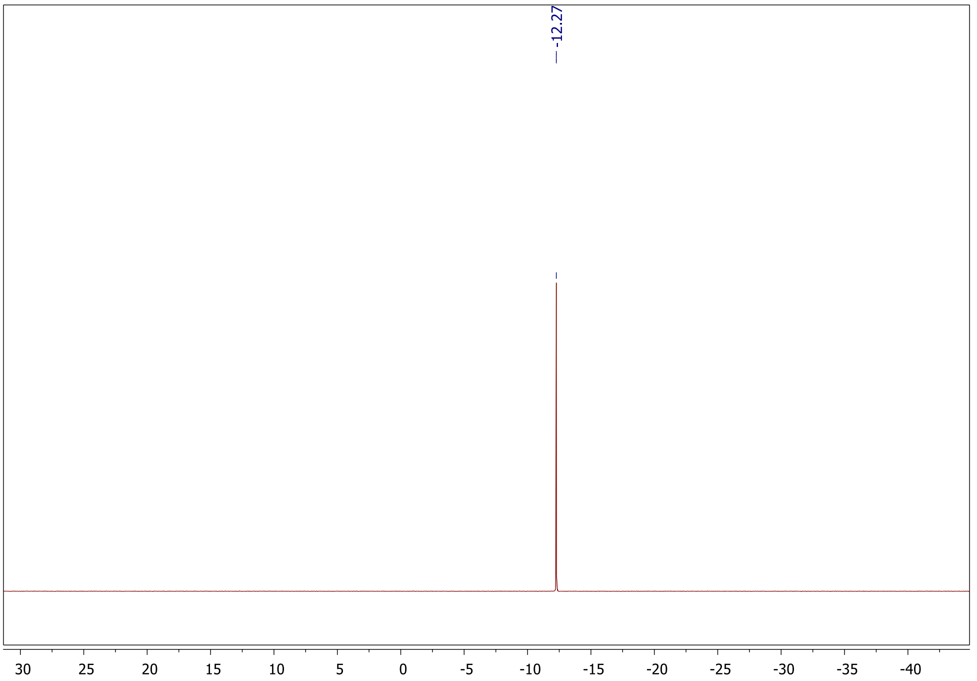

Fig. 6: ^{31}P NMR of compound **2**.

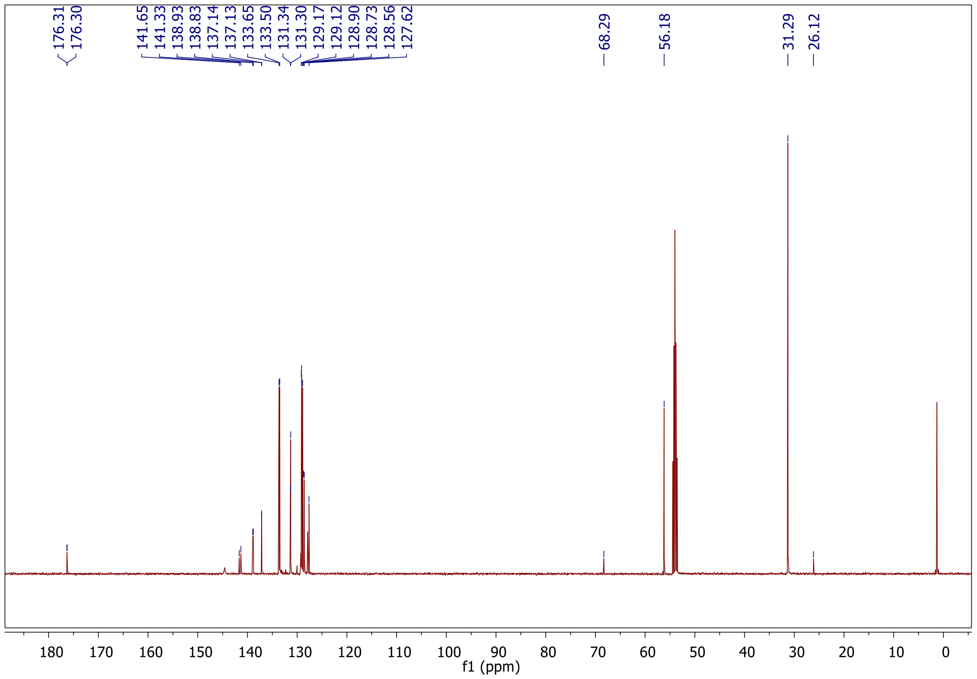

Fig. 7: ^{13}C NMR of compound **2**.

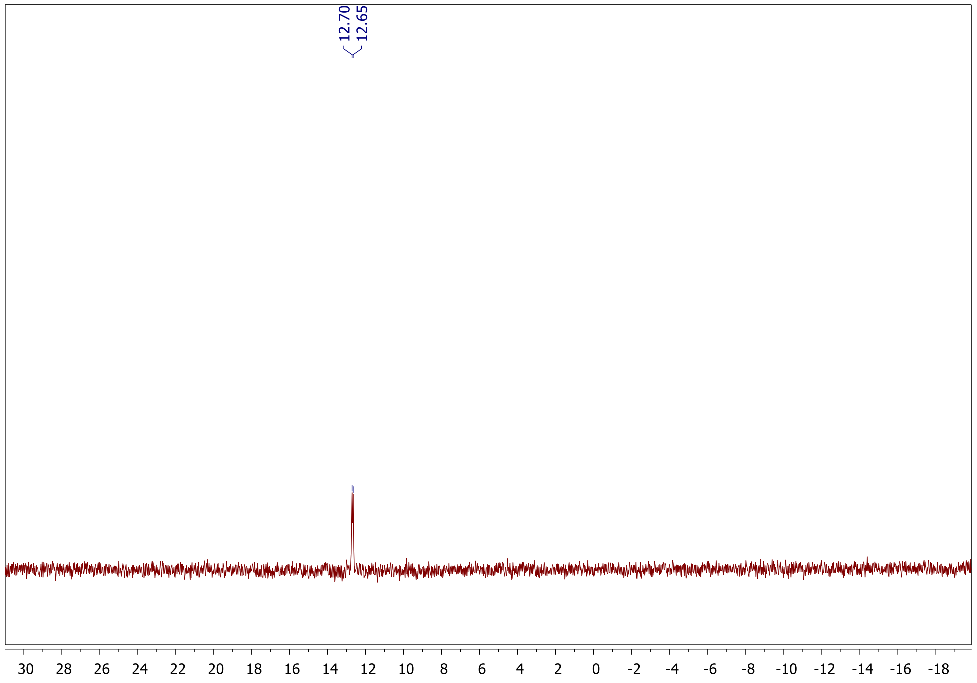

Fig. 8: ^{29}Si NMR of compound **2**.

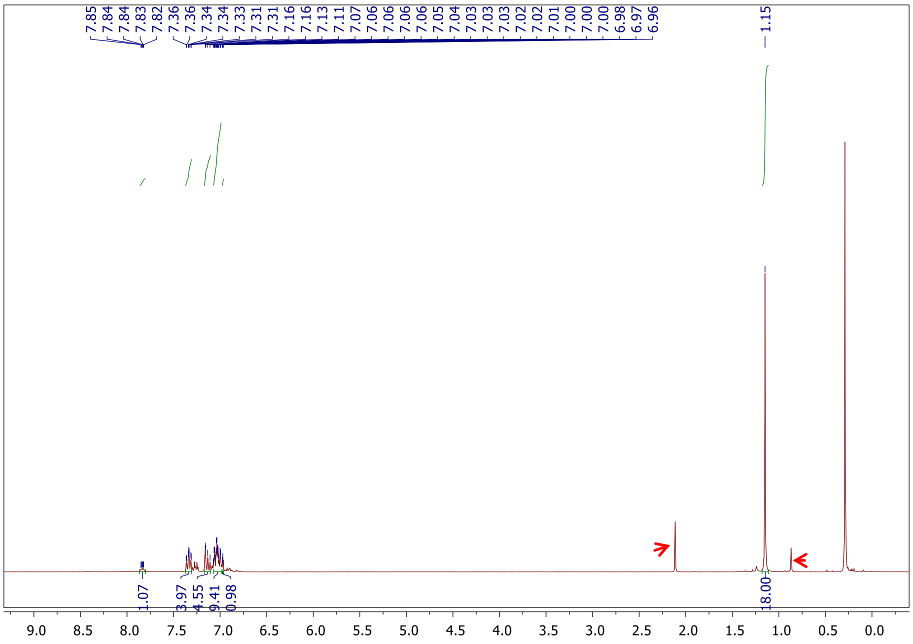

Fig. 9: ^{1}H NMR of compound **3**; 0.87 ppm (grease), 2.11 ppm (toluene) (in C_6D_6).

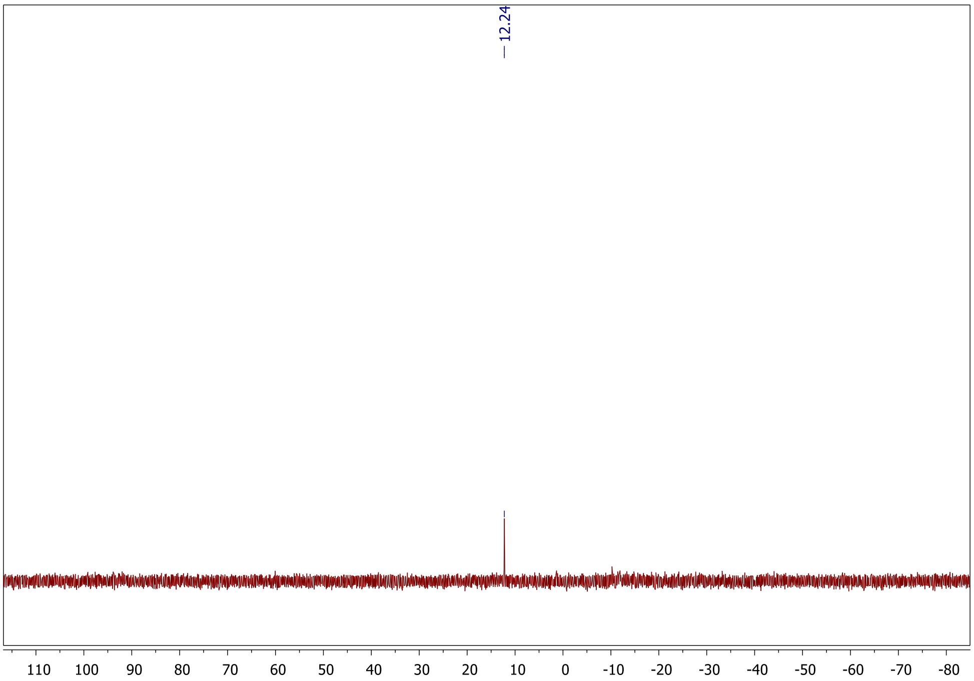

Fig. 10: ^{31}P NMR of compound **3**.

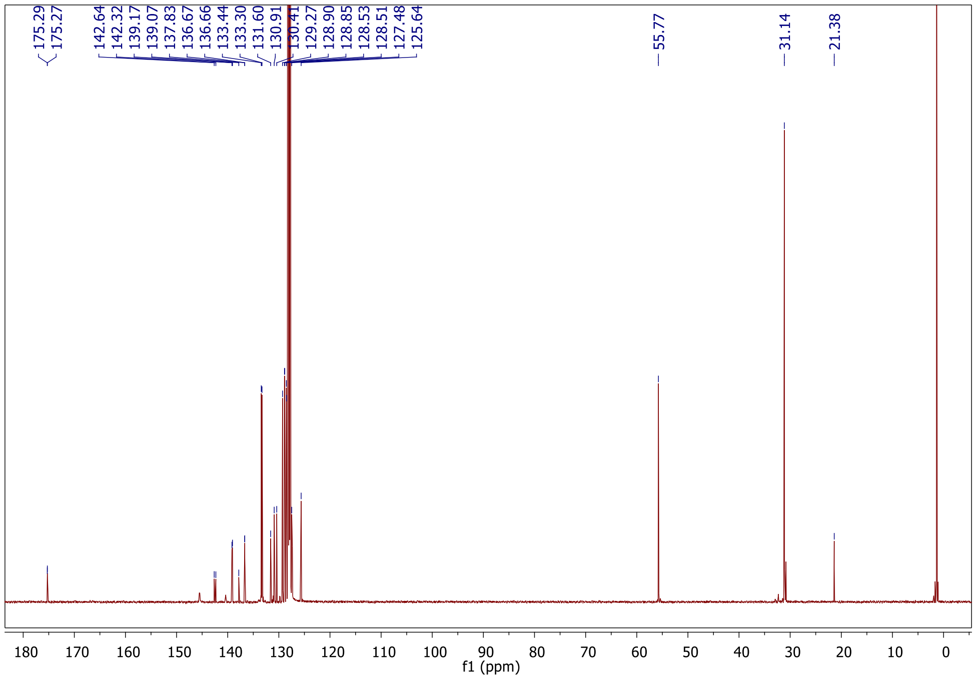

Fig. 11: ^{13}C NMR of compound **3**.

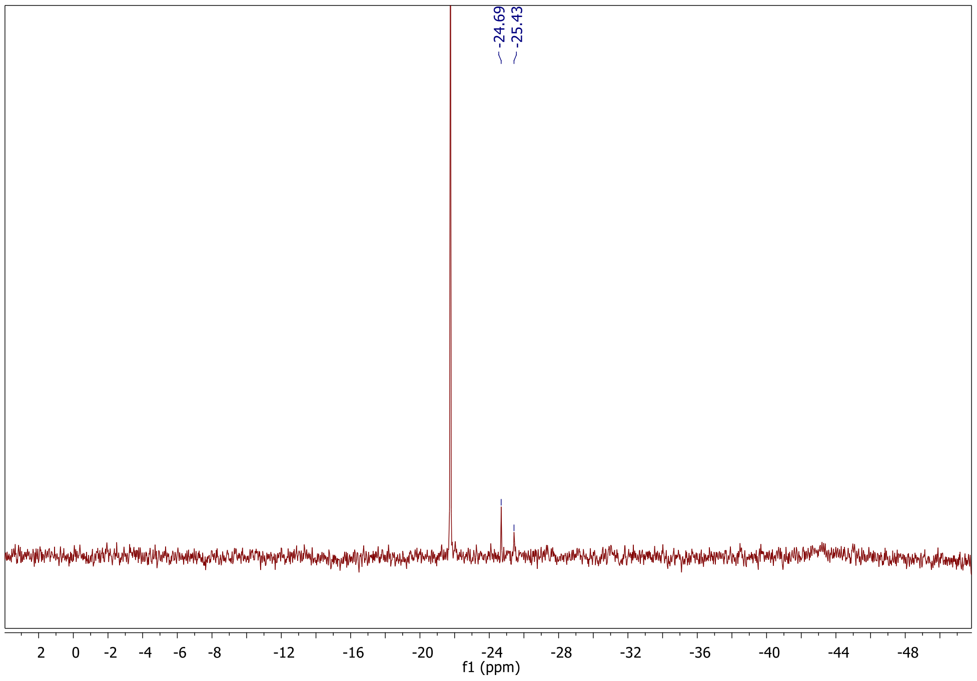

Fig. 12: ^{29}Si NMR of compound **3**.

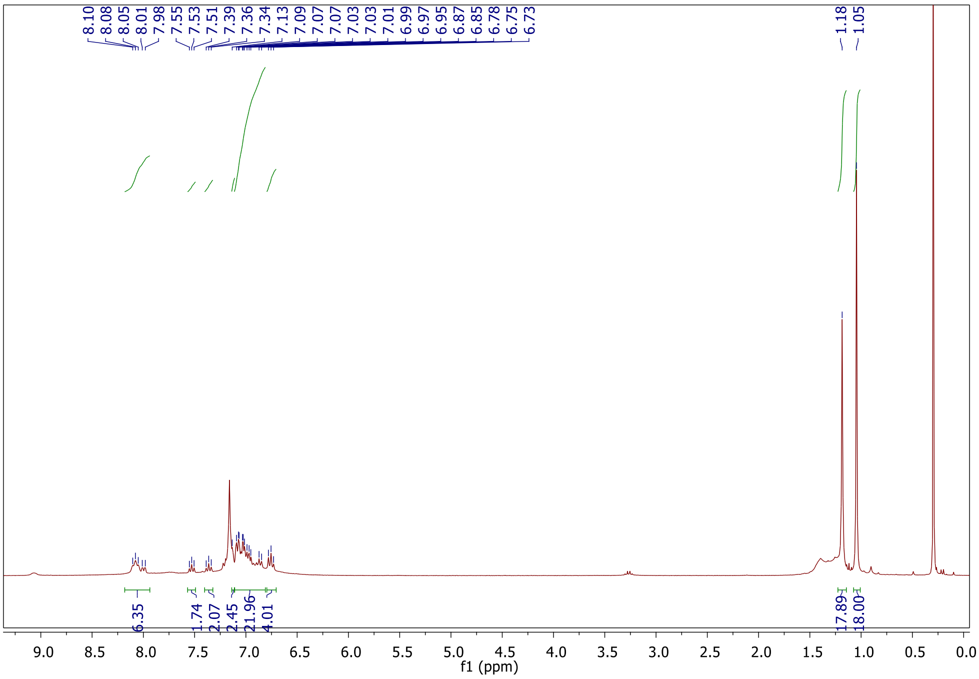

Fig. 13: ^{1}H NMR of compound **4**.

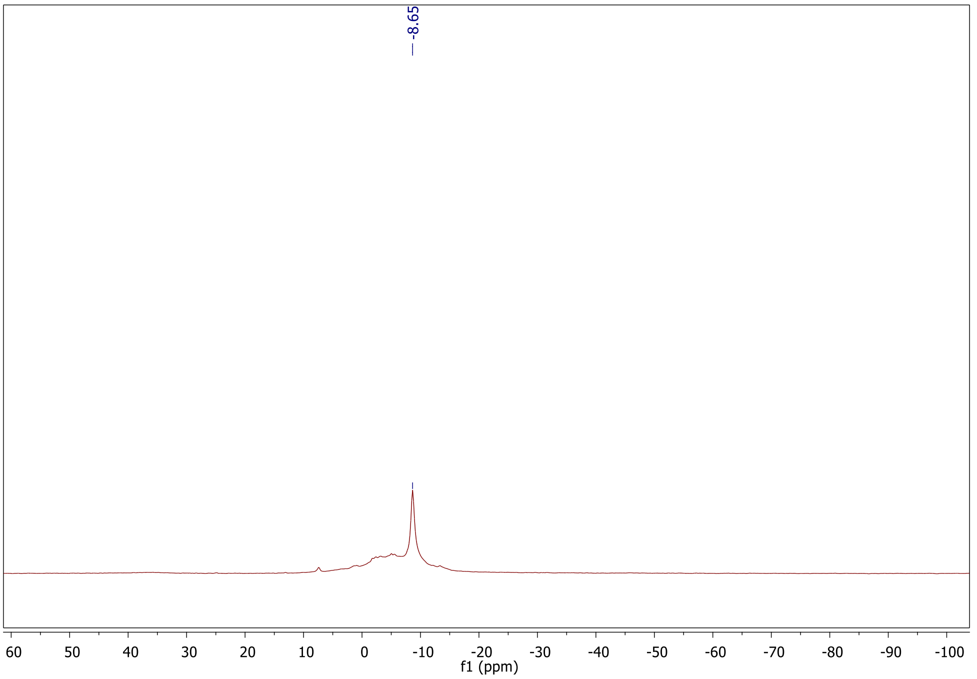

Fig. 14: ^{31}P NMR of compound **4**.

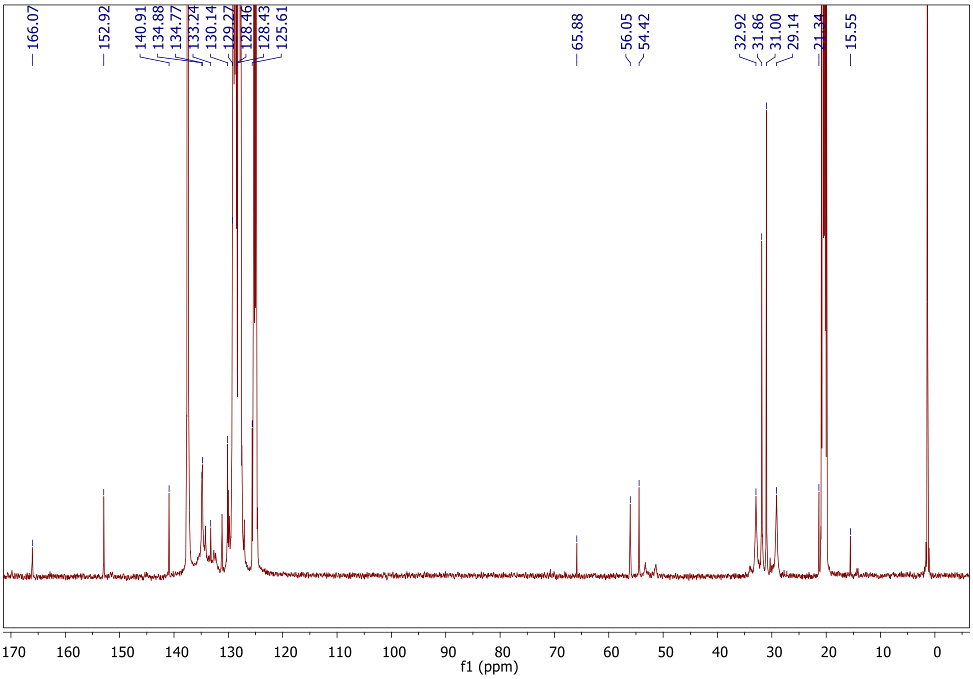

Fig. 15: ^{13}C NMR of compound **4**.

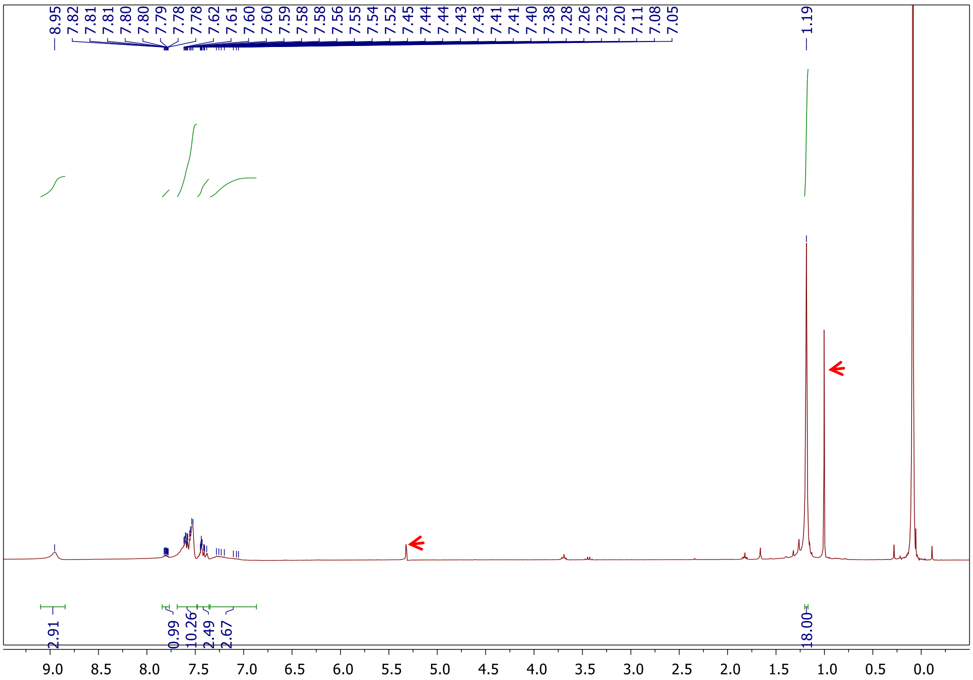

Fig. 16: ^{1}H NMR of compound **5**; 1.00 ppm (grease), 5.32 ppm (CD_2Cl_2).

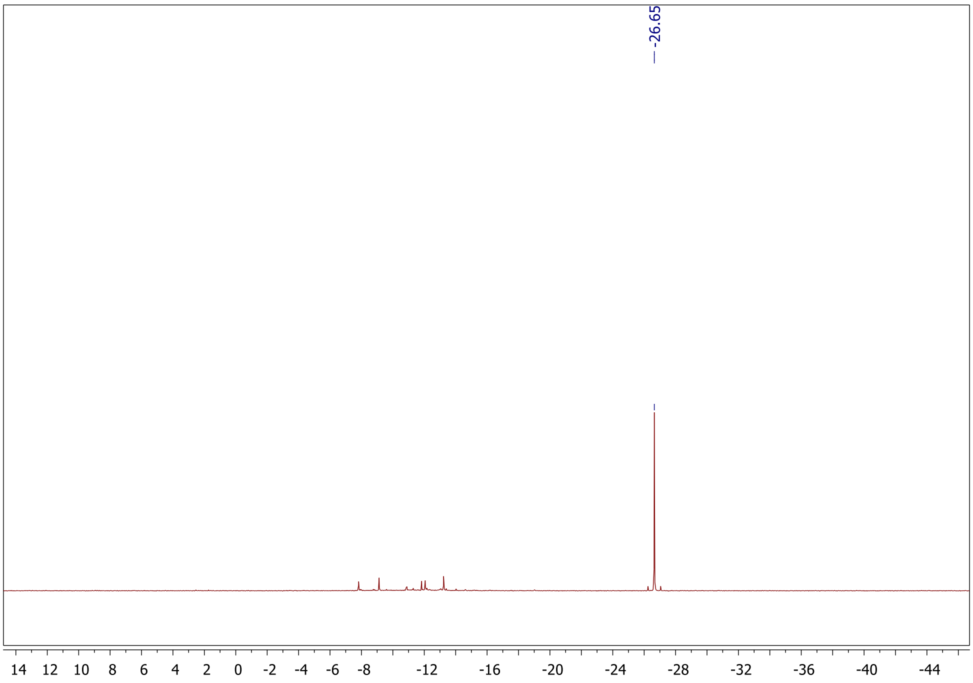

Fig. 17: ^{31}P NMR of compound **5**.

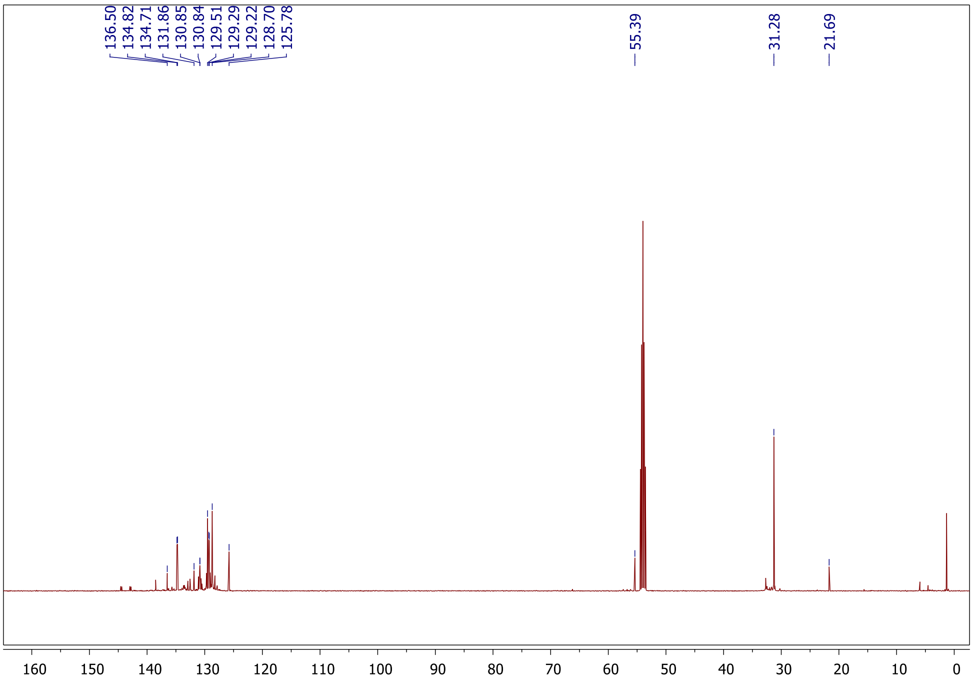

Fig. 18: ^{13}C NMR of compound **5**.

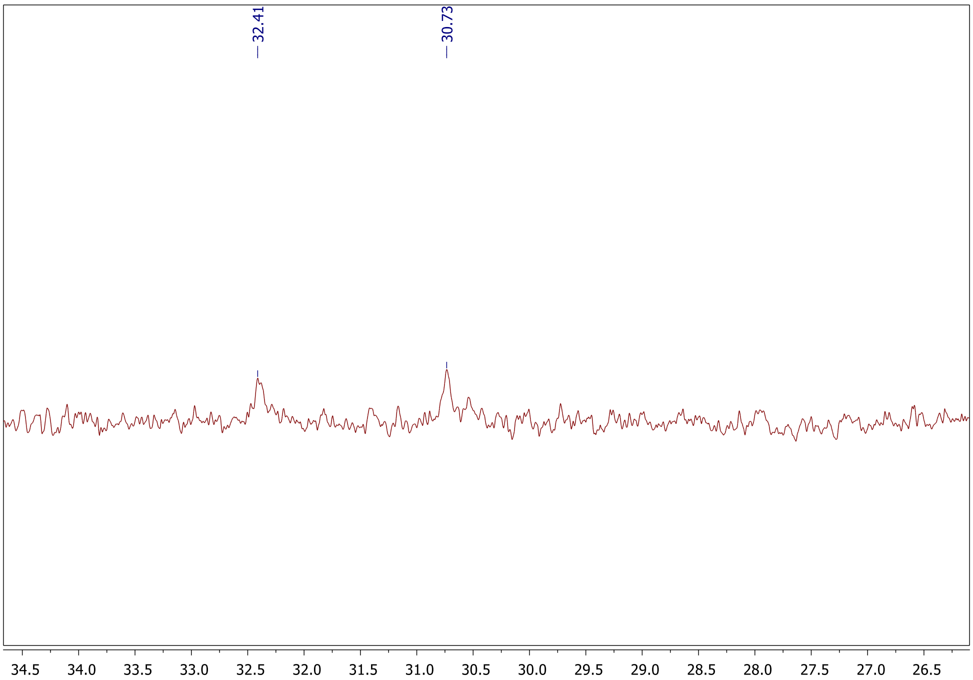

Fig. 19: ^{29}Si NMR of compound **5**.

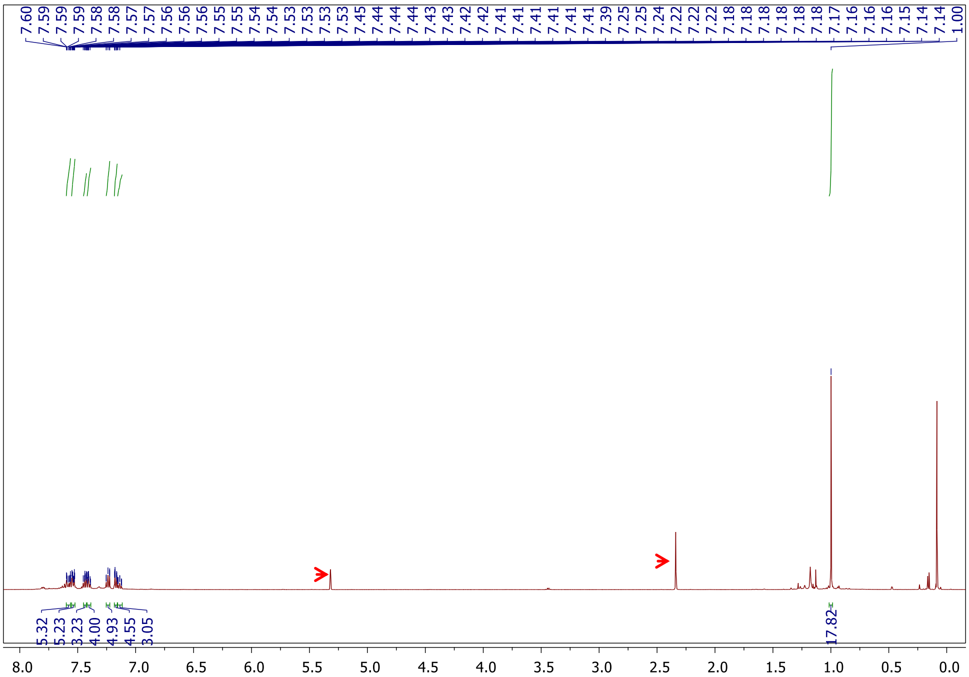

Fig. 20: ^{1}H NMR of compound **6**; 2.34 ppm (toluene), 5.32 ppm (CD_2Cl_2).

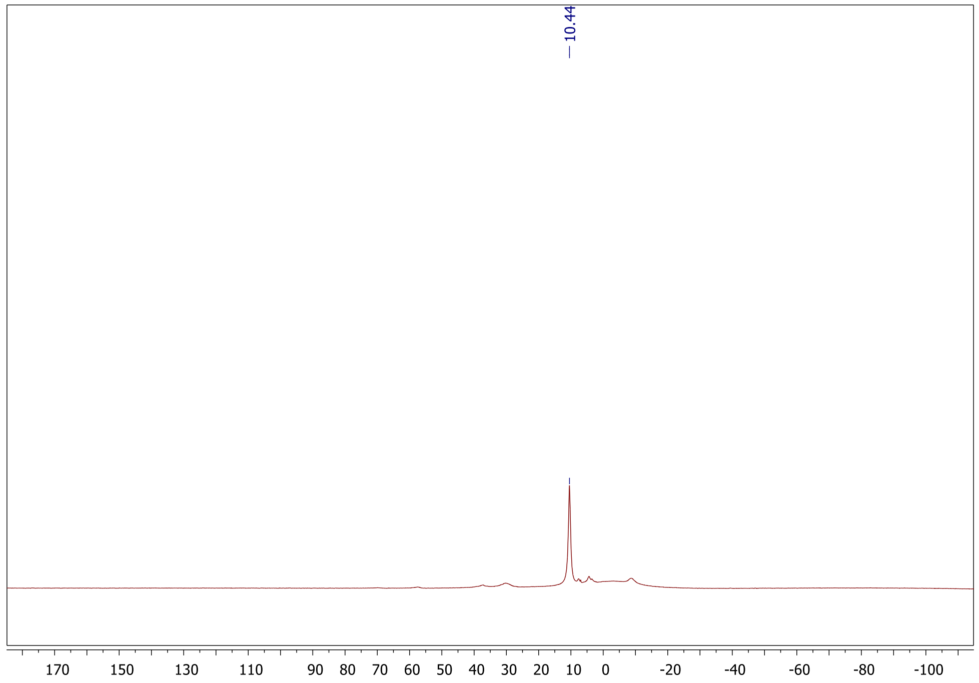

Fig. 21: ^{11}B NMR of compound **6**.

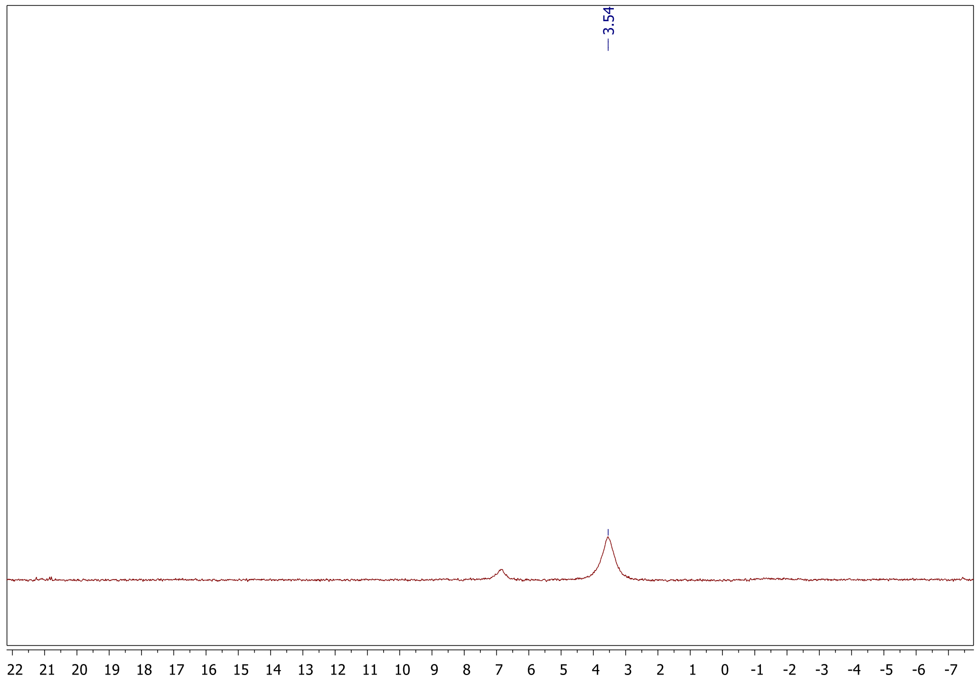

Fig. 22: ^{31}P NMR of compound **6**.

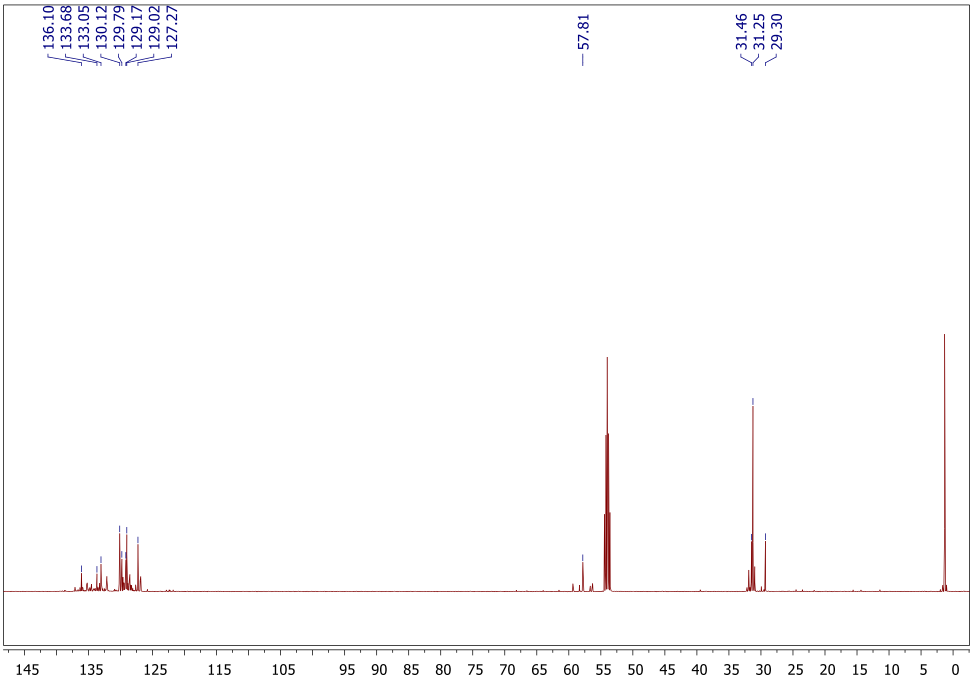

Fig. 23: ^{13}C NMR of compound **6**.

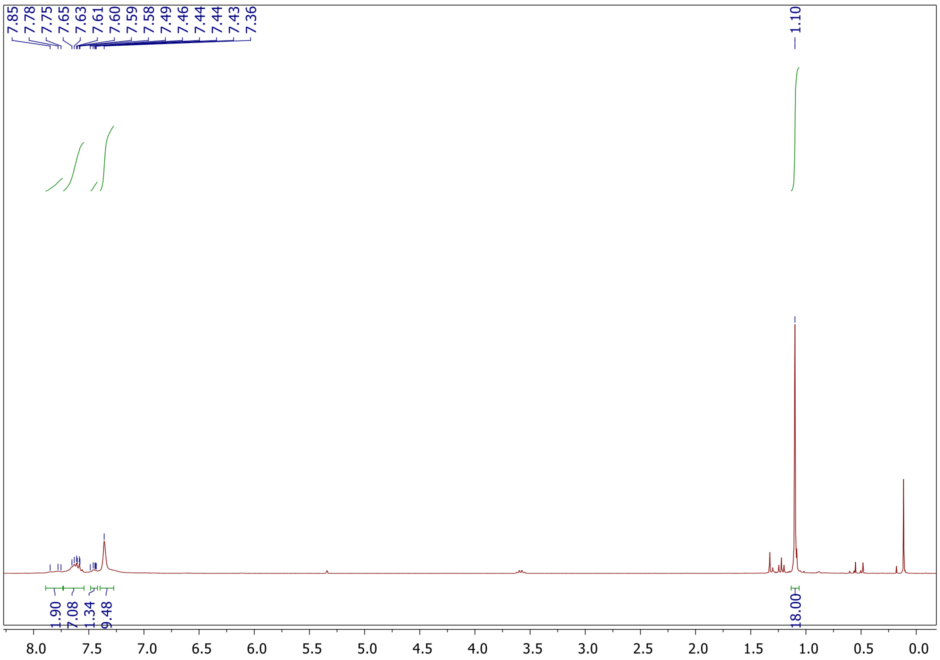

Fig. 24: ^{1}H NMR of compound **7**.

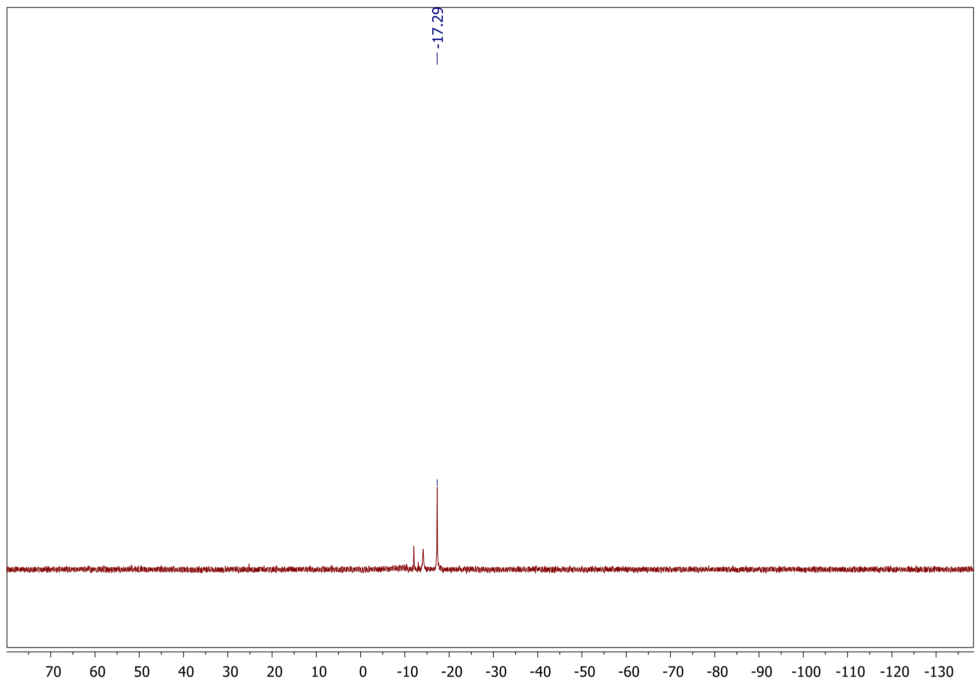

Fig. 25: ^{31}P NMR of compound **7**.

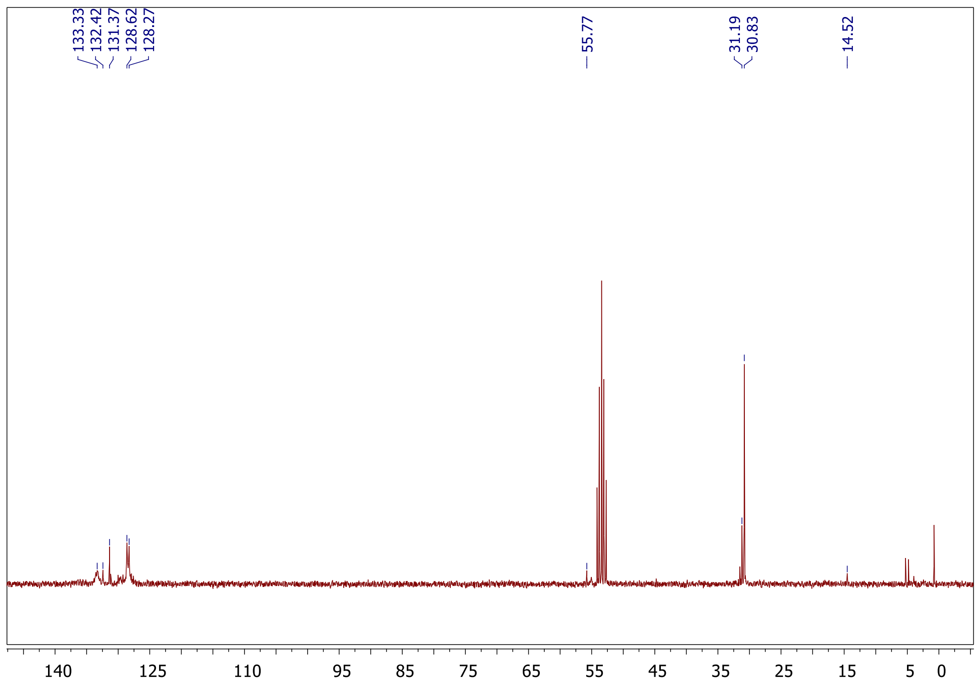

Fig. 26: ^{13}C NMR of compound **7**.

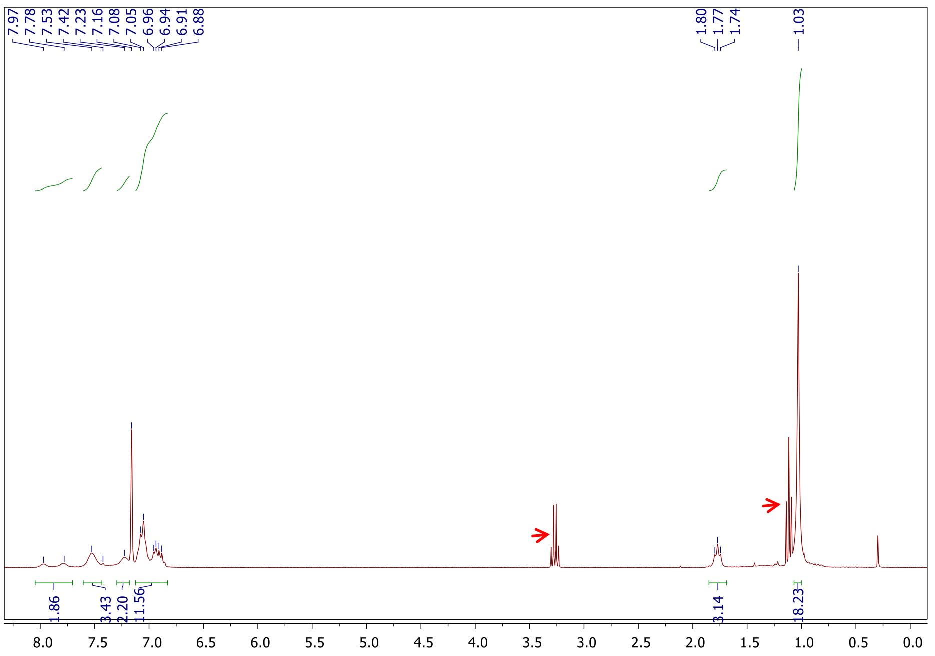

Fig. 27: ^{1}H NMR of compound **8**; 1.12 and 3.70 ppm (diethyl Ether).

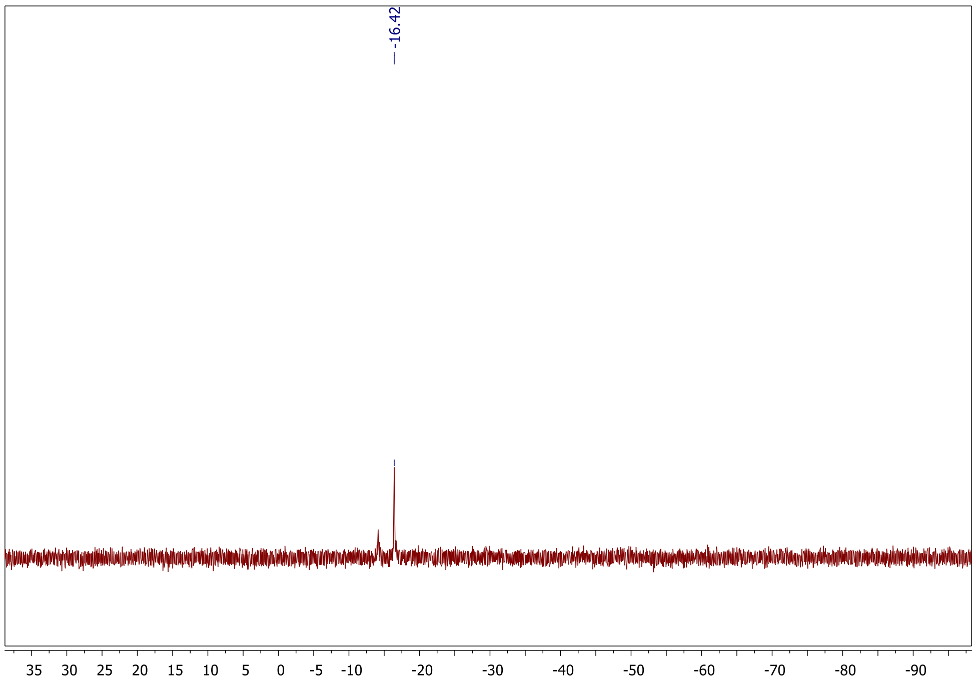

Fig. 28: ^{31}P NMR of compound **8**.

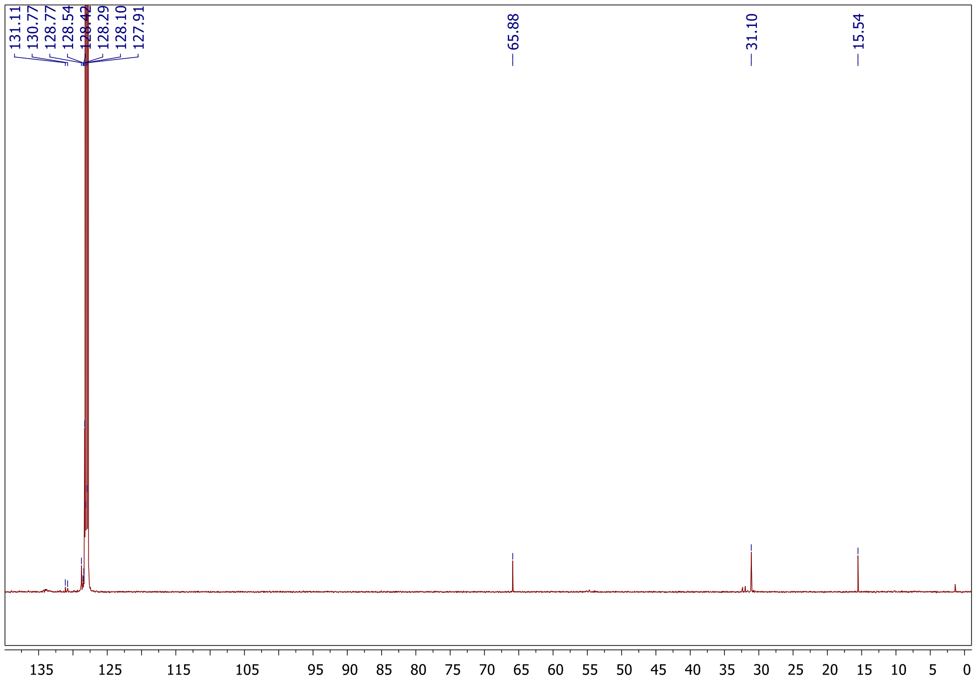

Fig. 29: ^{13}C NMR of compound **8**.

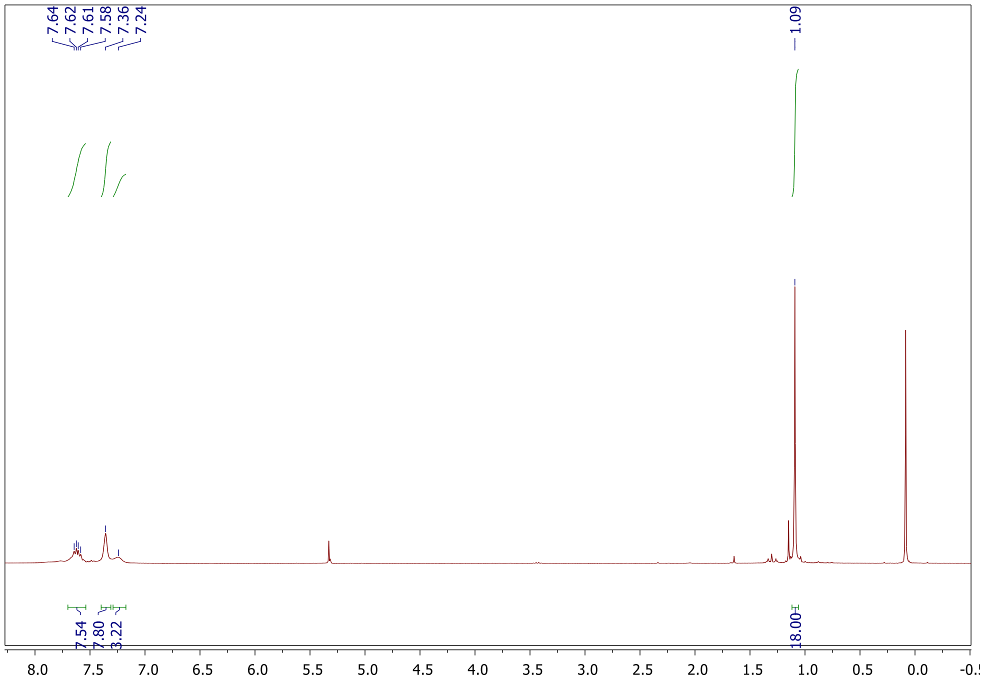

Fig. 30: ^{1}H NMR of compound **9**;

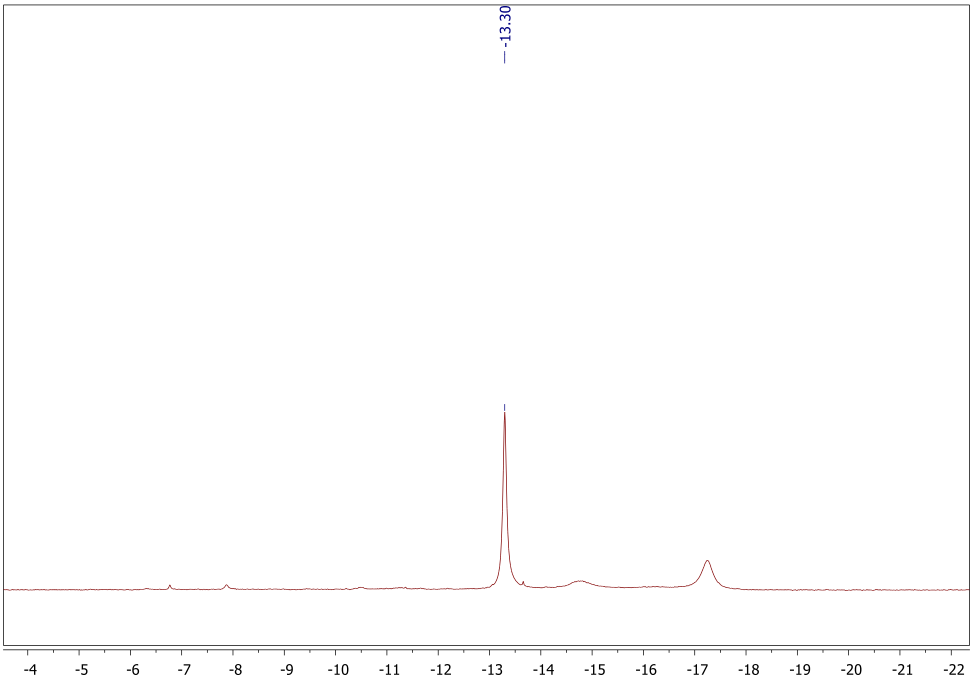

Fig. 31: ^{31}P NMR of compound **9**.

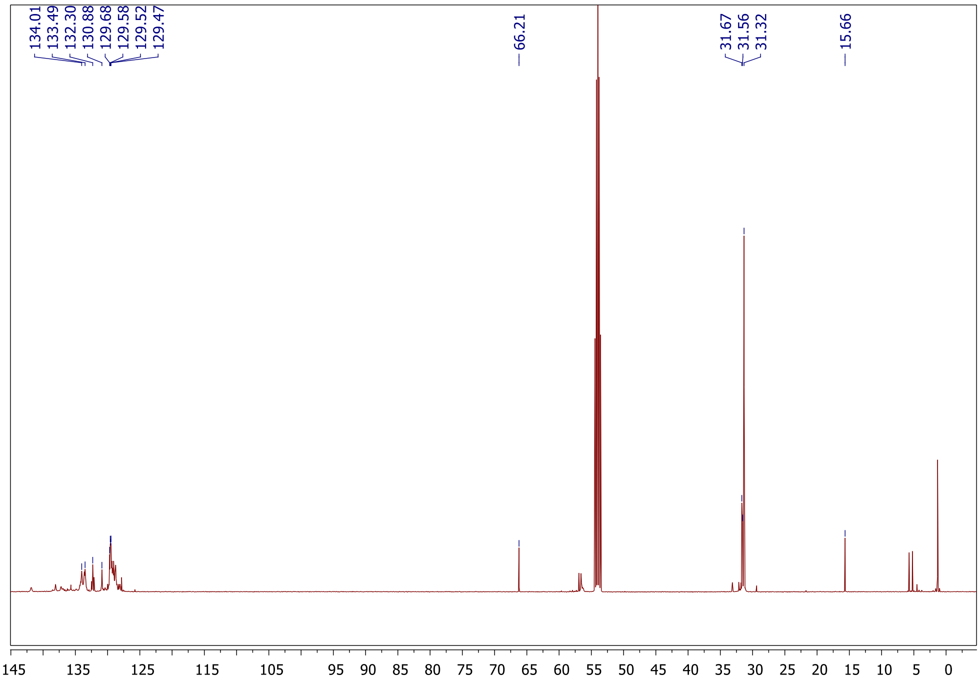

Fig. 32: ^{13}C NMR of compound **9**.

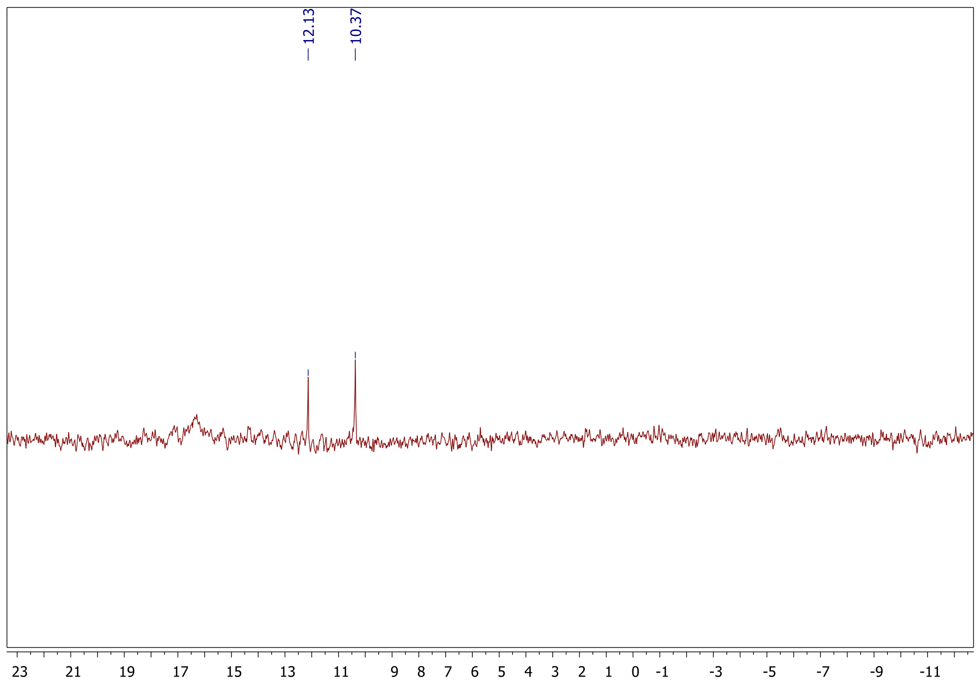

Fig. 33: ^{29}Si NMR of compound **9**.

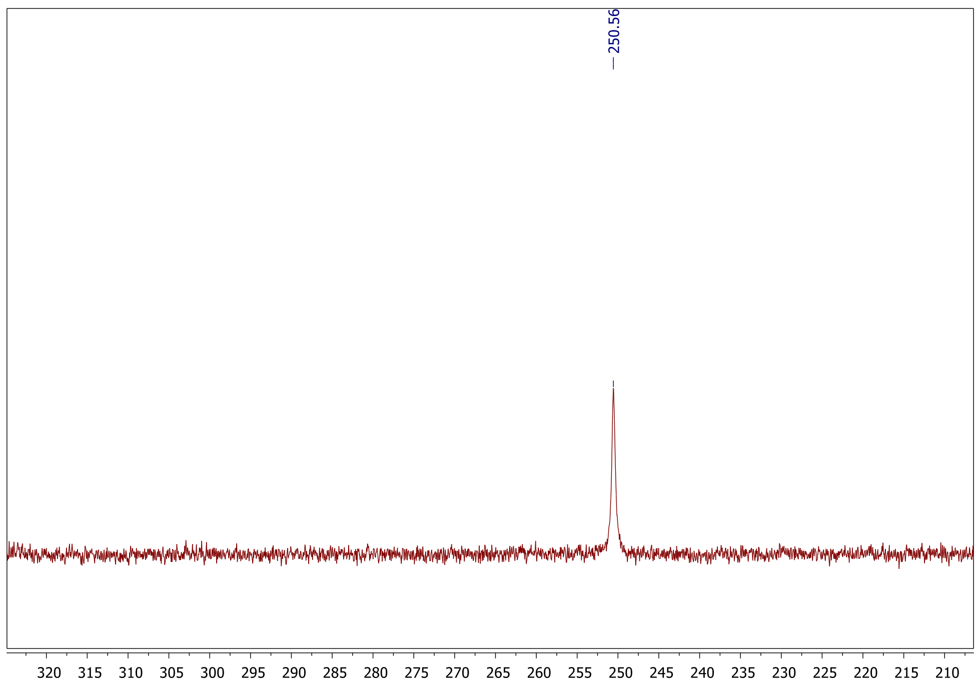

Fig. 34: ^{71}Ga NMR of compound **9**.

Mass Spectra for compounds **1-3** and **5-9**

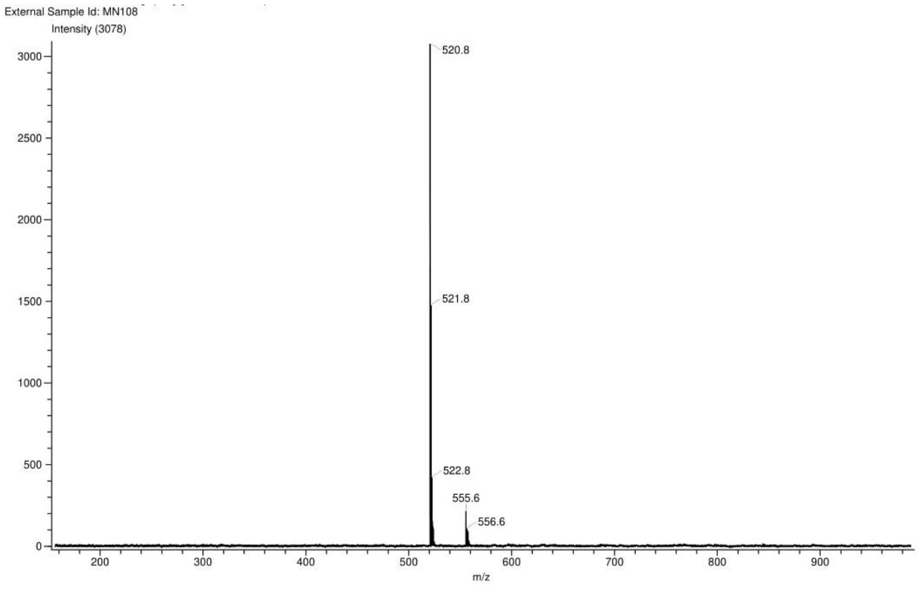

Fig. 1: Compound **1**; MS (LIFDI, toluene): *m/z* = 520 ([M]$^+$)

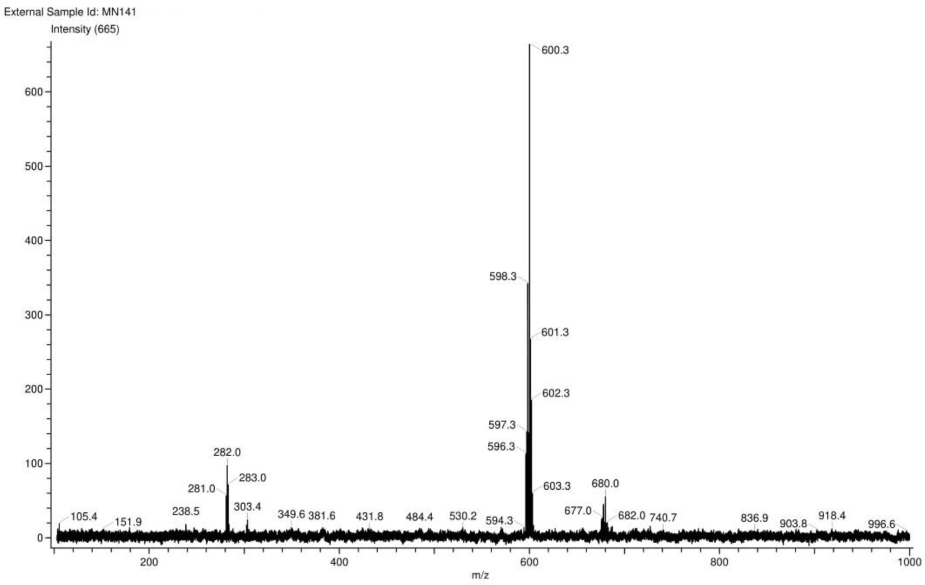

Fig. 2: Compound **2**; MS (LIFDI, toluene): *m/z* = 600 ([M]$^+$)

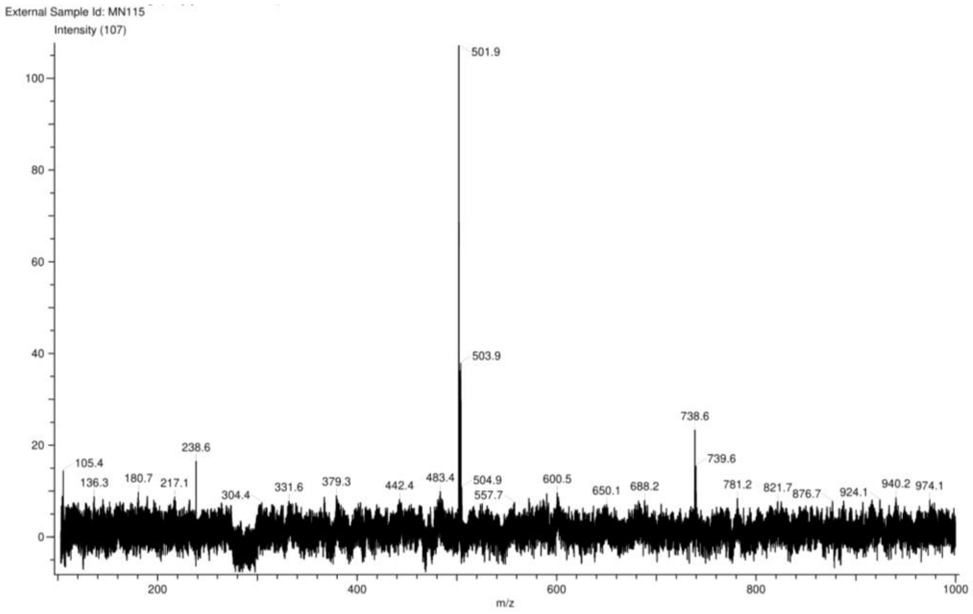

Fig. 3: Compound **3**; MS (LIFDI, toluene): *m/z* = 501 ([M- ^{t}BuCl+H_3]$^+$)

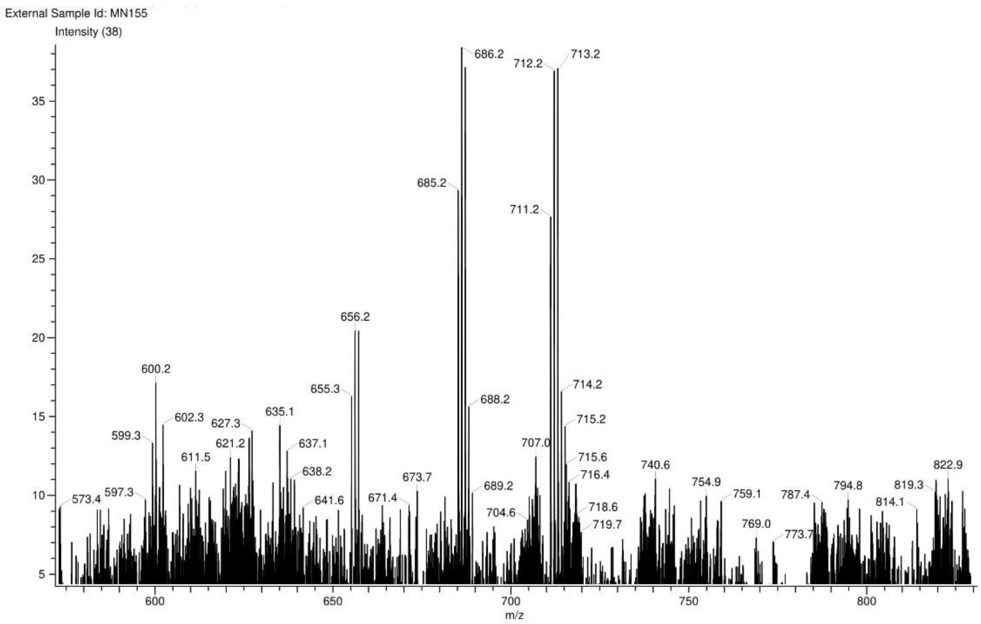

Fig. 4: Compound **5**; MS (LIFDI, toluene): *m/z* = 654 ([M+H]$^+$)

Under MS condition no reliable spectrum was obtained, due to the fragmentation of compound **5.**

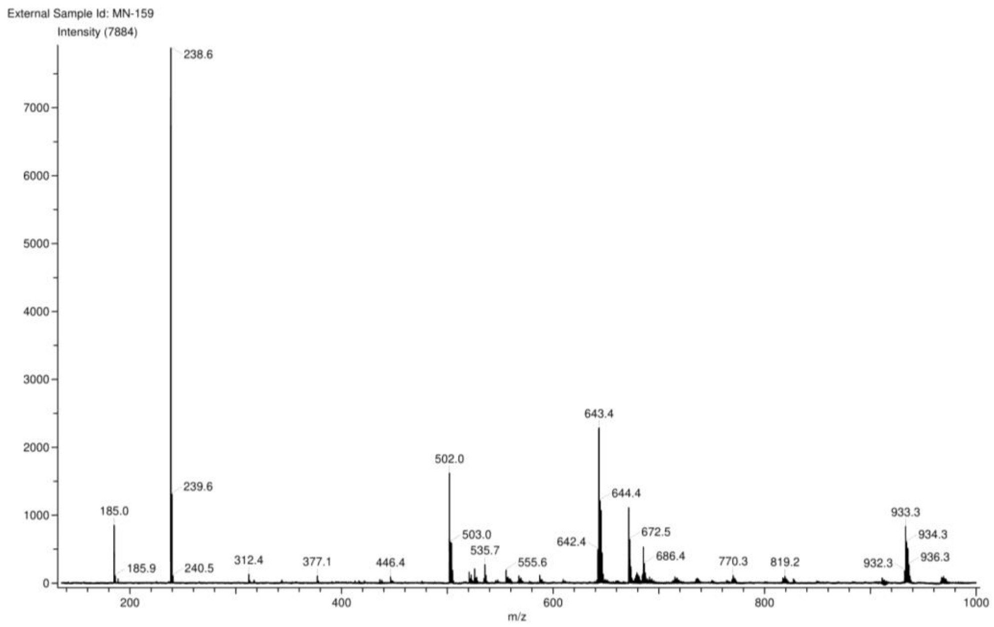

Fig. 5: Compound **6**; MS (LIFDI, toluene): *m/z* = 643 ([M-PhBCl$_3$]$^+$)

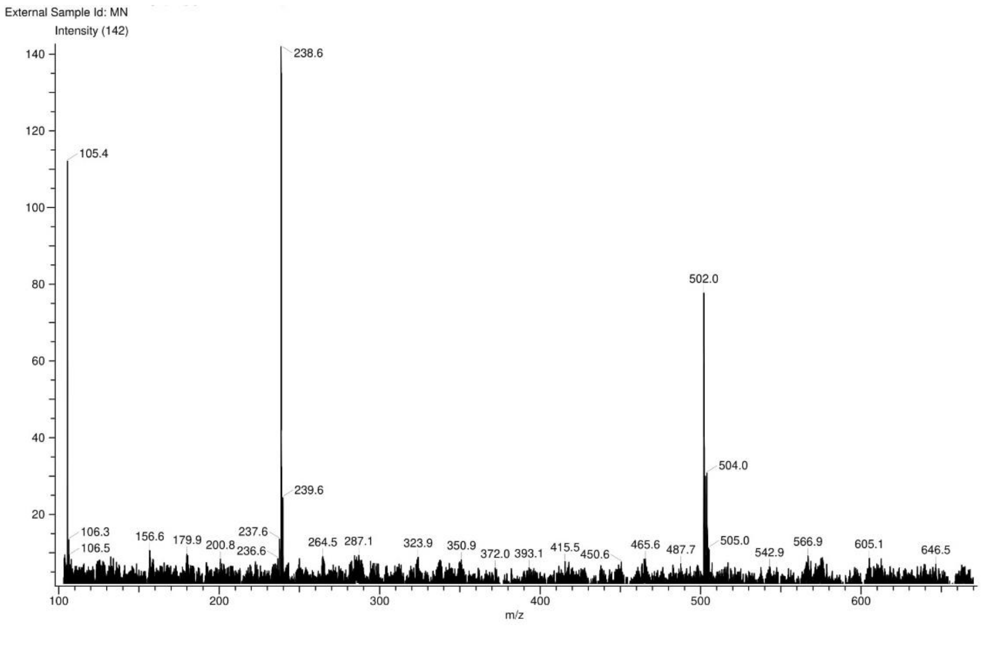

Fig. 6: Compound **7**; MS (LIFDI, toluene): *m/z* = 607 ([M-Cl$_2$+Na+H$_2$]$^+$)

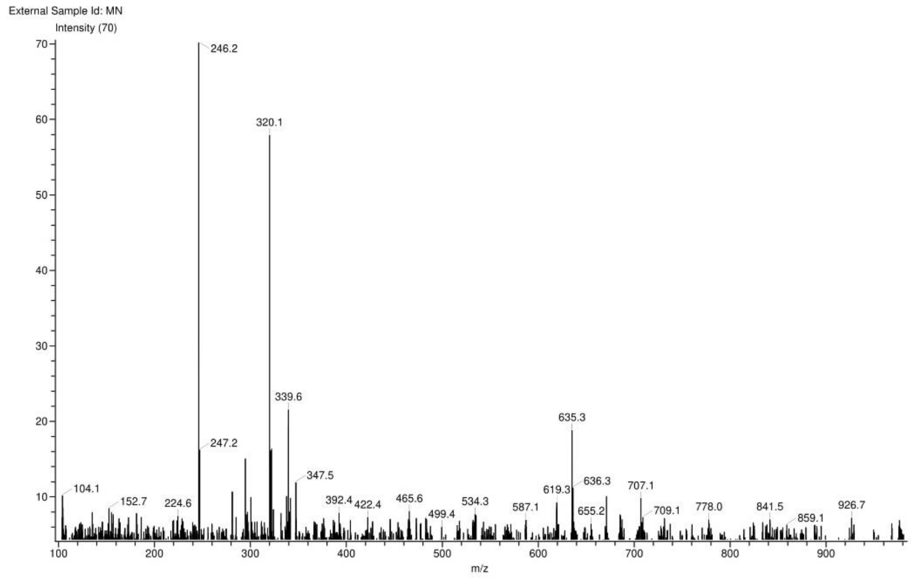

Fig. 7: Compound **8**; MS (LIFDI, toluene): *m/z* = 635 ([M-Cl+Na]$^+$)

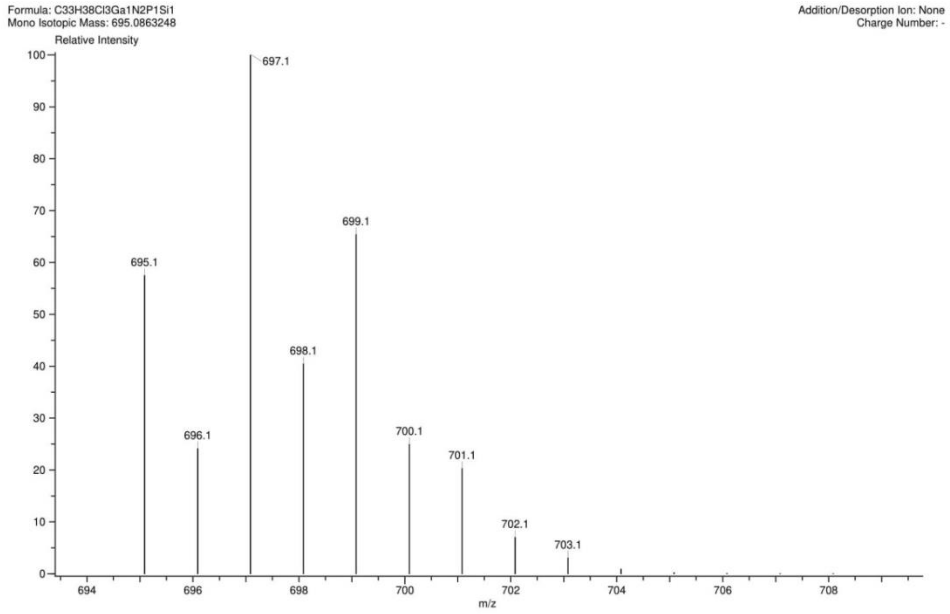

Fig. 8: Compound **9**; MS (LIFDI, toluene): *m/z* = 694 ([M+H]$^+$)

Bond length comparisons for compound **4**

We propose the following bond length histograms to help understanding the structure of compound **4**. These histograms were obtained from Conquest/Mercury after analysis of the data currently available on the CCDC database (up to June 2020).

The areas corresponding to comparable bond lengths that were found in **4** are highlighted in red. Other areas are marked in blue.

All histograms feature the amount of structures found in the database vs the corresponding bond length of a Cu-X bond (X=Si, P, Cl) for different copper oxidation states.

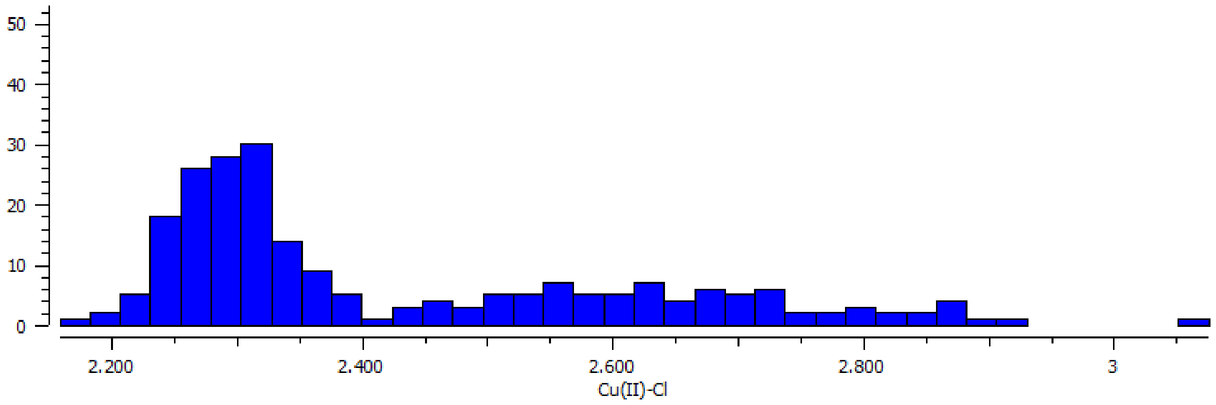

Fig. 1: Histogram of reported Cu(II)-Cl bond lengths

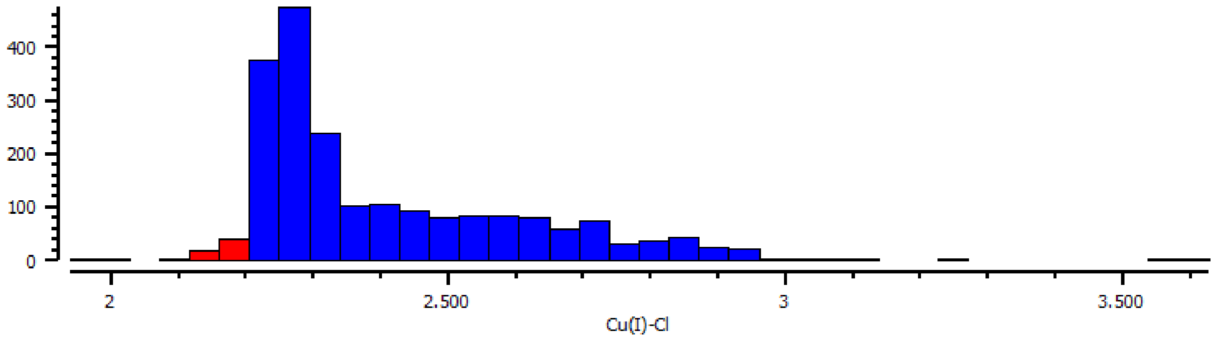

Fig. 2: Histogram of reported Cu(I)-Cl bond lengths

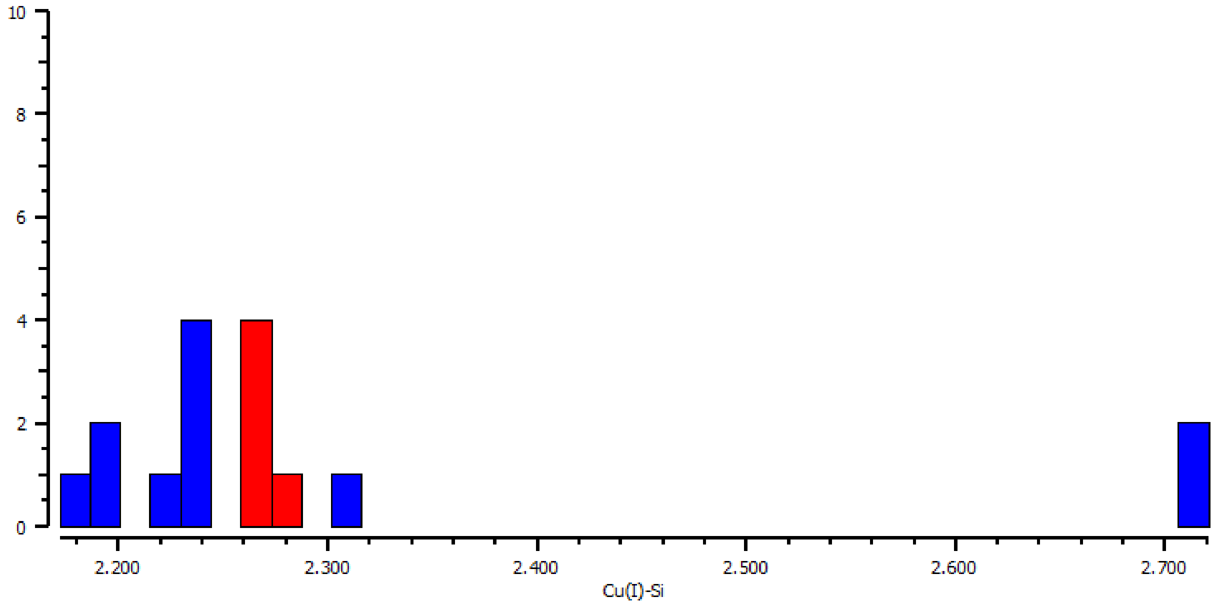

Fig. 3: Histogram of reported Cu(I)-Si bond lengths

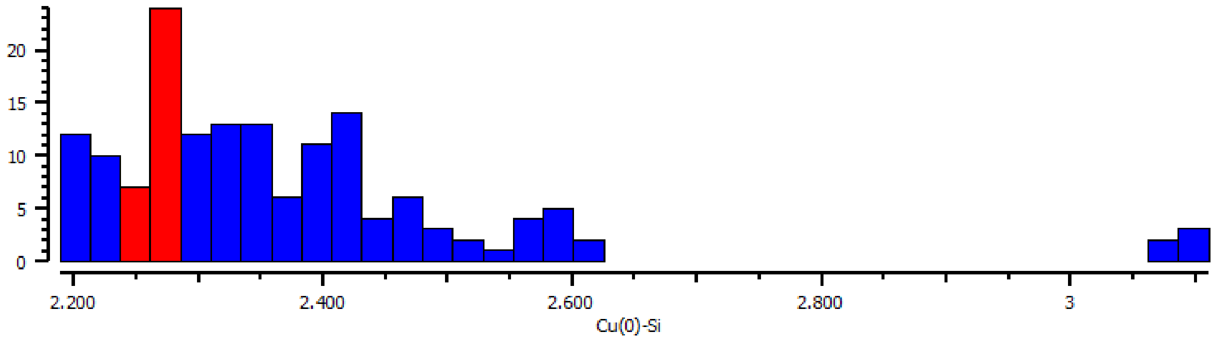

Fig. 4: Histogram of reported Cu(0)-Si bond lengths

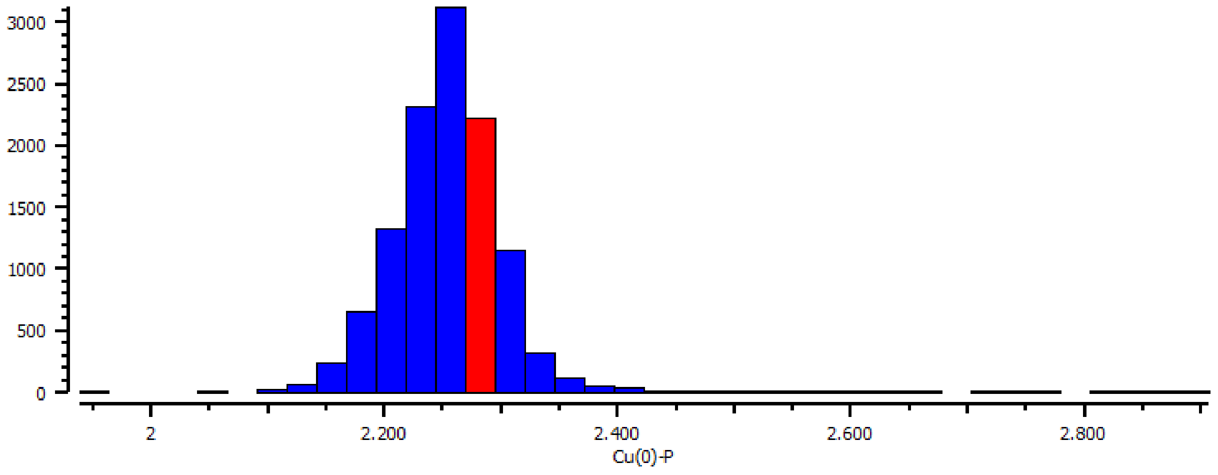

Fig. 5: Histogram of reported Cu(0)-P bond lengths

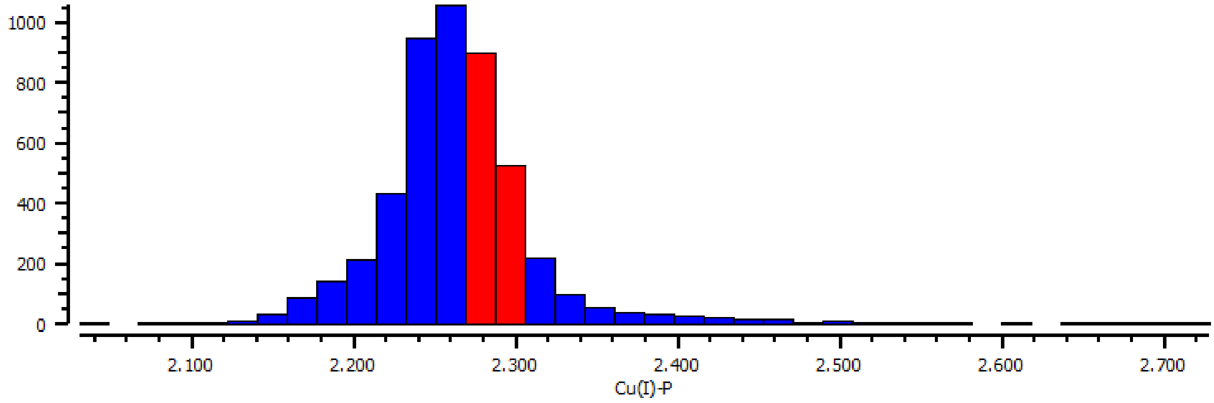

Fig. 7: Histogram of reported Cu(I)-P bond lengths

References

[S1] T. Schulz, K. Meindl, D. Leusser, D. Stern, J. Graf, C. Michaelsen, M. Ruf, G. M. Sheldrick, D. Stalke, *J. Appl. Crystallogr.* **2009**, *42*, 885-891.

[S2] Bruker AXS Inc., *SAINT, Madison*, **2016**.

[S3] L. Krause, R. Herbst-Irmer, D. Stalke, *J. Appl. Crystallogr.*, **2015**, *48*, 1907-1913.

[S4] L. Krause, R. Herbst-Irmer, G. M. Sheldrick, D. Stalke, *J. Appl. Crystallogr.* **2015**, *48*, 3-10.

[S5] M. Sevvana, M. Ruf, I. Usón, G. M. Sheldrick, R. Herbst-Irmer, *Acta Crystallogr.*, **2019**, *D75*, 1040-1050.

[S6] G. M. Sheldrick, *Acta Crystallogr.* **2015**, *A71*, 3-8.

[S7] G. M. Sheldrick, *Acta Crystallogr.* **2015**, *C71*, 3-8.

[S8] C. B. Hübschle, G. M. Sheldrick, B. Dittrich, *J. Appl. Cryst.*, **2011**, *44*, 1281-1284.

[S9] A. Thorn, B. Dittrich, G. M. Sheldrick, *Acta Crystallogr.* **2012**, *A68*, 448-451.

[S10] S. Parsons, H. D. Flack, T. Wagner, *Acta Crystallogr.* **2013**, B69, 249-259.
